# Patterns of genetic admixture reveal similar rates of borrowing across diverse scenarios of language contact

**DOI:** 10.1126/sciadv.adv7521

**Published:** 2025-08-29

**Authors:** Anna Graff, Damián E. Blasi, Erik J. Ringen, Vladimir Bajić, Daphné Bavelier, Kentaro K. Shimizu, Brigitte Pakendorf, Chiara Barbieri, Balthasar Bickel

**Affiliations:** ^1^ Institute for the Interdisciplinary Study of Language Evolution (ISLE), University of Zurich, Zurich 8050, Switzerland.; ^2^ Department of Evolutionary Biology and Environmental Studies, University of Zurich, Zurich 8057, Switzerland.; ^3^ Catalan Institute for Research and Advanced Studies (ICREA), Barcelona 08010, Spain.; ^4^ Center for Brain and Cognition, Pompeu Fabra University, Barcelona 08010, Spain.; ^5^ Linguistic Research Infrastructure (LiRI), University of Zurich, Zurich 8050, Switzerland.; ^6^ Human Biology and Primate Evolution, Freie Universität Berlin, Berlin 14195, Germany.; ^7^ Faculté de Psychologie et des Sciences de l’Education, Université de Genève, Geneva 1202, Switzerland.; ^8^ Kihara Institute for Biological Research, Yokohama City University, Yokohama 244-0813, Japan.; ^9^ Laboratoire Dynamique Du Langage, CNRS & Université Lumière Lyon 2, Lyon 69007, France.; ^10^ Dipartimento di Scienze della vita e dell’ambiente, Università degli Studi di Cagliari, Cagliari 09124, Italy.

## Abstract

When speakers of different languages are in contact, they often borrow features like sounds, words, or syntactic patterns from one language to the other. However, the lack of historical data has hampered estimation of this effect at a global scale. We overcome this hurdle by using genetic admixture and shared geohistorical location as a proxy for population contact. We find that language pairs whose speaker populations underwent genetic admixture or that are located in the same geohistorical area exhibit notable similar increases in shared linguistic patterns across world regions and different demographic relationships, suggesting a consistent trend in borrowing rates. At the same time, the effect varies strongly across specific linguistic features. This variation is only partly explained by cognitive differences in lifelong learnability and by social functions of signaling assimilation through borrowing, leaving much randomness in which specific features are borrowed. Additionally, we find that, for some features, admixture decreases sharing, likely reflecting signals of divergence (schismogenesis) under contact.

## Teaser

Human population contact leads to consistently similar rates of linguistic borrowing, but effects vary across linguistic features.

## INTRODUCTION

Unlike their genes, humans transfer cultural traits not only through vertical inheritance but also through horizontal borrowing (also referred to as copying, spread, diffusion, or convergence in linguistics) (*1*–*5*). When populations are in contact with each other, they often adopt or borrow cultural elements such as technologies, beliefs, practices, and various aspects of language (*6*). Such instances of horizontal transfer are made possible by the remarkable learning abilities that characterize humans over the entire life span. In the case of language, these abilities can result in various extents of bilingualism and multilingualism, patterns which provide particularly fertile grounds for borrowing (*7*).

However, the extent to which contact results in borrowing remains heavily debated, with far-reaching consequences for the validity of the tree model for linguistic evolution (*8*–*15*). On the one hand, core vocabulary is persistent in vertical transmission and tends to resist borrowing. Many words are inherited with only slight modification all the way from the root of a language phylogeny to large swathes of its descendants (e.g., the words for mother, *madre*, *Mutter*, and *mère* in the Indo-European daughter languages English, Spanish, German, and French, respectively) so that relationships between languages and groups of languages can readily be detected and reconstructed (*16*, *17*). On the other hand, features of linguistic structure, such as patterns in grammar or phonology, tend to be more unstable over time (*18*–*20*). The distribution of such structural features, as well as the introduction of new concepts and words, is often parsimoniously explained by contact between specific pairs of populations (*21*) or between several populations within a wider geohistorical area (*3*, *22*–*28*).

Borrowing is typically initiated by speakers who have already acquired the basics of their native language; younger children tend to be overly resilient and conservative learners, and they mostly lack the social power to initiate change (*29*–*33*). As a result, borrowing is expected to be more likely in features that remain relatively easy to learn after early childhood. For example, relative to grammatical categories, lexical concepts remain easier to learn even later in life (*34*–*37*). Thus, one might expect lexical concepts to be more borrowable than grammatical categories. Observations about differences in borrowability from case studies support this prediction, with lexical items being more readily transferred than structural features (*3*, *5*, *38*). However, it is unknown to what extent this and similar predictions hold globally and to what extent overall rates of borrowing or the borrowability of specific features instead depend more on the social dynamics underlying specific contact histories, such as imbalances of power or demography when populations are in contact.

While frequent, borrowing is not the only outcome of contact. Anthropologists have long noted that contact can also lead to the opposite, viz. schismogenesis, a process of signaling divergence between populations in contact (*39*). Divergence in language features has often been noted in local case studies (*40*–*43*). In specific cases, like within Austronesian languages, contact has been identified as an important driver of diversification in vocabulary, but less so in grammar (*18*) and only partially so in phonology (*44*). At a global scale, divergence has been systematically quantified only for dialect contact (*45*), finding features of grammatical form to diverge most, and for patterns in language phylogenies inferred from core vocabulary data (*46*), finding bursts of change after language splits.

While language contact enjoys substantial attention, its global study has suffered from severe biases (*47*). Research has focused on post hoc inferences, where the current distribution of some features in some region is most plausibly explained by borrowing during past contact. The evidence for such contact is sometimes independently supported by geohistorical evidence, but, in most parts of the world, this evidence is largely circumstantial. As a result, the extent to which contact shapes linguistic evolution, the degree to which features differ in their probability to be borrowed across multiple social, cultural, and demographic situations, and the net balance between borrowing and divergence under contact remain poorly understood.

Here, we introduce genetic admixture as a proxy for population contact that is independent of language, allowing us to study its effects on language. Specifically, we quantify the effects of genetic contact on linguistic structures and ask whether genetic contact and shared geocultural history (to the extent known) yield similar linguistic outcomes. Our focus is on structural patterns, for example, whether the object follows the verb, as in English “eat apples,” as opposed to languages like Japanese and Nepali, where the object precedes the verb; or whether the difference between “k” and “kh” distinguishes meaning as in Nepali, or not, as in English. We exclude from our study the borrowing of concrete words and grammatical forms (“matter borrowing”) (*5*), such as the fact that the word “pound” was borrowed from Latin into English. We compare the extent of borrowing across specific features and assess differences in borrowability as expected from case studies and research on learnability over the life span.

## RESULTS

### Sampling contact between populations

The motivation to capture contact through genetics relies on the effects of human population admixture. The intense demographic contact leading to an admixed population profile presupposes extensive interaction between adults from previously unconnected groups and entails linguistically mixed families. This creates ample opportunity for contact to affect not only genes but also a wide range of cultural traits, including language. Genes and language features such as words or structural patterns are sometimes transferred together in situations of contact (*48*). For instance, the borrowing of click sounds from Khoisan into Bantu languages in Zambia was coupled with demographic exchange between distant groups after large-scale migrations (*49*, *50*).

**Fig. 1.**
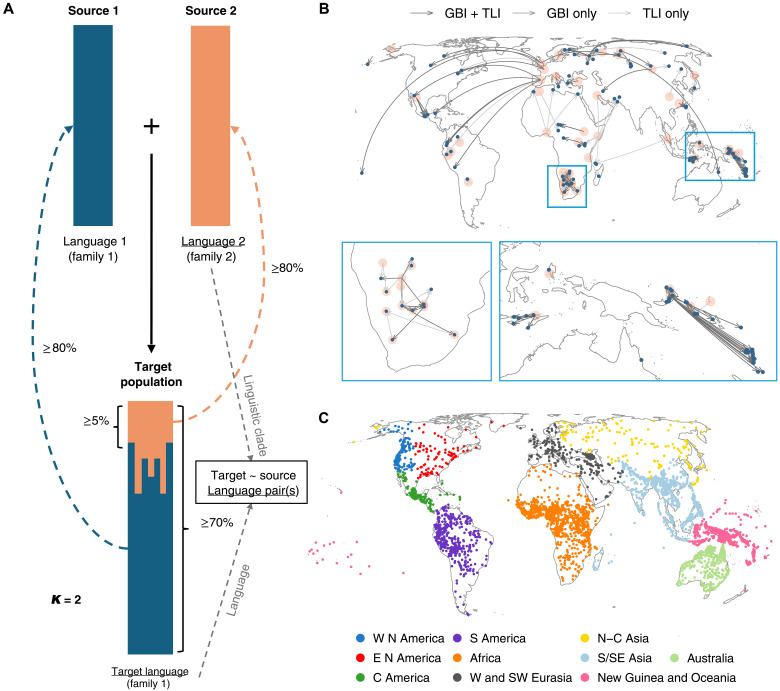
Sampling contact between populations. (**A**) Sampling admixture in populations (solid arrow) if their two largest ancestry components amount to at least 70% of genetic ancestry, of which the minor source contributes at least 5%, and if the admixture is evident through at least five different levels of globally assumed components (*K*, tested from 12 to 30, see also fig. S1A for results at *K* = 12). The two components were manually associated (dashed arrows) with source populations exhibiting the ancestry to at least 80%. Target populations were only considered if their two main ancestries were assigned to populations (source 1 and source 2) speaking languages (language 1 and language 2) from different language families (family 1 and family 2), whereby one of these families (family 1 in this illustration) should be the family of the target language spoken by the target population. Targets were associated (gray arrows) with their now spoken language (“language 1” in the figure) and the source with the phylogenetic clade that best characterizes the language varieties (“language 2”) at the time of contact. To control for shared inheritance, we only sampled pairs where target and source are from different families (family 1 and family 2). (**B**) One hundred twenty-six target-source language pairs. Blue, target languages; orange, source clades (centered on one language for visualization purposes only); TLI and GBI, different linguistic datasets (see the main text). (**C**) Languages from which features were drawn colored by geohistorical area from AUTOTYP (fig. S1B for an alternative). W N America, Western North America; E N America. Eastern North America; C America, Central America; S America, South America; W and SW Eurasia, Western and Southwestern Eurasia; N-C Asia, Northern-Central Asia; S/SE Asia, South/Southeast Asia.

To probe for genetic evidence of contact, we searched for pairwise contact between genetic ancestries that were sufficiently divergent to be distinguishable. In particular, we identified populations with admixture from one ancestry component that is associated with a linguistically unrelated group (Fig. 1A and Materials and Methods). This procedure ensures consistency in the criteria for genetic contact, and it keeps our sample relatively free of confounds from shared linguistic inheritance. However, our procedure is limited by the nonuniform coverage in our sample across the world (Fig. 1B) and by having enough samples to represent each ancestry (*51*). A further limitation is that we cannot cover the impact of cases of pairwise admixture in genetic ancestries underrepresented in our sample, of gradients of admixed ancestries (*52*), and of admixture scenarios with more than two ancestries. Last, the restriction to unrelated language pairs might underestimate both borrowing and divergence effects because these are particularly expected within language families, where structural similarity boosts borrowing (*3*, *15*, *53*–*55*) and shared history boosts divergence (*40*, *45*).

To identify populations with admixture, we ran ADMIXTURE (*56*) and estimated *F*_3_ statistics (*57*) on genomic single-nucleotide polymorphism chip data from an expanded version of GeLaTo (*48*), a database that matches genetic populations to their languages (4768 individuals in 558 populations associated with 373 languages; table S1), and supplemented the results with cases of admixture reported in the literature (fig. S1A and Materials and Methods).

The resulting list of genetically admixed populations covers different geographical and historical scales, including local cases of demic contact, larger Neolithic displacements of farmers and pastoralist groups (*58*), and intercontinental contacts from the past five centuries of population movements (invasions and displacements) associated with European colonialism (*59*) and slave trade (*60*–*62*).

From this list, we derived target-source pairs (*N* =126, table S2) and associated them with languages. For the target, representing a present-day population, we followed the association provided by GeLaTo and the additional literature, associating the population with a single language. For the source population, representing a past population, we have no access to the language(s) with which it was associated at the time of contact. In response, we resorted to the nearest clade of the languages now spoken by present-day proxies for the past source population, assuming that this includes most (often all) of the likely features reconstructible for contact time (Materials and Methods). For example, for the Quechua-Spanish pair, we sampled the source feature states from all available languages within the Romance clade to approximate the 16th century dialect variation at the time when contact began; solely considering modern standard Castilian Spanish would artificially reduce this variation.

We then linked target and source languages to their features from two databases documenting distributions of linguistic patterns, which were curated to remove logical and strong statistical dependencies between features (*63*): GBI (“Grambank Independent”) covering grammatical features, and TLI (“Typology Linked and Independent”) covering a variety of grammatical, lexical, and phonological features (table S3). To control for universal baseline expectations about the presence of features in the target and source languages, we further sampled 300 random pairs of unrelated languages. Because what carries potential for contact effects in multistate features are feature states (e.g., a specific word order such as object-verb versus object-verb) and not the feature by itself (e.g., the order of object and verb in general), we extracted the data separately for every state in features with more than two states, resulting in a total of *n* = 638 feature states (*n* = 202 in GBI and *n* = 481 in TLI).

This procedure focuses on pairwise contact between specific languages. However, contact can also characterize entire networks of languages that jointly evolved in a given geohistorical area (*3*, *23*–*25*, *47*). Although the relevant historical and ethnographic evidence is often (as noted) circumstantial, we allow for this alternative scenario of contact by additionally assigning all sampled languages to areas that have been established as particularly prone to contact (Fig. 1C). We sourced such areas from the AUTOTYP database (*64*) and, for a sensitivity analysis, from Glottolog (*65*, *66*) (fig. S1B) rather than geographical distances between current locations (*67*, *68*), because the areas are less sensitive to recent migrations (*69*–*71*).

We then modeled the probability of a language pair to share states as a function of genetic admixture and areal colocation in a series of Bayesian multilevel logistic regressions (Materials and Methods, figs. S13 to S32, and tables S4 to S23). Model comparison with approximate leave-one-out cross-validation (*72*) suggests that a model with both genetic and areal predictors strongly outperforms models with only one of these predictors (fig. S2, all ${∆}_{\text{elpd}}$ > 260±46).

### Genetic contact and areas increase the probability of sharing feature states

**Fig. 2.**
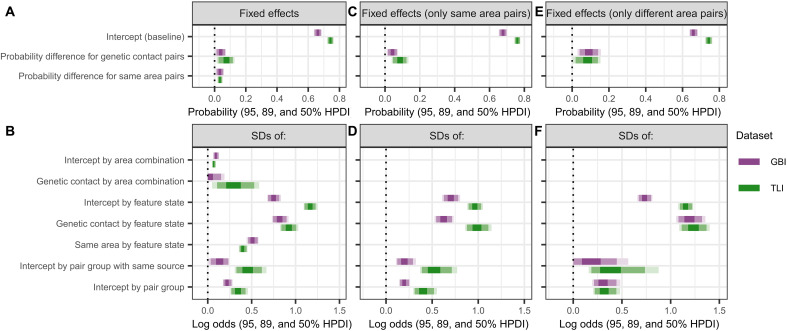
Posterior predictions reveal a robust effect of structural convergence across different contact conditions. (**A** and **B**) In the best-fitting model considering all language pairs, the probability of sharing feature states decisively increases in contact compared to a random baseline (Δ*P* > 0 in more than 99% of the posterior probabilities), across two linguistic datasets (GBI focusing on grammatical and lexical features, TLI additionally including phonological features) (A), but there are substantial SDs by feature state (B). These effects are similar when considering only cases of genetic contact within the same area (**C** and **D**), and when considering only cases of genetic contact between different areas (**E** and **F**).

The best-fitting model shows that both genetically and areally defined contacts increase state sharing (Fig. 2A). Genetic contact increases sharing probabilities by a posterior mean of 3.9% in the GBI dataset [89% highest posterior density interval (HPDI) = [1.2%, 6.7%], *P*($\hat{\beta }$ > 0) = 0.99] and 7.2% in the TLI dataset [89% HPDI = [2.6%, 11.7%]; *P*($\hat{\beta }$ > 0) = 0.99]. Belonging to the same area increases sharing probabilities by a posterior mean of 3.4% in GBI [89% HPDI = [1.3%, 5.5%], *P*($\hat{\beta }$ > 0) = 0.99] and of 3.5% in TLI [89% HPDI = [2.2%, 4.9%], *P*($\hat{\beta }$ > 0) = 1.00]. All results are robust against the alternative area definition from Glottolog and against a sensitivity analysis excluding language pairs not associated with a negative and significant *F*_3_ statistic (fig. S3) (*57*). The effects are relatively strong given that the baseline expectations are well above chance (i.e., a 50% probability of sharing a state) and leave only limited room for probability increases (with all posterior mean sharing probabilities in the baseline higher than 64.0%; 89% HPDI = [64.0%, 68.1%] for GBI and 89% HPDI = [72.5%, 75.8%] for TLI). In particular, in the case of genetic contact, we find strong evidence for borrowing (HPD > 89%) even in cases where the universal baseline probability of sharing is high and the effects, therefore, remain small.

The total distribution inside an area might also influence the chances of detecting borrowing under genetic contact when sampling pairs from within that area (e.g., Bantu and Khoisan languages within Africa) as opposed to between different areas (e.g., Iberian and Quechuan languages between Europe and South America). This possibility is important because, beyond differences in demographic particulars and geographical scale, the two scenarios potentially correlate with different histories: Genetic contact between different areas in our sample predominantly involves cases associated with European colonialism, with more recent timing and stronger demographic imbalances than most other cases of genetic contact, and genetic contact within the same area tends to exclude these. However, quantifying the effect of genetic contact separately for contact within areas (Fig. 2, C and D) versus between areas (Fig. 2, E and F) reveals the same overall trends: Within-area genetic contact results in a mean posterior probability increase of 4.3% [89% HPDI = [1.7%, 6.9%], *P*($\hat{\beta }$ > 0) = 0.99] in the GBI dataset and a mean posterior probability increase of 8.5% [89% HPDI = [4.5%, 12.3%], *P*($\hat{\beta }$ > 0) = 1.00] in the TLI dataset, and between-area genetic contact results in a mean posterior probability increase of 8.9% [89% HPDI = [4.1%, 14.1%], *P*($\hat{\beta }$ > 0) = 0.99] in the GBI dataset and a mean posterior probability increase of 7.8% [89% HPDI = [2.0%, 14.0%], *P*($\hat{\beta }$ > 0) = 0.97] in the TLI dataset.

Globally, contact therefore robustly favors effects of structural borrowing over effects of divergence, and this holds to the same extent when considering genetic contact associated with different demographic conditions, when considering areal contact and across all considered conditions. This is confirmed by a meta-analysis over the probability differences for the fixed effects (fig. S4 and Materials and Methods): The shortest credible interval to include zero is the 80% HPDI for model type (i.e., the different demographic conditions shown in Fig. 2, A versus C versus E), the 19% HPDI for main versus sensitivity analyses, the 79% HPDI for dataset (GBI versus TLI), and the 88% HPDI for genetic versus areal contact.

### Effects of contact vary across feature states

**Fig. 3.**
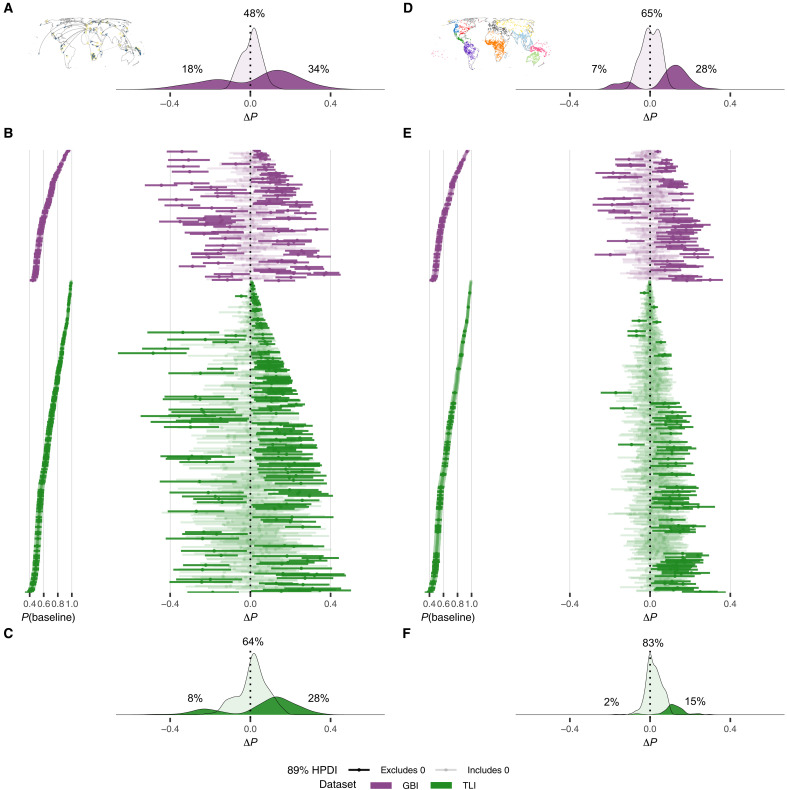
Posterior baseline sharing probability per feature state and posterior Δ*P* for each feature state under contact (ordered by dataset and effect size). (**A** to **C**) As a result of genetic admixture versus (**D** to **F**) colocation in a geohistorical area. For alternatively ordered visualizations of posterior Δ*P*, see fig. S7A. [(A) and (C)] Density plots of states with 89% HPD excluding versus including zero in GBI and TLI under genetic contact, [(D) and (F)] under areal colocation. [(B) and (E)] Interval plots per feature state, ordered by increasing posterior baseline. For details, see tables S24 and S25. There are more features with decreases in sharing probability (Δ*P* < 0) in genetic than areal contact (density plots of features with 89% HPD excluding versus including 0), suggesting specific effects of divergence in addition to borrowing. Although states with higher baseline sharing probabilities have less scope for increased rates of borrowing under contact, we detect borrowing for states across the range of baseline sharing probabilities.

The slightly different effect sizes of genetic contact on the features included in the GBI and TLI datasets (Fig. 2, A and C) suggest a fair amount of variation driven by the specific features coded in each dataset. This is confirmed by the relatively large SDs of feature states (Fig. 2, B, D, and F; all posterior mean SD > 0.40 ± 0.06 in log odds and two SEs). Figure 3 shows the estimated effects in terms of probability differences for all 683 states (for details, see tables S24 and S25). In addition to states with excess sharing under contact, i.e., borrowing [positive differences; 34 and 28% of states in genetic contact for GBI (Fig. 3A) and TLI (Fig. 3C), respectively; 28 and 15% of states in areal contact for GBI (Fig. 3D) and TLI (Fig. 3F), respectively], there is a substantial number of states that are unaffected by contact (with zero included in the 89% HPDI; 48 and 64% of states in genetic contact for GBI and TLI, respectively; 65 and 83% of states in areal contact for GBI and TLI, respectively). Additionally, to a lesser but still noticeable extent, there are states with decreased sharing probabilities, i.e., cases of divergence under genetic contact (18% of states in GBI and 8% in TLI; Fig. 2C). Under areally defined contact, divergence effects are much rarer (7% of states in GBI and 2% in TLI; Fig. 2B). This might reflect the fact that divergence is intrinsically difficult to discover in areas because the total proportion of shared states in an area can be high even if most geographically adjacent languages show local divergence. Again, a separate quantification of the effect of genetic contact between versus within areas (fig. S5, C and D) revealed the same overall trends as without the distinction (fig. S5A).

Intriguingly, while the global effects are similar across all types of contact, they vary at the level of individual feature states: The feature state that shows most borrowing in areal contact shows divergence or no difference in genetic contact (regardless of whether this is defined globally or within or between areas). Conversely, the feature state that shows most divergence in areal contact shows borrowing or no difference in genetic contact (see fig. S5 and table S24 for details). These observations hold beyond the single-most extreme cases of borrowing and divergence, and they are very similar in the sensitivity analysis (figs. S6 to S8 and table S25). They suggest that specific feature states differ in how likely they are to act as carriers for signaling social divergence versus convergence in the form of schismogenesis or borrowing in different types of contact (*45*).

### Meta-analyses highlight differences between domains of language

Feature states differ in how they react to contact, but do these differences match the hierarchies in the relative borrowability of linguistic patterns that have been proposed in the past on the basis of case studies? Do they align with expectations about the relative ease of learning different types of features across the life span? To answer these questions, we conducted further meta-analyses comparing contact effects across classes of features as defined in the GBI and TLI datasets (table S3) (*63*). Table 1 provides definitions and examples for each feature class. While motivated by considerations of linguistic analysis, they approximate some of the distinctions for which one would expect differences in borrowability and learnability based on previous reports (*3*, *5*, *23*, *34*–*37*).

**Table 1.** Feature classifications for meta-analyses. Definitions and examples based on (*63*).

Name	Definition	Examples
lexical semantics	Structuring of lexical meaning	Colexifications (e.g., same word for “arm” and “hand” like in Czech)
lexical classes	Formal, morphosyntactically relevant information in the lexicon	Presence of verb classes (e.g., conjugation classes in Italian where some verbs have third person form like *am**a*** “loves,” while others have third person form like *cred**e*** “believes”), grammatical gender (e.g., distinctions like masculine ***il*** *problema* “the problem” versus feminine ***la*** *macchina* “the machine”)
grammatical categories	Presence and nature of semantic notions in grammar (not their concrete expression)	Presence of past tense (e.g., English *work**ed***), evidentiality (e.g., Turkish *gel**miş*** “I hear/infer she came”)
linear order	Ordering of sentence elements	Position of the object relative to the verb (e.g., *eat rice* like in English or *bhāt khānu* “rice eat” like in Nepali)
other grammar	All other formal aspects of grammar	Past tense marking at the beginning (e.g., Swahili *a**li**soma* “studied”) versus at the end of verb forms (e.g., English *work**ed***), type of relative clause formation (e.g., *the woman **who** works*, with pronoun “who” in English versus *çalış**an** kadın* with ending *-an* in Turkish)
phonology	Patterns and rules in the sound system	(Any features relating to the following three types: prosody, vocalic, and consonantal)
prosody	Rhythm, stress, and intonation	Stress placement (e.g., always on the first syllable of a verb as in Hungarian versus variable as in English)
vocalic	Features of the vowel system	Vowel nasalization (e.g., the distinction between *bon* “good” and *beau* “beautiful” in French)
consonantal	Features of the consonant system	Presence of aspiration (e.g., the distinction between *kām* “work” and *khām* “envelope” in Nepali)

**Fig. 4.**
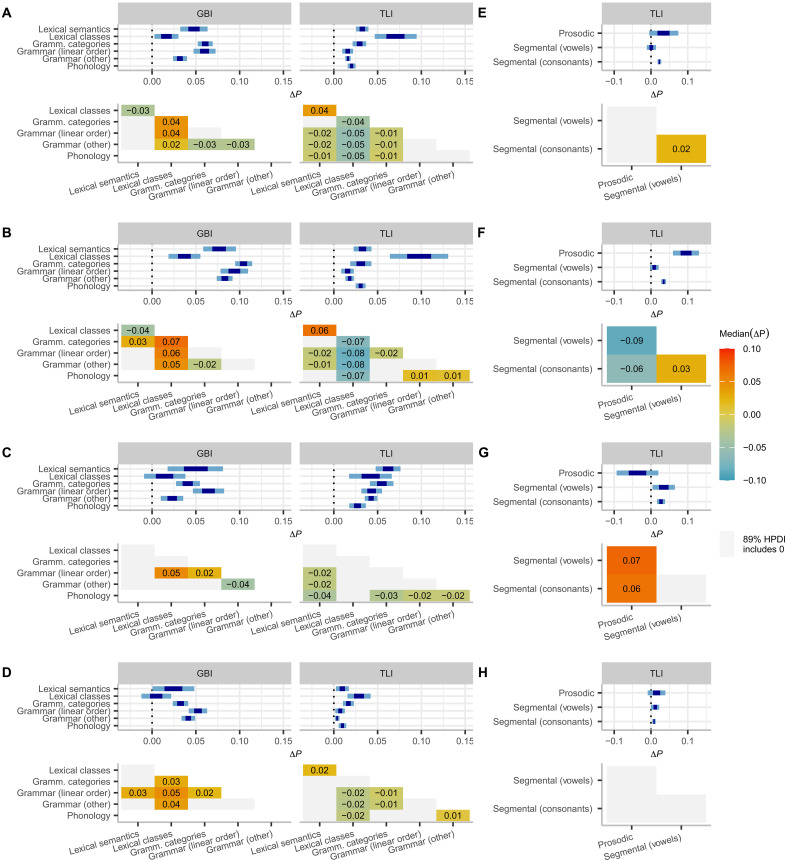
Meta-analyses of contact effects under different types of contact scenarios. (**A** to **D**) Across domains of language of the TLI and GBI dataset; (**E** to **H**) across phonological features (TLI dataset only, the GBI dataset does not include phonological data). [(A) and (E)] Genetic contact, all pairs; [(B) and (F)] genetic contact, different area pairs only; [(C) and (G)] genetic contact, same area pairs only; [(D) and (H)] colocation in the same area. Intervals show the 50 and 89% credible intervals of the combined effects per domain. Heatmaps show the median posterior difference between domains, with gray shading for differences whose 89% HPDI includes zero. For example, lexical class features in the GBI dataset are 3% less likely to be shared under genetic contact than features of lexical semantics (A).

Our results are only partially consistent with expectations. Most in line with the results of previous suggestions is our finding that features of linear order show similar or higher borrowing probabilities (Δ*P* > 0) compared to other aspects of grammar (Fig. 4, A to D; see fig. S9 for details and fig. S11 for the sensitivity analysis). For instance, in GBI, linear order shows a 3% higher median posterior probability of sharing states under genetic contact (Fig. 4A) while there is no evidence for a difference under areal contact (gray-shaded cell, Fig. 4D). This is largely in line with results from case studies (*3*, *23*), with findings that features of linear order tend to be underexploited for schismogenesis (*45*) and with its relatively persistent learnability over age (*73*). However, the effect is not as robust across conditions as one might expect, and it is only supported with high posterior probabilities in the GBI dataset (Fig. 4, A to H, and fig. S10). Another finding that seems to confirm previous reports (*68*, *74*) is higher or similar borrowing probabilities for consonantal over vocalic features, but strong evidence is again limited to only some conditions (Fig. 4, E to H, and figs. S10 and S12). Last, features relating to lexical semantics show borrowing probabilities in a similar or higher range than grammar (linear order and other grammar) and phonology, but there is an exception to this trend in the same-area condition in the GBI dataset (Fig. 4D) where lexical semantics shows lower borrowing probability than linear order. These observations seem to partially confirm the notion that new lexical concepts remain easier to learn after childhood than other structural aspects of language. However, the difference is yet again neither as pronounced nor as consistent as one might expect on the basis of previous research.

At the same time, we note that lexical classes show higher or similar borrowing probabilities than lexical semantics in the TLI but not in the GBI dataset. Further, grammatical categories show borrowing probabilities in a similar range (gray-shaded cells; Fig. 4, A, C, and D) as lexical semantics or even higher (Fig. 4B), and, like lexical semantics, they outrank linear order, other grammar, and phonology, with median posterior differences of 1 to 3%, again with an exception in the same-area condition of the GBI dataset and also in the within-area genetic condition of the GBI dataset (Fig. 4, C and D). These observations are unexpected given that grammatical categories and lexical classes are difficult to acquire after the critical period of early childhood (*36*, *37*, *75*–*80*), i.e., at an age at which borrowing is most likely to be initiated. While previous research would therefore predict low borrowability for these features, we observe relatively high borrowabilities.

Several explanations for this unexpected finding are possible. One possibility is that adult learning of categories (e.g., the existence of a past tense) as opposed to their formal marking (e.g., that past tense is marked at the beginning or at the end of a verb; Table 1) is easier than suspected or that this is specifically the case for the categories coded in our data and less so for those that have been commonly studied in (predominantly) European languages. Another possibility is that our categorization of features might not pick up the distinctions that matter for learnability. For example, it is possible that the opposite effects in lexical classes in the GBI and TLI datasets stem from differences in parts of speech, because the GBI dataset focuses more on nominal than verbal classes (table S25). Yet another possibility is that children might be more actively involved in borrowing than commonly assumed. This might be particularly important in complex and intensive contact situations such as in genetic contact between areas (Fig. 4B), a condition where our data mostly rest on contact associated with European colonialism and where the probability of borrowing grammatical categories is decisively higher than borrowing lexical semantics, at least in the GBI dataset.

While we cannot at present quantify the relative contributions of these possibilities to an overall explanation of our findings, the social conditions of colonial contact might be specifically relevant for capturing effects we find in features of prosody (Fig. 4, F and G). Prosody—the rhythmic, stress, and intonational properties of speech—is a particularly important marker of social belonging and differentiation (*81*, *82*). We find higher borrowing probabilities for prosody than for other aspects of phonology in genetic contact between areas (Fig. 4F). These are conditions with strong power imbalances associated with colonialism, exerting high pressure for assimilation due to prestige hierarchies (*83*). By contrast, features of prosody show lower borrowing probabilities than other aspects of phonology in genetic contact within areas. In this contact condition, prosodic features are even less likely to be shared than in the baseline condition, indicating divergence (Fig. 4G). This might be due to the function of prosodic features as markers of divergence in socially more balanced contact situations.

## DISCUSSION

While our study targets only contact between languages from different families and invites extensions to data from related languages, our results suggest that the extent of borrowing is notable similar across all the contact conditions considered. In particular, the similarity extends to between-area contact. This is consistent with observations that regular patterns of linguistic transmission persist even in the special demographic and social dynamic of such contact, as in the case of creole formation (*84*). But what drives the regularity and similarity of borrowing rates?

Received scholarship points to universal principles of borrowability across features, but our meta-analyses cast doubt on this. We find that globally fixed hierarchies of pattern borrowing mostly fail to capture the outcomes of language contact. While some of the differences in borrowing probability (linear order versus other grammar, consonants versus vowels, and lexical semantics versus other features) partially match expectations, the evidence is not robust across conditions and datasets for most of the predictions that our data allow us to test. Moreover, beyond these domains, we find glaring differences in the specific linguistic feature states that tend to be borrowed in contact. Again, different contact conditions produce different relative rankings of borrowability, possibly reflecting the contingencies of sociolinguistic history in each contact pair or geohistorical area (*55*, *81*, *85*).

An alternative explanation for consistently similar borrowing rates lies in a potentially general, stationary and self-limiting rate of horizontal spread of linguistic patterns through contact, which is constrained not to substantially disrupt vertical transmission pathways of linguistic evolution. Testing whether there are bounds to this rate requires larger and more uniform samples and more targeted research on how borrowing events between individual speakers lead to population-level changes (*86*).

Our analyses further find that only few structural features are globally preferred signals of social distinction between unrelated languages. This is consistent with the observation that divergence between dialects is more commonly signaled by differences in word choice than in structural features (*45*). Our findings add features in prosody, such as patterns of word stress, as favored carriers of social distinction in conditions of balanced contact.

Together, these results paint a complex picture for the past and future of the world’s languages. Extensive demographic contact would not only have contributed to language loss but also critically affected distributions of linguistic features, promoting the erosion of structural linguistic diversity among surviving languages. We anticipate that this dynamic will continue to jeopardize linguistic diversity beyond language loss (*87*) in our increasingly globalized world confronted with, among others, the consequences of land use expansions (*88*) and demographic displacements induced by climate change (*89*).

## MATERIALS AND METHODS

### Experimental design

The genetic analysis relies on genomic data from an expanded version of the GeLaTo dataset (*48*, *90*). The database includes 4768 unrelated individuals in 558 genetic populations (median: eight individuals) representing 373 languages. Each genetic population is assigned to a language with a Glottocode (*66*), following curating criteria based on anthropological and ethnolinguistic information available from the original genetic publication (*48*). These populations are listed in table S1. The literature review for additional admixture cases identified 45 additional instances of contact compatible with the criteria used through our ADMIXTURE run on genomic data (*61*, *91*–*104*). The structural linguistic data used in this research draws from the full “statistical” curations of the GBI and TLI datasets (*63*). These datasets were previously curated to merge structural linguistic data from standard databases of linguistic diversity in a way that minimizes the amount of missing data and reduces logical and strong universal statistical dependencies between features (*63*), making it suitable for models like ours that assume conditional independence of the response. For area coding, we used the 10 continent-sized areas (Fig. 1C) from AUTOTYP (*64*) in the main analysis and the six macroareas (fig. S1B) from Glottolog (*66*) in the sensitivity analysis.

For the genetic analysis, we ran the ADMIXTURE software (*56*) assuming *K* = 2 to *K* = 30 ancestries with 20 runs per each *K*, retaining the run with the highest likelihood per *K*. The smallest cross-validation errors are found at *K* = 23 and *K* = 22. After checking the likelihood profiles and visually inspecting the consistency of the runs, we selected those from *K* = 12 to *K* = 30, because the lowest *K* (*K* = 12) returned recognizable blocks of ancestry (fig. S1A). Previous studies using ADMIXTURE with global datasets but fewer population samples recognized solid blocks of ancestry at *K* = 5 (*105*), *K* = 8 (*106*), and *K* = 10 (*94*), but these studies did not explore higher values of *K*. We then identified populations as admixed if their two largest ancestry components amounted to at least 70% of genetic ancestry, of which the smaller source contributed at least 5%. Minor sources contributing less than 5% of ancestry were considered so small that they would reflect noise. We furthermore required that these thresholds hold through at least five different levels of *K*: If they were admixed for fewer than 5 levels of *K*, then we disregarded the admixture signal for the population as insufficiently consistent.

To identify the two source populations for the ancestry components in the admixed populations, we approximated these source populations for each *K* by populations available in the GeLaTo sample. We did this by flagging all populations that incorporate the admixture component in question at ≥80%. For each admixed (target) population, we had a set of possible source 1 populations denoting the larger admixture component and a set of possible source 2 populations denoting the smaller admixture component. Our final filtering step was to list all possible combinations of source 1 and source 2, where one of the two sources was represented by a population speaking a language of the same language family as the target population and the other represented a population speaking an unrelated language. This resulted in a list of genetic triplets consisting of a target, a source 1 and a source 2 population. For each triplet, we logged the following information: the number of distinct *K* over which the target population was admixed; for how many of these *K* the source 1 population appeared as a match for the larger admixture component; for how many of these *K* the source 2 population appeared as a match for the smaller admixture component; and availability of structural linguistic data for the target population as well as each of the source populations. The target populations along with their unrelated source population were considered potential candidates for further analysis are retrievable from Zenodo (doi.org/10.5281/zenodo.15263706, output/longlists/longlist_for_manual_curation.csv). Of all 3506 candidates considered under the 70% admixture threshold, 81% also held under a more conservative threshold of 80 and 56% even held under a threshold of 90%.

This list of candidate triplets was then filtered to yield language pairs for which we had linguistic feature data available in GBI and TLI, with a (single) target language denoting the target population and a set of languages representing one or more languages in a clade unrelated to the target language associated with the source population. For some populations, we used closely related proxy languages in the linguistic database to increase data coverage. Table S1 lists all relevant populations’ language assignments in the original GeLaTo database and our mappings to GBI and TLI.

Many of the candidate triplets were spurious in that they only appeared inconsistently across a small number of *K*. For instance, Greek populations appeared admixed at the 70% threshold for five different values of *K.* However, only one of these *K*, namely, *K* = 12, marked the Greek as admixed with a non–Indo-European source (in this case, Afro-Asiatic). This signal was too weak to be included for analysis. Similarly, many candidates were dubious given external knowledge of population genetics in the relevant world region. For instance, the ancestry that we know to be Indo-European that acted as a source for many admixture events in South America was often also additionally labeled as a Uralic ancestry component. In these cases, we did not include contact pairs involving Uralic languages given our historical knowledge of imperialism and the colonization of the Americas. Last, in some cases, we were not able to assign languages to the admixture sources with any reasonable degree of confidence, or there was no reasonable linguistic data available for the languages. Overall, our curation yielded a set of 81 genetic contact pairs, to which we added 45 further pairs based on a literature review. Each pair set was assigned a unique pair ID, which was nested within 39 broad pair IDs that group together pairs with the same admixture source; for example, a single broad pair ID (broad pair ID 21) encompassed all seven instances of Spanish admixture into Central and South American populations (pair IDs 21.01, 21.02, 21.03, 21.04, 21.05, 21.06, and 21.07). For those 81 genetic contact pairs derived from our ADMIXTURE analysis, we sought to perform an additional statistical test for admixture using Patterson’s *F*_3_ statistic (*57*). Negative *F*_3_ values are indicative of admixture, with *z*-scores < −3 representing the conventional significance threshold, while positive *F*_3_ values do not prove lack of admixture. For six pairs, computing *F*_3_ was not possible because they were derived from genetic triplets where the source 1 population corresponded to the target population. For the remaining 75 language pairs, we computed the *F*_3_ statistics and corresponding *z*-scores for all associated population triplets, recording the lowest associated *F*_3_ statistic and corresponding *z*-score in the final pair list and whether or not these values are further indicative of admixture according to conventional significance thresholds. This final list is available as table S2.

We then intersected the pairs with linguistic features from GBI and TLI. Specifically, for each dataset, we assembled all available language pairs whose speaker populations are admixed. A single pair ID could thereby be represented by multiple language pairs, if several languages and/or dialects from the specified source clade were available for a given feature. In these cases, pair IDs and broad pair IDs handled relatedness between these potential source languages. For the GBI features, this yielded 348 language pairs; for the TLI features, it yielded 818 language pairs.

For every feature matched to at least one pair ID, we extracted the data for all pair sets. We recorded each target language’s and each source language’s state for the feature and recorded whether the pair has the same state or not. We additionally recorded whether the language pair is from the same continent-sized AUTOTYP area or (for the sensitivity analysis) Glottolog macroarea. To these language pairs, we added 300 randomly drawn language pairs for every feature to act as a baseline. For every feature with more than two states, we drew pairs for every state separately because feature states (e.g., a specific word order such as subject-object-verb versus object-verb-subject) rather than features by themselves (e.g., the word order of subject, object, and verb generally) carry potential for contact effects in multistate features.

### Statistical analysis

Subsets of the resulting language pair data then served as input for a series of five Bayesian multilevel logistic regressions (m1 to m5), once for the main analysis and once for the sensitivity analysis against an alternative definition of geohistorical areas. Additionally, we also fit the main model m1 excluding those language pairs for which we did not find independent support for admixture in our *F*_3_ analysis. We implemented the models (as defined in the next section) in R’s brms (*107*) interface to Stan (*108*). Because the features from these datasets are only curated for reduced statistically dependence within but not across datasets, we fitted every model on the GBI and TLI data, separately.

All models allow the global intercept (fixed effect) to vary by feature state (random effect). Given our statewise (binarized) approach to modeling the features, the global intercept is at least 0.5 on the probability scale, motivating a prior with a positive mean for it. The models including information on areal colocation additionally include a global slope for the effect of areal contact that we allow to vary again by feature state, and also varying intercepts and slopes for each area combination, accounting for varying degrees of baseline similarity and of different contact effects among pairs, depending on the areas that they are from. Models including genetic information included global slopes for the effect of genetic contact, which were allowed to vary by feature state as well as by each instance of contact labeled under the same pair ID. These latter slopes were nested within their broad pair ID. Together, these varying slopes account for structuring among different contact pairs. In the models including information on both areal and genetic contact, we also included varying slopes for genetic contact by area combination of the languages involved.

We ran prior and posterior predictive checks for every fitted model (figs. S13 to S32). We performed model comparison with leave-one-out cross-validation using Pareto-smoothed importance sampling from the posterior, using the loo package (*109*).

For a robustness analysis, we drew posterior samples of the main genetic and areal effects from the fitted statistical models m1, m4, and m5 for both datasets and for both the main and the sensitivity analyses. Using the mean and SD of each effect estimate, we then performed a meta-analysis (see the next section for model definition). We report average marginal effects for each contrast, using the marginaleffects package (*110*).

To report state sharing, we drew posterior samples from the fitted statistical models m1, m4, and m5 and computed probability differences for all feature states under genetic contact (in m1, m4, and m5) compared to the baseline or under the same area condition (in m1) compared to the different area condition. Using the mean and SD of each difference, we then performed meta-analyses (see the next section for model definitions) with varying slopes by feature state group, using a categorical predictor to subsume feature states of the same group. All data and scripts necessary to reproduce all elements of the analysis are available on Zenodo (doi.org/10.5281/zenodo.15263706).

### Model specifications

#### Combined model, with both genetic and information on areal colocation (m1)

$${\text{same\_state}}_{i}∼\text{Bernoulli}\left(\pi_{i}\right)$$ (1)

$$\pi_{i}=\text{logi}t^{-1}(\eta_{i})$$ (2)

$$\begin{array}{c} \eta_{i}=\alpha +\alpha_{\text{STATE}\left[i\right]}+\alpha_{\text{AA}\left[i\right]}+\left(\beta_{1}+\beta_{A,\text{STATE}\left[i\right]}\right)\times A_{i}\\ +\left(\beta_{2}+\beta_{G,\text{STATE}\left[i\right]}+\beta_{\text{AA}\left[i\right]}+\beta_{\text{BROAD\_ID}/\text{PAIR\_ID}\left[i\right]}\right)\times G_{i} \end{array}$$ (3)

(αAAβAA)∼mvN(00),ΣAA (4)

$$\Sigma_{\text{AA}}=\left(\begin{array}{cc} \sigma_{\alpha_{\text{AA}}}^{2} & \sigma_{\alpha_{\text{AA}}}\sigma_{\beta_{\text{AA}}}\rho \\ \sigma_{\alpha_{\text{AA}}}\sigma_{\beta_{\text{AA}}}\rho & \sigma_{\beta_{\text{AA}}}^{2} \end{array}\right)$$ (5)

$$\left(\begin{array}{c} \alpha_{\text{STATE}}\\ \beta_{A,\text{STATE}}\\ \beta_{G,\text{STATE}} \end{array}\right)∼\mathrm{mvN}\left[\;(\begin{array}{c} 0\\ 0\\ 0 \end{array}),\Sigma_{\text{STATE}}\right]$$ (6)

$$\begin{array}{c} \Sigma_{\text{STATE}}=\left(\begin{array}{ccc} \sigma_{\alpha_{\text{STATE}}}^{2} & \sigma_{\alpha_{\text{STATE}}}\sigma_{\beta_{A,\text{STATE}}}\rho & \sigma_{\alpha_{\text{STATE}}}\sigma_{\beta_{G,\text{STATE}}}\rho \\ \sigma_{\alpha_{\text{STATE}}}\sigma_{\beta_{A,\text{STATE}}}\rho & \sigma_{\beta_{A,\text{STATE}}}^{2} & \sigma_{\beta_{A,\text{STATE}}}\sigma_{\beta_{G,\text{STATE}}}\rho \\ \sigma_{\alpha_{\text{STATE}}}\sigma_{\beta_{G,\text{STATE}}}\rho & \sigma_{\beta_{A,\text{STATE}}}\sigma_{\beta_{G,\text{STATE}}}\rho & \sigma_{\beta_{G,\text{STATE}}}^{2} \end{array}\right) \end{array}$$ (7)

α indicates the intercept, and β_1_ indicates the fixed effect of a language pair being from the same area (1) or not (0) (*A*). β_2_ indicates the fixed effect of a language pair representing a genetic contact pair (1) or a baseline pair (0) (*G*).

The terms $\alpha_{\text{STATE}\left[i\right]}$ and $\alpha_{\text{AA}\left[i\right]}$ indicate that the intercept can vary by state and area combination. $\beta_{A,\text{STATE}\left[i\right]}$ and $\beta_{G,\text{STATE}\left[i\right]}$ indicate that the both the area and the genetic contact slope can vary by state. $\beta_{\text{AA}\left[i\right]}$ and $\beta_{\text{BROAD\_ID}/\text{PAIR\_ID}\left[i\right]}$ indicate that the genetic contact slope can further vary by area combination and by each contact instance (*pair_id*), nested within *broad_id* (grouping contact instances with the same source).

We set the following priors on model parameters

α∼N(0.75,0.5) (8)

$$\beta_{1,}\;\beta_{2,}∼N\left(0,1.5\right)$$ (9)

$$\alpha_{\text{STATE}\left[i\right]},\beta_{A,\text{STATE}\left[i\right]},\beta_{G,\text{STATE}\left[i\right]}∼N\left(0,\sigma_{\text{STATE}\left[i\right]}\right)$$ (10)

$$\alpha_{\text{AA}\left[i\right]},\beta_{\text{AA}\left[i\right]}∼N\left(0,\sigma_{\text{AA}\left[i\right]}\right)$$ (11)

$$\beta_{\text{BROAD\_ID}/\text{PAIR\_ID}\left[i\right]}∼N\left(0,\sigma_{\text{BROAD\_ID}/\text{PAIR\_ID}\left[i\right]}\right)$$ (12)

$$\sigma_{\text{STATE}\left[i\right]},\sigma_{\text{AA}\left[i\right],}\sigma_{\text{BROAD\_ID}/\text{PAIR\_ID}\left[i\right]}∼N\left(0,2\right)$$ (13)

$$R_{\text{AA}},R_{\text{STATE}}∼\mathrm{LKJ}\left(2\right)$$ (14)

The intercept is assumed to follow a normal prior with mean of 0.75 and SD of 0.5. Fixed effects are each assumed to follow a normal prior with mean of 0 and SD of 1.5. Each varying effect individually follows a normal prior with mean of 0 and SD of 2. Jointly, the varying effects by area combination follow multivariate normal priors with mean 0 and variance-covariance $\Sigma_{\text{AA}}$ . The effects by feature state follow multivariate normal priors with mean of 0 and variance-covariance $\Sigma_{\text{STATE}}$ . The covariance matrices are decomposed into a prior SD vector and a correlation matrix **R**. These **R** matrices are individually drawn from an LKJ(2) prior.

This model (m1) was fitted using feature states from GBI (*n* = 202) and TLI (*n* = 481), separately.

#### Model with areal information only (m2)

$${\text{same\_state}}_{i}∼\text{Bernoulli}\left(\pi_{i}\right)$$ (15)

$$\pi_{i}={\text{logit}}^{-1}(\eta_{i})$$ (16)

$$\eta_{i}=\alpha +\alpha_{\text{STATE}\left[i\right]}+\alpha_{\text{AA}\left[i\right]}+\left(\beta_{1}+\beta_{\text{STATE}\left[i\right]}\right)\times A_{i}$$ (17)

(αSTATEβSTATE)∼mvN(00),ΣSTATE (18)

$$\Sigma_{\text{STATE}}=\left(\begin{array}{cc} \sigma_{\alpha_{\text{STATE}}}^{2} & \sigma_{\alpha_{\text{STATE}}}\sigma_{\beta_{\text{STATE}}}\rho \\ \sigma_{\alpha_{\text{STATE}}}\sigma_{\beta_{\text{STATE}}}\rho & \sigma_{\beta_{\text{STATE}}}^{2} \end{array}\right)$$ (19)

The priors drawn are the same as in model m1.

α indicates the intercept, and β_1_ indicates the fixed effect of a language pair being from the same area (1) or not (0) (*A*). The terms $\alpha_{\text{STATE}\left[i\right]}$ and $\alpha_{\text{AA}\left[i\right]}$ indicate that the intercept can vary by feature state and area combination. $\beta_{\text{STATE}\left[i\right]}$ indicates that the area slope can vary by feature state.

This model (m2) was fitted using feature states from GBI (*n* = 202) and TLI (*n* = 481), separately.

#### Model with genetic information only (m3, m4, and m5)

$${\text{same\_state}}_{i}∼\text{Bernoulli}\left(\pi_{i}\right)$$ (20)

$$\pi_{i}={\text{logit}}^{-1}(\eta_{i})$$ (21)

$$\eta_{i}=\alpha +\alpha_{\text{STATE}\left[i\right]}+\left(\beta_{1}+\beta_{\text{STATE}\left[i\right]}+\beta_{\text{BROAD\_ID}/\text{PAIR\_ID}\left[i\right]}\right)\times G_{i}$$ (22)

(αSTATEβSTATE)∼mvN(00),ΣSTATE (23)

$$\begin{array}{c} \Sigma_{\text{STATE}}=\left(\begin{array}{cc} \sigma_{\alpha_{\text{STATE}}}^{2} & \sigma_{\alpha_{\text{STATE}}}\sigma_{\beta_{\text{STATE}}}\rho \\ \sigma_{\alpha_{\text{STATE}}}\sigma_{\beta_{\text{STATE}}}\rho & \sigma_{\beta_{\text{STATE}}}^{2} \end{array}\right) \end{array}$$ (24)

The priors drawn are the same as in model m1.

α indicates the intercept, and β_1_ indicates the fixed effect of a language pair representing a genetic contact pair (1) or a baseline pair (0) (*G*). The term $\alpha_{\text{STATE}\left[i\right]}$ indicates that the intercept can vary by feature state. $\beta_{\text{STATE}\left[i\right]}$ indicates that the genetic contact slope can vary by feature state. The random slope $\beta_{\text{BROAD\_ID}/\text{PAIR\_ID}\left[i\right]}$ allows for contact effects to vary for each contact instance (*pair_id*), nested within *broad_id* (grouping contact instances with the same source).

This model was fitted once on available pairs (m3), once on only pairs of languages from the same area (m4), and once on only pairs from different areas (m5). In each case, separate models were fitted for GBI (*n* = 202) and TLI (*n* = 481) feature states.

#### Main effect meta-analysis model

$$y_{i}∼N\left(\mu_{i},\sigma_{i}\right)$$ (25)

$$\mu_{i}=\alpha +\beta_{1}\times m_{i}+\beta_{2}\times s_{i}+\beta_{3}\times p_{i}+\beta_{4}\times d_{i}$$ (26)

α∼N(0,1.5) (27)

β∼N(0,1.5) (28)

The response $y_{i}$ refers to an estimate for the main effect of contact from models m1, m4, and m5 for sensitivity analysis status (main analysis, Glottolog-area sensitivity analysis, and F3-based sensitivity analysis) and each dataset. α indicates the intercept. $\beta_{1}$ indicates the coefficient for each model *m* (with m1 representing the reference state), $\beta_{2}$ indicates the coefficient for each sensitivity status *s* (with the main model representing the reference state), $\beta_{3}$ indicates the parameter coefficient (with genetic contact representing the reference state, the other possibility is areal contact), and $\beta_{4}\;$indicates the dataset coefficient (with GBI representing the reference state, the other possibility is TLI).

#### Feature state meta-analysis model

$$y_{i}∼N\left(\mu_{i},\sigma_{i}\right)$$ (29)

$$\mu_{i}=\beta \times g_{i}$$ (30)

β∼N(0,1.5) (31)

The response $y_{i}$ refers to the effect of contact for each feature state. β indicates the coefficient for each level of *g*, where *g* is the categorical variable classifying states into domain groups (see section “Meta-analyses highlight differences between domains of language”). We fitted separate models for two separate classifications *g1* and *g2* for each of four types of contact (genetic contact, all pairs; genetic contact, different area pairs only; genetic contact, same area pairs only; same area).
