# Supplementary material for: Patterns of genetic admixture reveal similar rates of borrowing across diverse scenarios of language contact
Source: Sci Adv. 2025 Aug 29;11(35):eadv7521. doi: 10.1126/sciadv.adv7521 (PMC12396315; doi:10.1126/sciadv.adv7521)
Supplement: Supplementary file 1 — Figs. S1 to S32 Legends for tables S1 to S3, S24, and S25 Tables S4 to S23 [file sciadv.adv7521_sm.pdf]

Science Advances
AAAS

Supplementary Materials for

Patterns of genetic admixture reveal similar rates of borrowing across diverse scenarios of language contact

Anna Graff *et al.*

Corresponding author: Anna Graff, anna.graff@uzh.ch; Chiara Barbieri, barbieri.chiara@gmail.com

Sci. Adv. **11**, eadv7521 (2025)
DOI: 10.1126/sciadv.adv7521

The PDF file includes:

Figs. S1 to S32
Legends for tables S1 to S3, S24, and S25
Tables S4 to S23

Other Supplementary Material for this manuscript includes the following:

Tables S1 to S3, S24, and S25

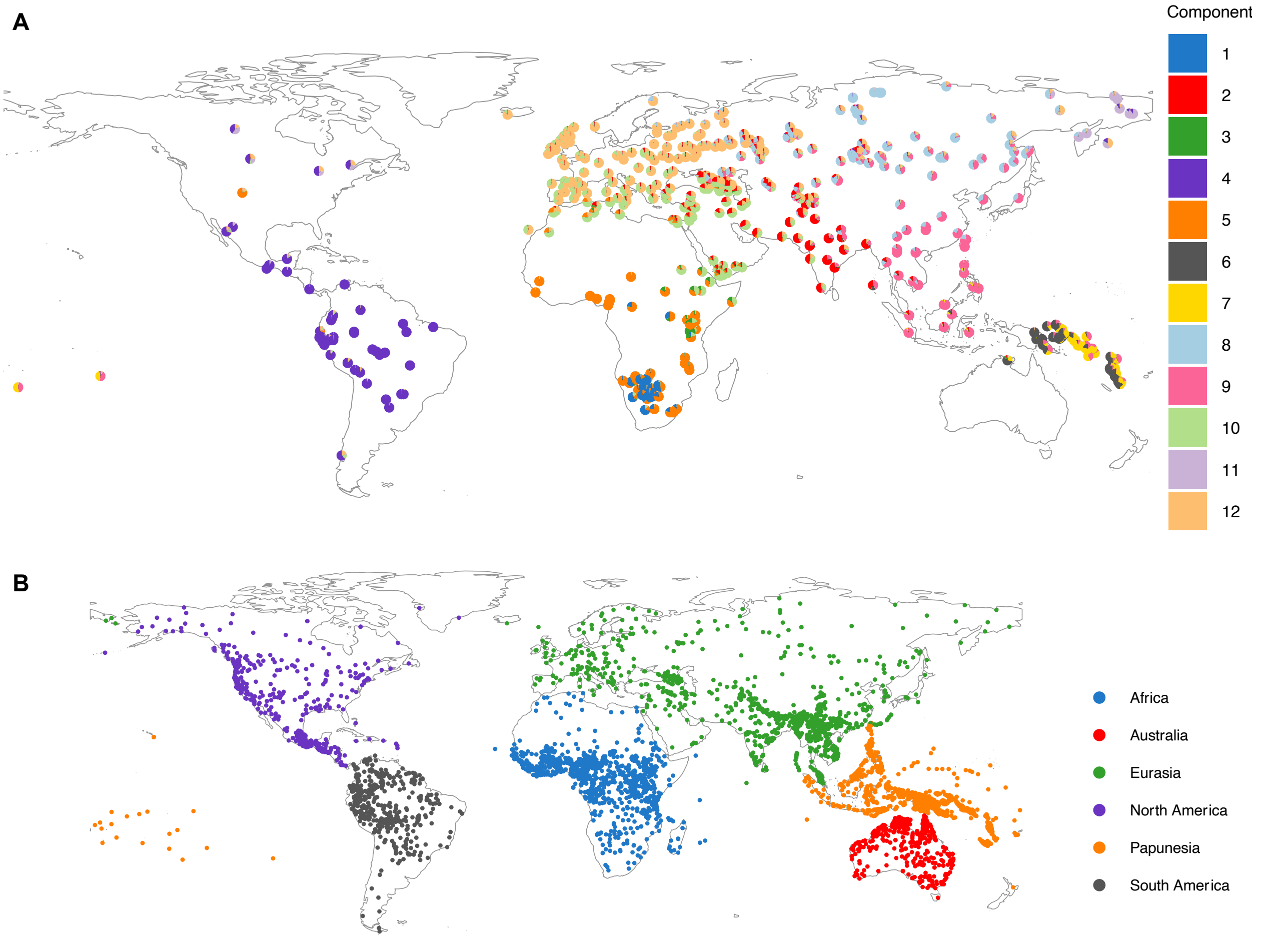

Fig. S1.
(**A**). Admixture pie chart for GeLaTo populations, assuming K = 12 ancestry components. (**B**). Language assignments to the six "macroareas" from Glottolog.

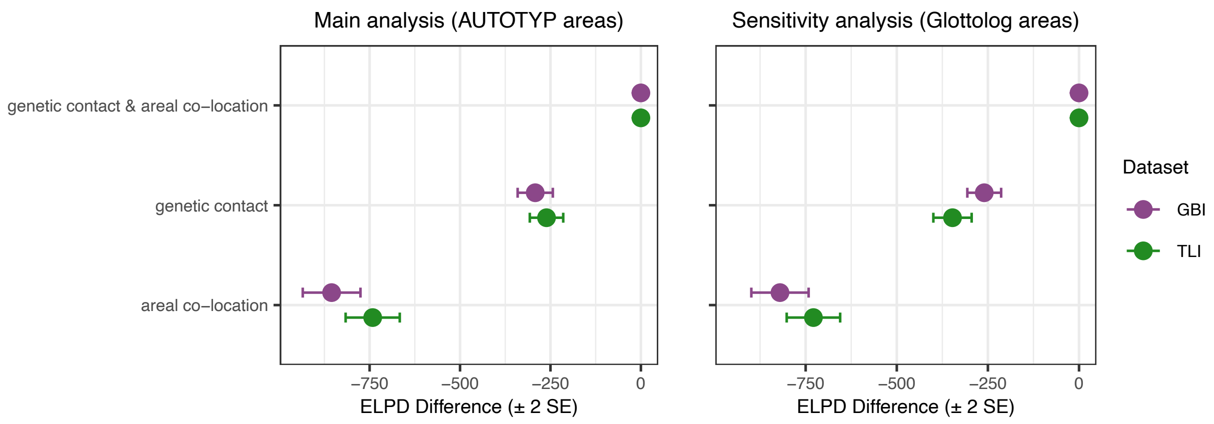

Fig. S2.

Model comparison using ELPD differences between the models using as fixed effects: both genetic contact and areal co-location (m1), only areal co-location (m2), only genetic contact (m3).

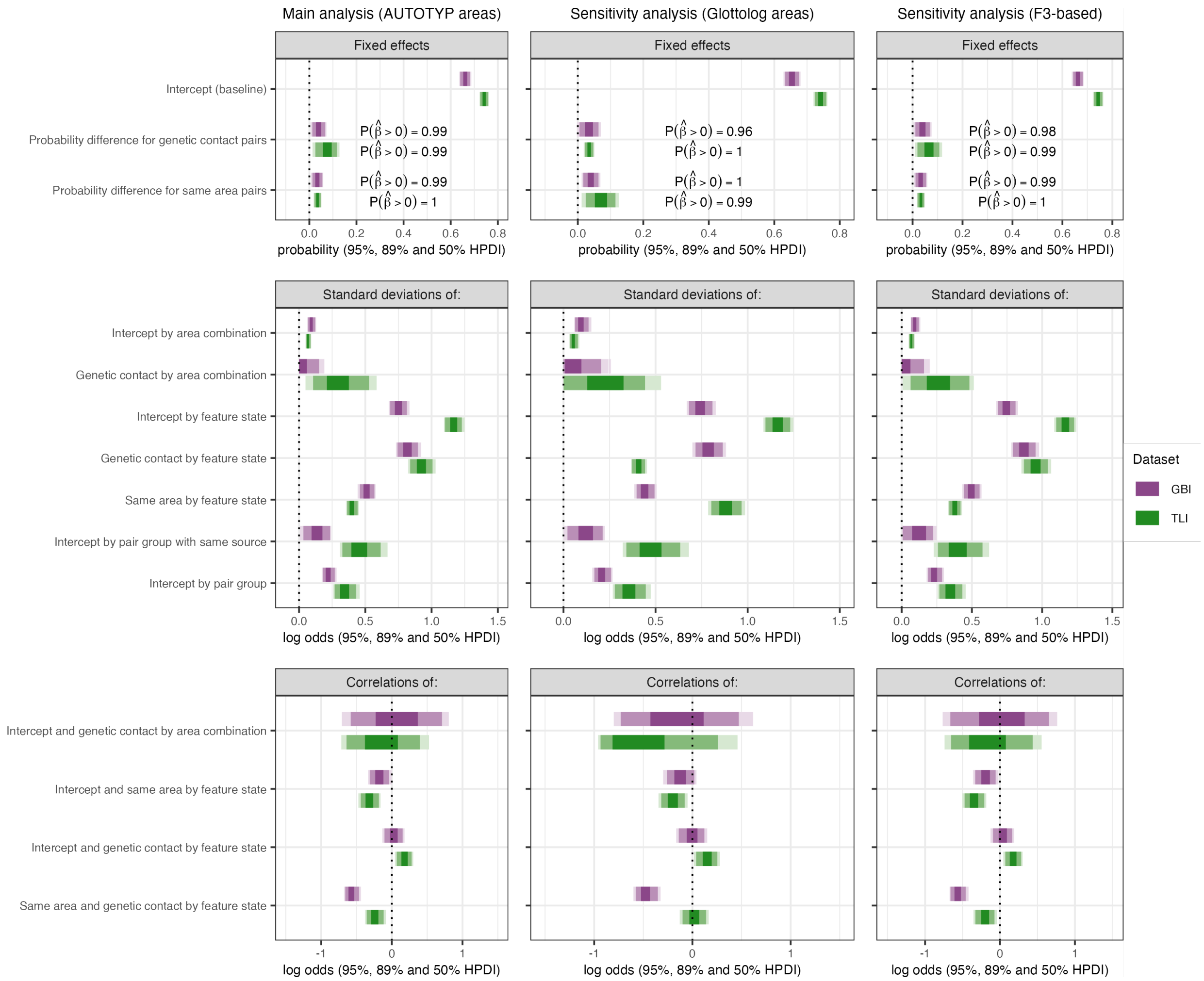

Fig. S3.

95%-, 89%- and 50%-HPDIs for model estimates of the combined models (m1) in the main and both sensitivity analyses.

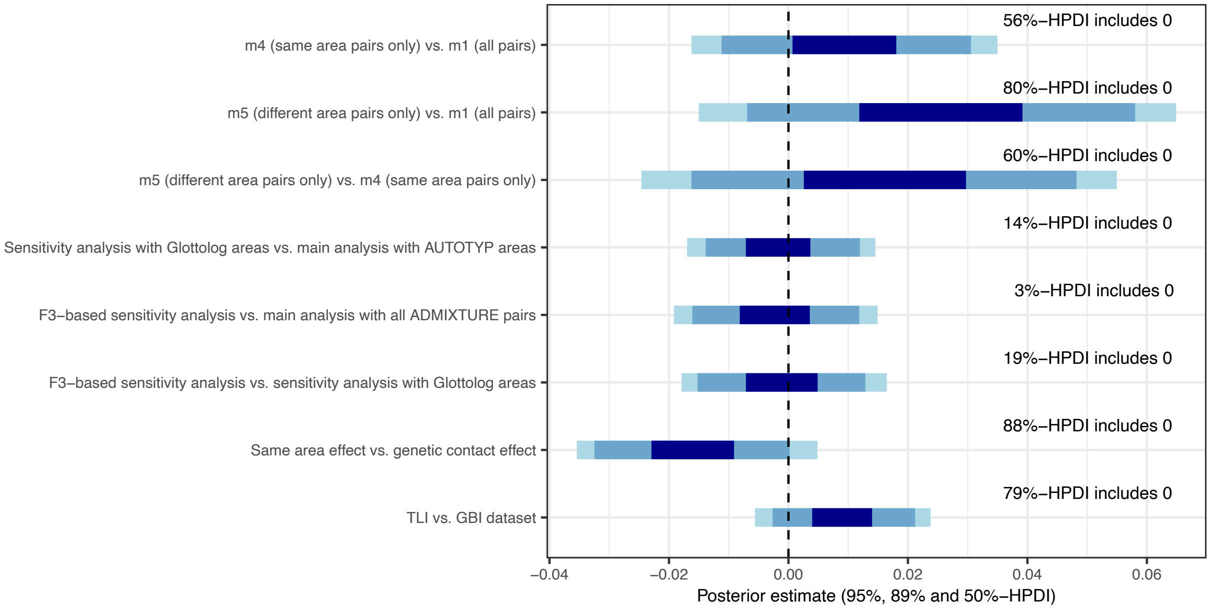

Fig. S4.

Posterior distributions of the effect estimates from the main effect robustness analysis. Each interval shows the 95%-, 89%- and 50%-HPDIs for model parameters. Annotations further indicate the narrowest HPDI that includes zero.

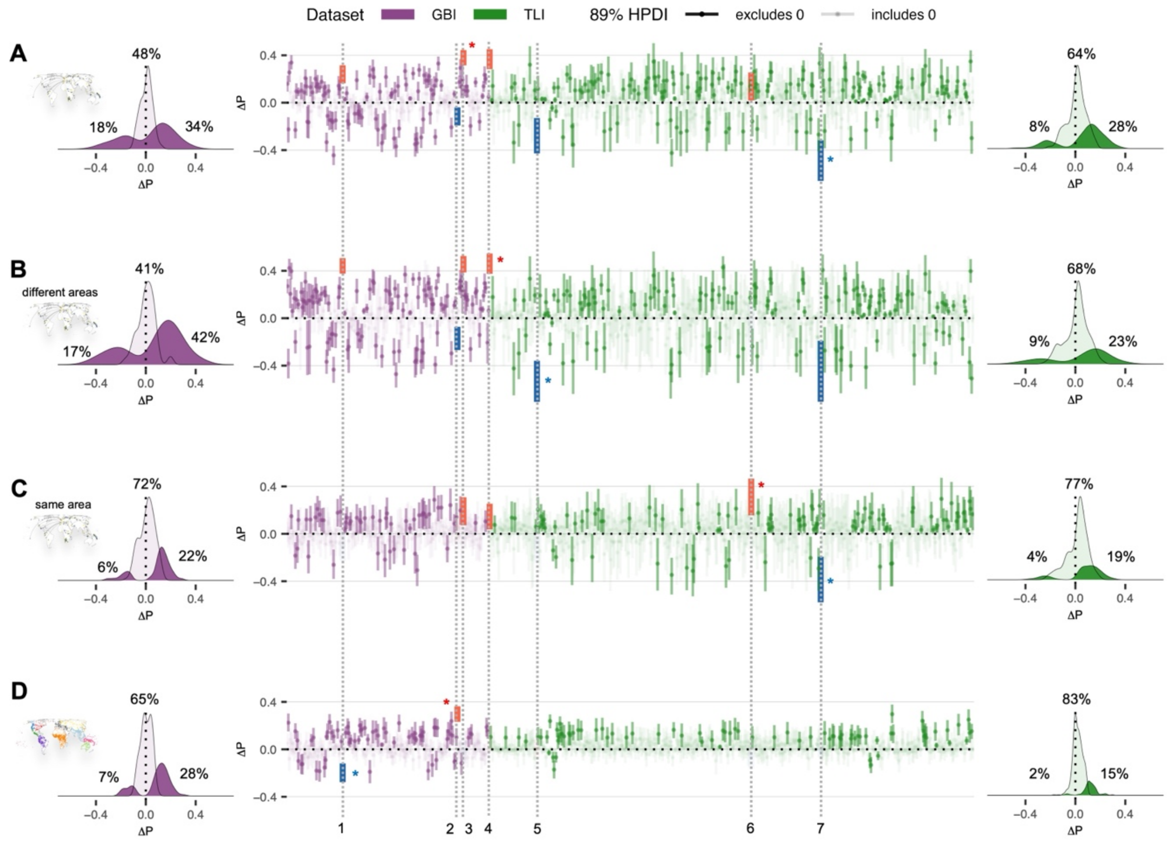

Fig. S5.

Effects of each contact type on the probability of language pairs sharing structural states in each dataset, using the AUTOTYP areas: (**A**) genetic contact (all pairs); (**B**) genetic contact (different area pairs only); (**C**) genetic contact (same area pairs only). (**D**) areal contact. Effects including zero in the 89% HPDI are plotted with transparent shading. Density plots show the mean contact effect on sharing across all features states, with percentage values indicating the proportion of states less likely, equally likely and more likely to be shared under each contact type compared to the relevant baseline. Interval plots show the effect intervals per state. The x-axis shows all 683 states, ordered alphabetically. The y-axis shows the difference in probability of sharing between contact vs. baseline pairs. Stars indicate the state with the highest positive (red) and highest negative (blue) effect in each panel. These states are then highlighted across the four contact types (panels A-D) and labelled (1-7). Grey lines allow the comparison of the effect of each highlighted state across contact conditions: States are coloured if they have a significant (excluding zero) diverging effect (in blue) or borrowing effect (in red). 1: GB126, existential verb. 2: GB623drmc, verbal prefixes and/or proclitics for speech act participants. 3: GB704drm, diminutive and/or augmentative marking on noun. 4: GB995F, noun-adjective and noun-demonstrative order. 5: TLI0560, concept of ‘eye’ colexified in the word for ‘tear’. 6: TLI0959: presence of distributive numerals. 7: TLI1017, word for distributive numerals.

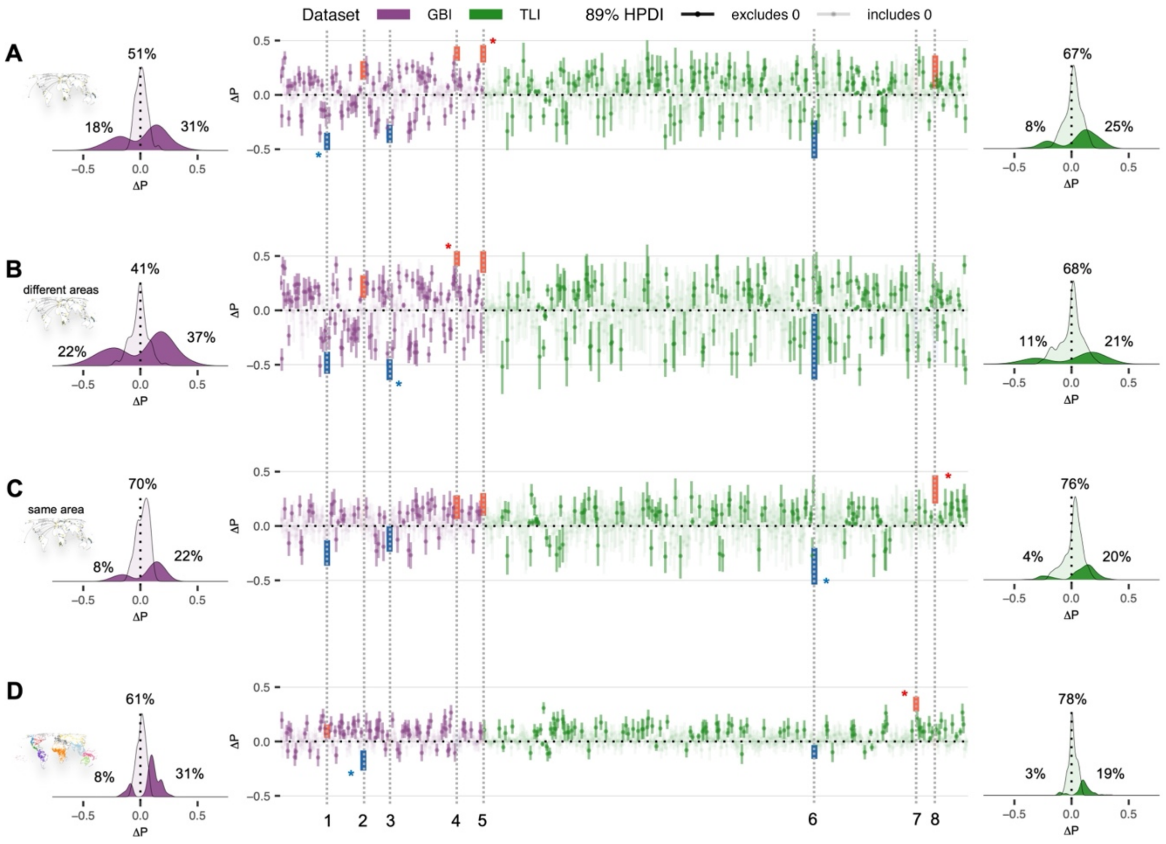

Fig. S6.

Effects of each contact type on the probability of language pairs sharing structural states in each dataset, using Glottolog areas. Same plotting conventions as in fig. S4: GB110cC, verb suppletion for tense or aspect (if morphological tense/aspect present). 2: GB196C, male-female distinction in 2p pronoun (if gender in 3p pronoun). 3: GB302c, phonologically free passive marker (if passive present). 4: GB704drm, diminutive and/or augmentative marking on noun. 5: GB995F, word order noun-adjective and noun-demonstrative. 6: TLI1017, word for distributive numerals. 7: TLI1084, indefinite pronouns related to generic nouns. 8: TLI1094, valency marking on fully inflected verb.

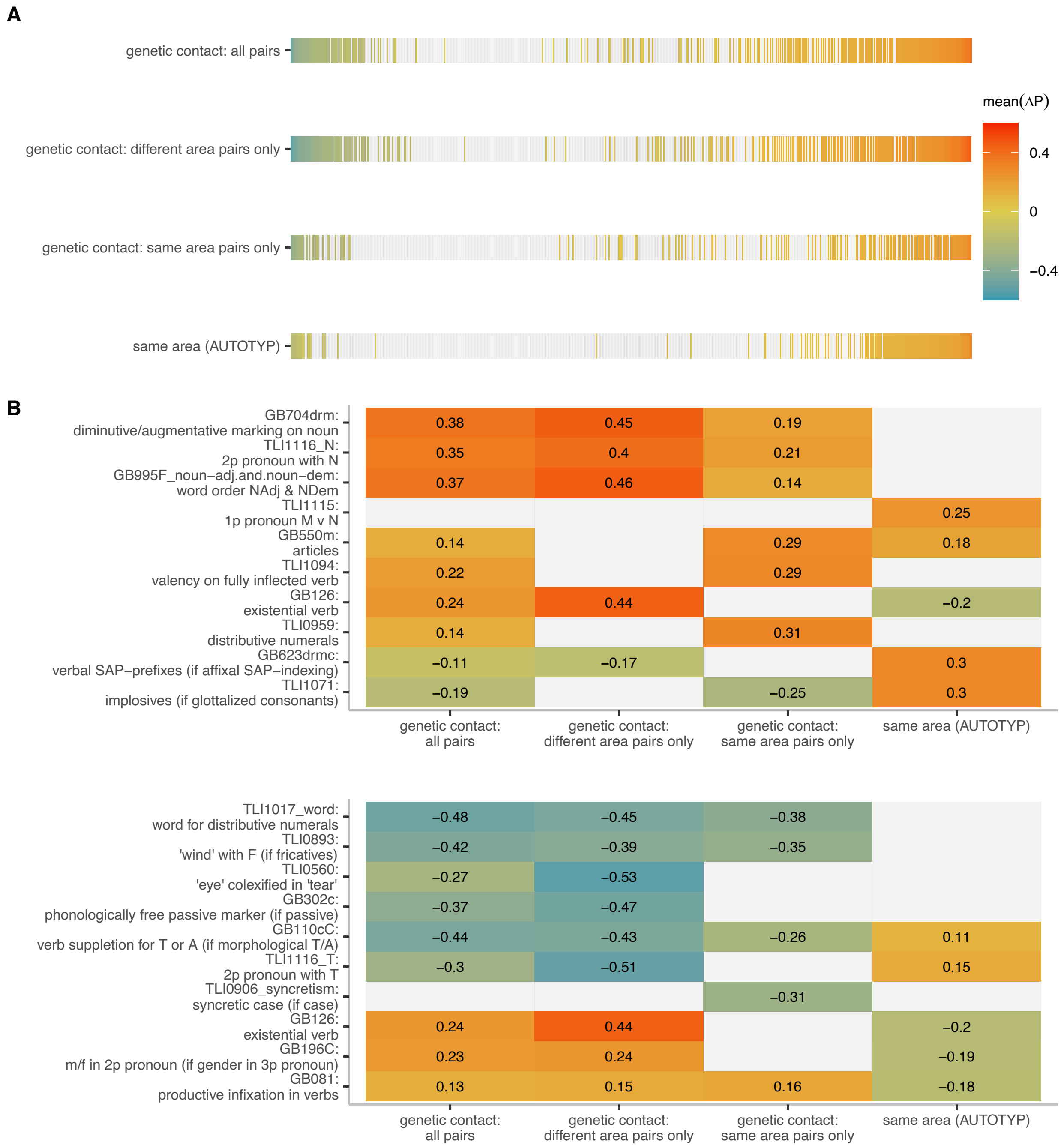

Fig. S7.

Alternative visualization of state sharing effect scales under each contact condition using AUTOTYP areas (genetic contact with all pairs; with different area pairs only; with same area pairs only; areal contact). Color indicates state sharing differences: shades of orange and red indicate convergence; shades of green and blue indicate divergence under contact. 89%-HPDIs including 0 are colored grey: for these, we cannot confidently declare a contact effect in any direction. (**A**) Individual heatmap scales of state sharing effects under each condition, each sorted in ascending magnitude of mean posterior effect. (**B**) Heatmaps of the three feature states with the strongest positive and negative (upper v. lower panel) effect on state sharing under each contact condition.

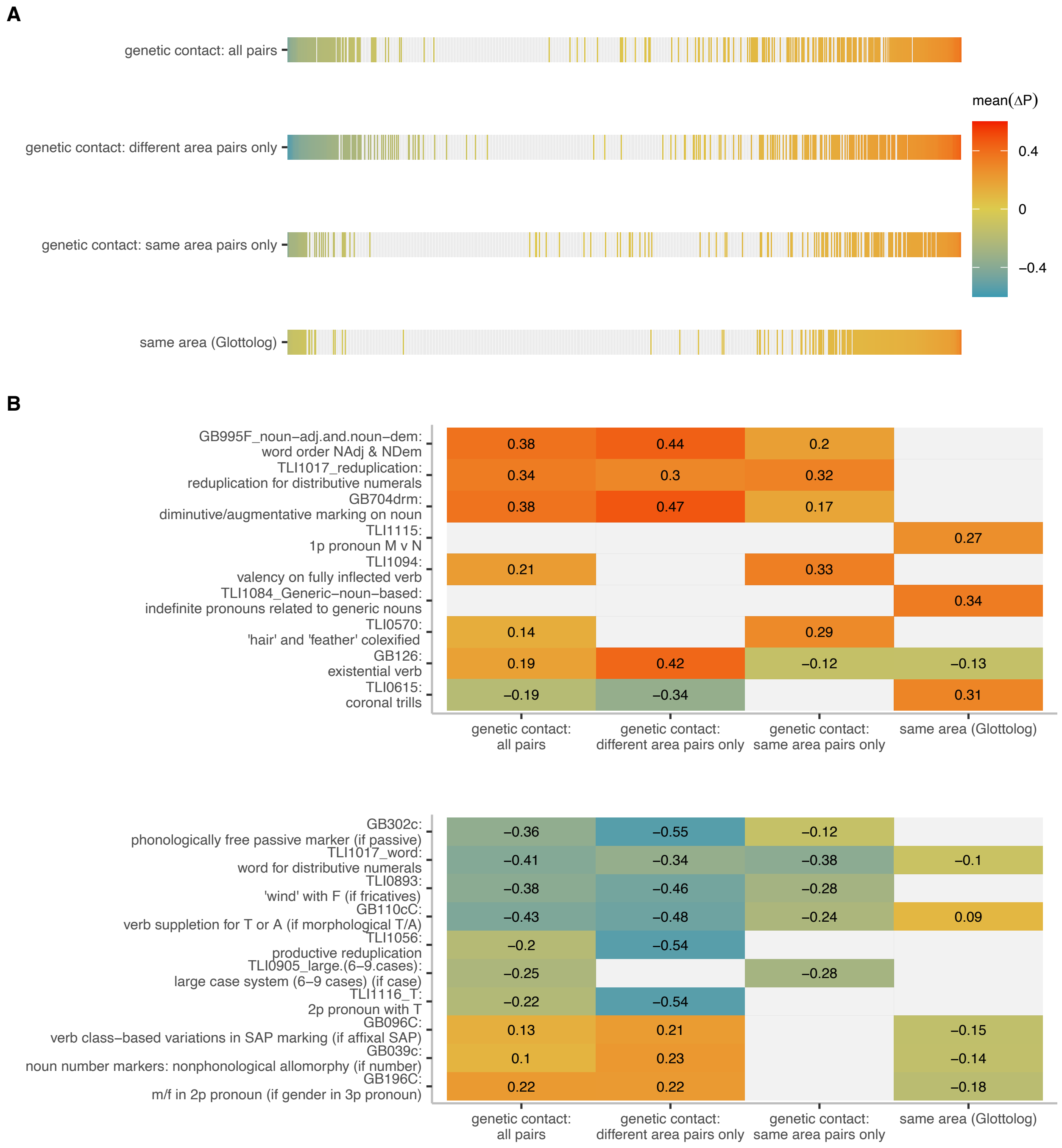

Fig. S8.

Alternative visualization of patterns of state sharing effect scales under each contact condition in the sensitivity analysis using the Glottolog areas. Same plotting conventions as in Fig. S7.

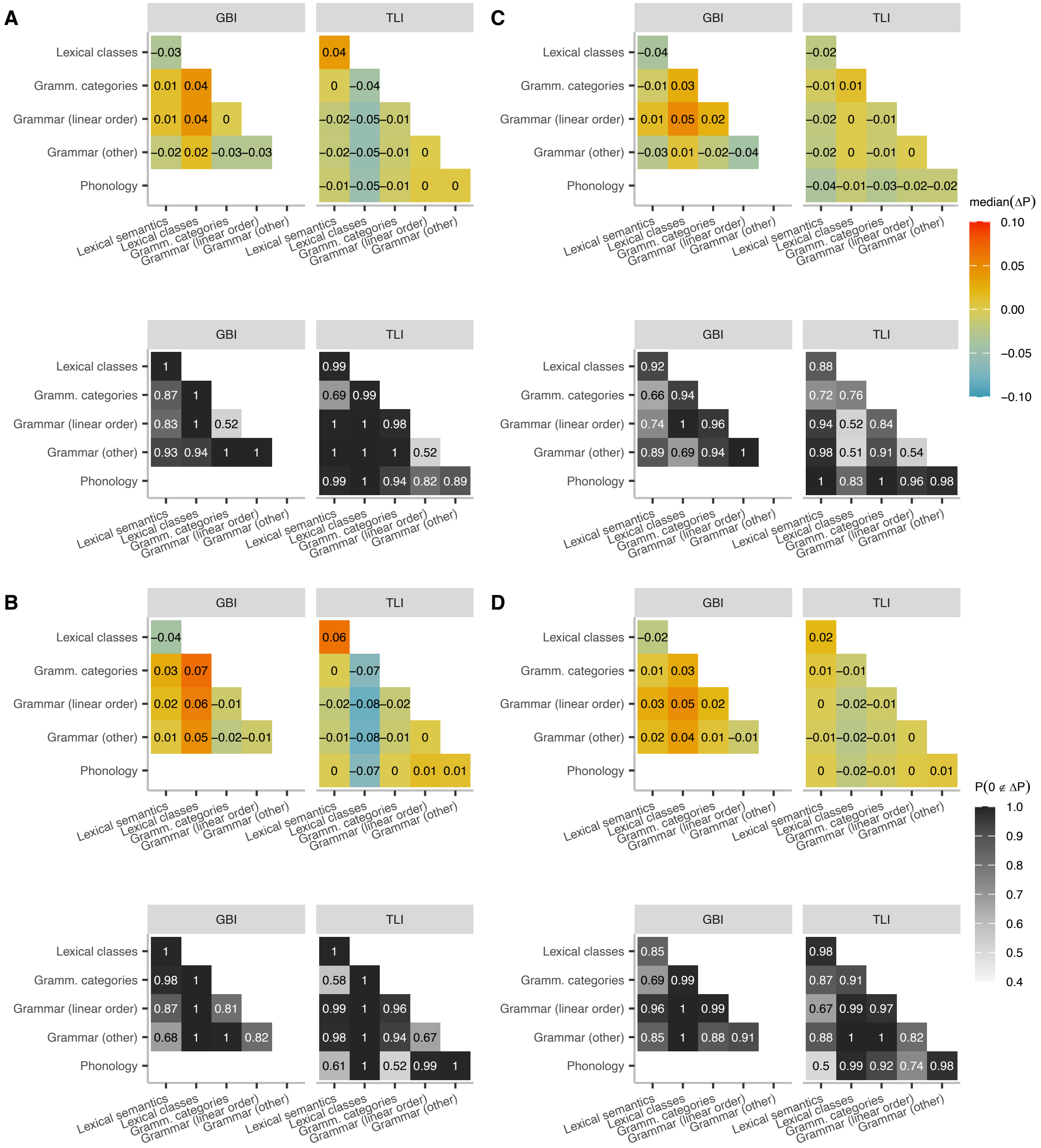

Fig. S9.

Meta-analysis of contact effects on features across domains of language under different types of contact, as an extension of Fig 4. The heatmap (upper plot) of each panel shows the posterior medians of borrowing probability differences between pairs of domains, irrespectively of the posterior's total distribution. The lower plot of each panel with a grey scale records the posterior probability of a non-zero difference. Contact types in subfigures are: (**A**) genetic contact, all pairs; (**B**) genetic contact: different area pairs only; (**C**) genetic contact: same area pairs only; (**D**) belonging to the same area (AUTOTYP). The GBI dataset does not include phonological data.

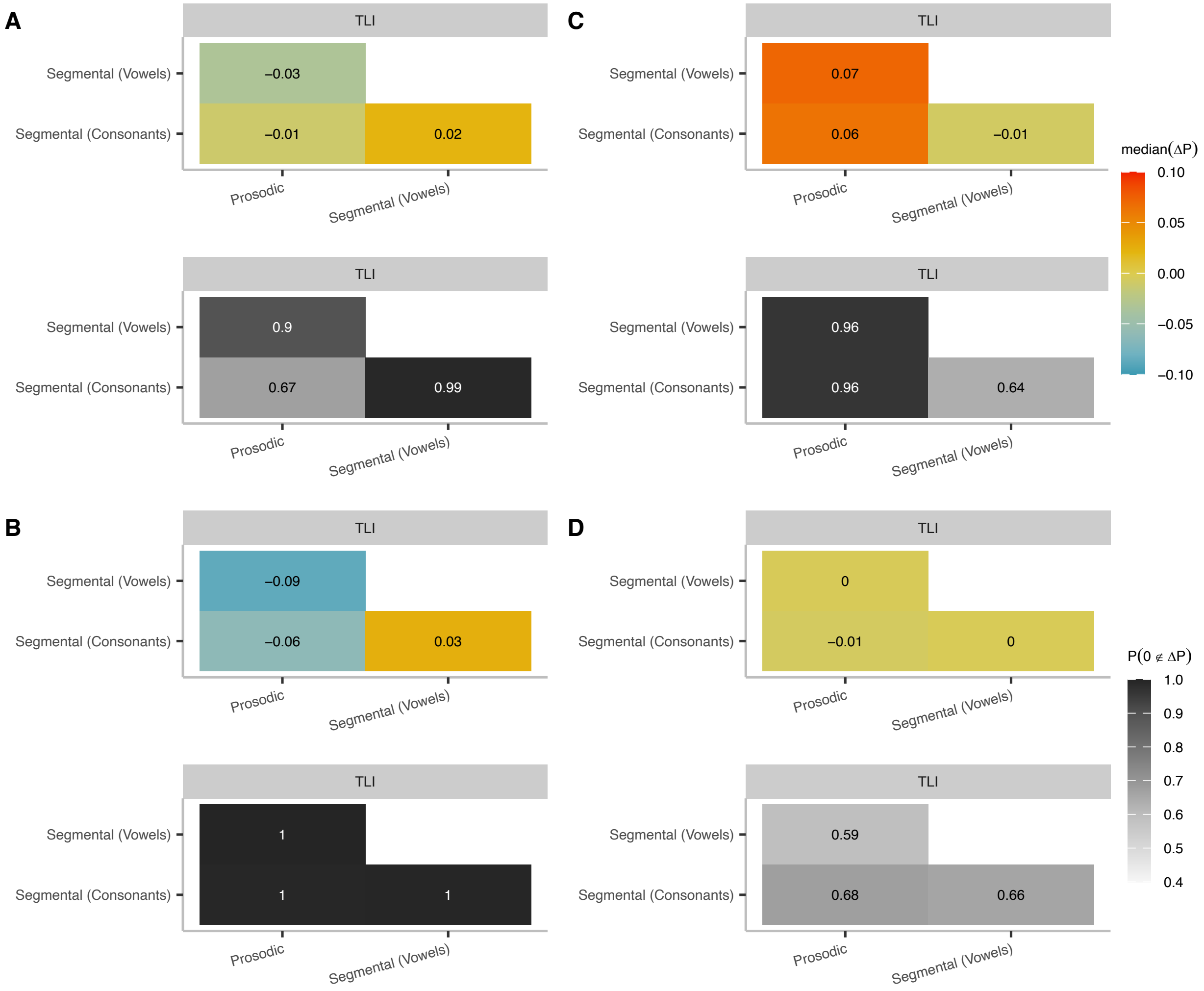

Fig. S10.

Meta-analysis of contact effects in phonology under different types of contact, extension of Fig 4. Same plotting conventions as Fig. S9.

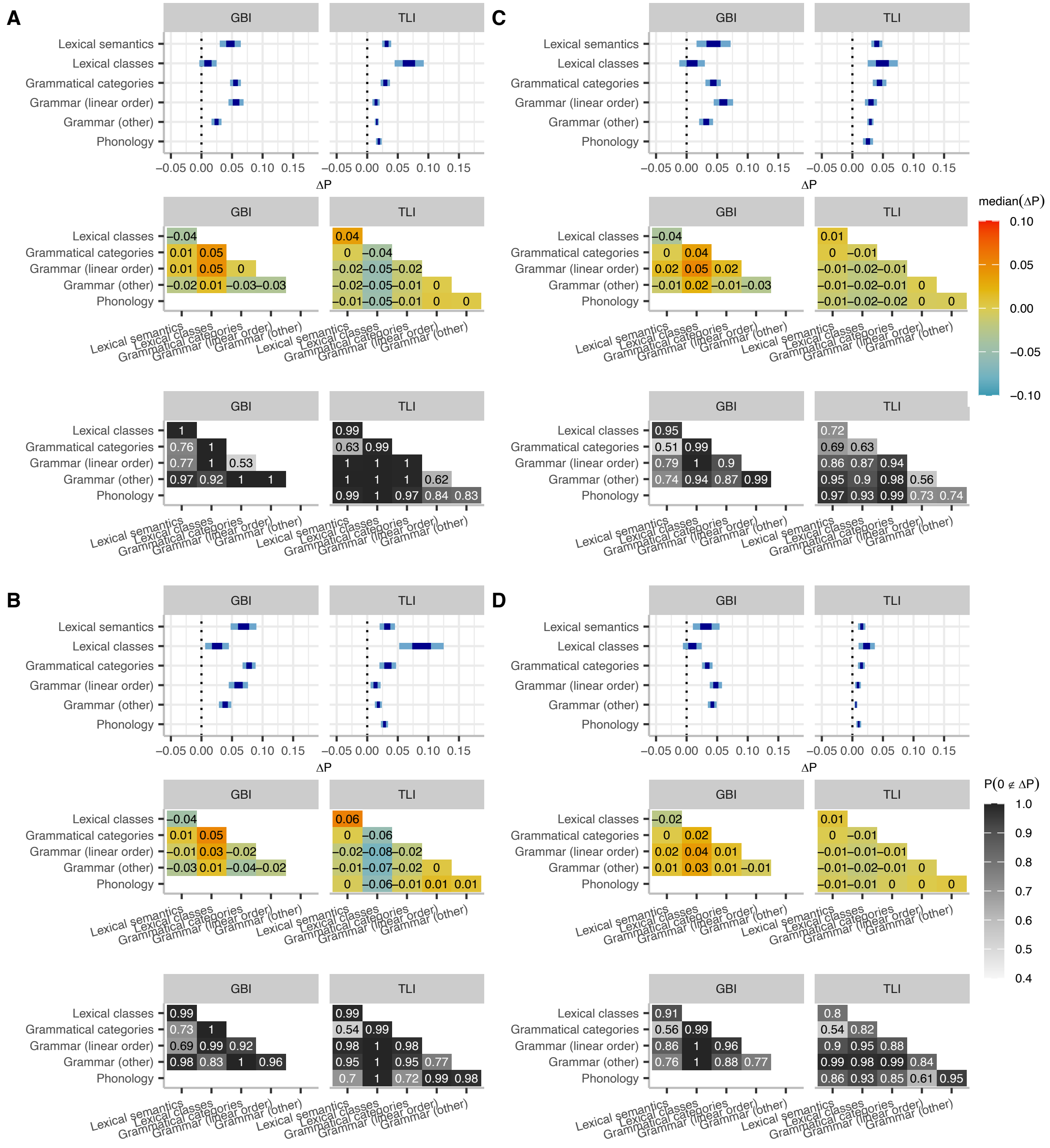

Fig. S11.

Meta-analysis of contact effects on features across domains under different types of contact, using the six Glottolog areas (sensitivity analysis). Same plotting conventions as Fig. S9.

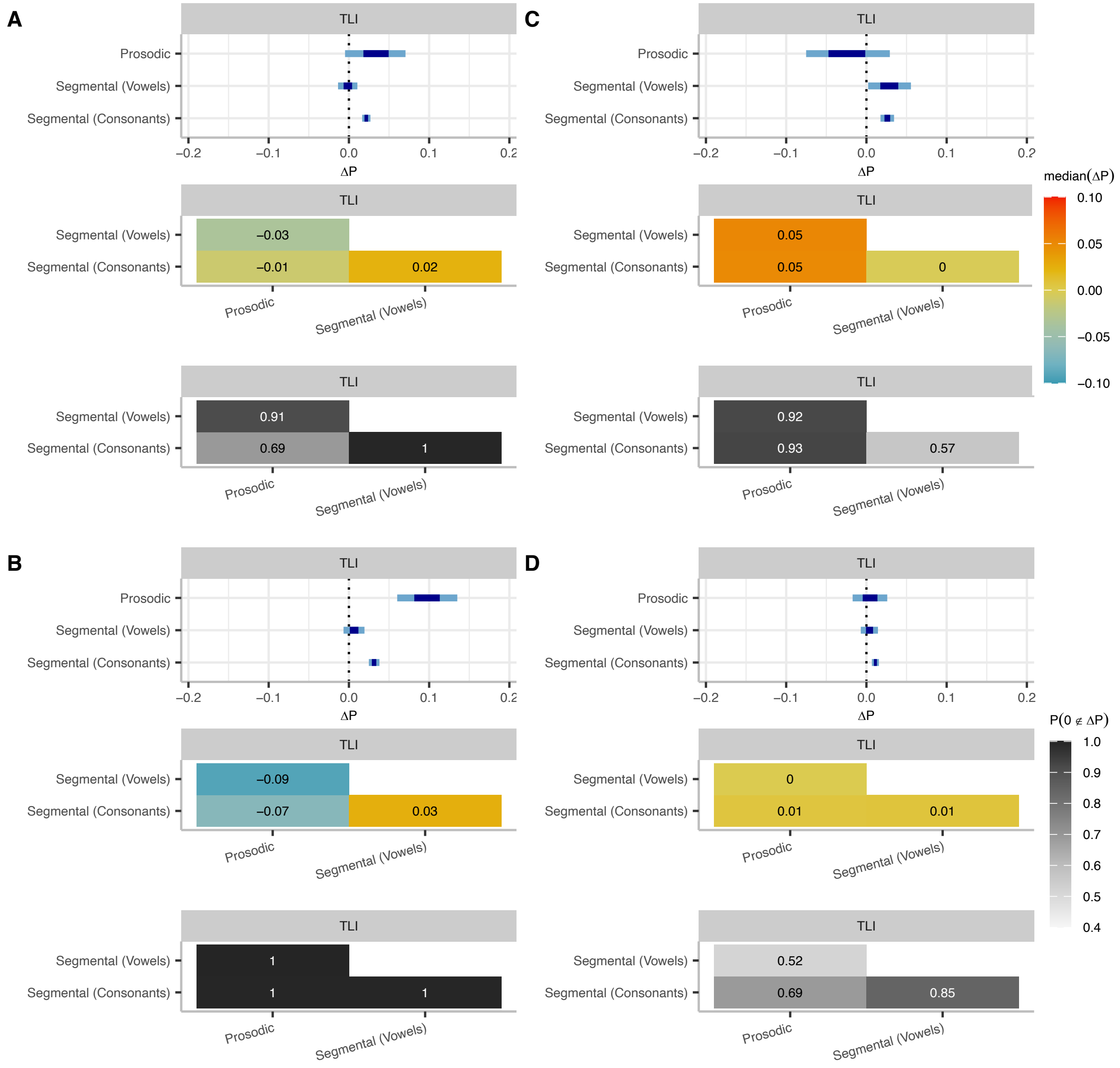

Fig. S12.

Meta-analysis of contact effects in phonology, using the six Glottolog areas (sensitivity analysis). Same plotting conventions as Fig. S9.

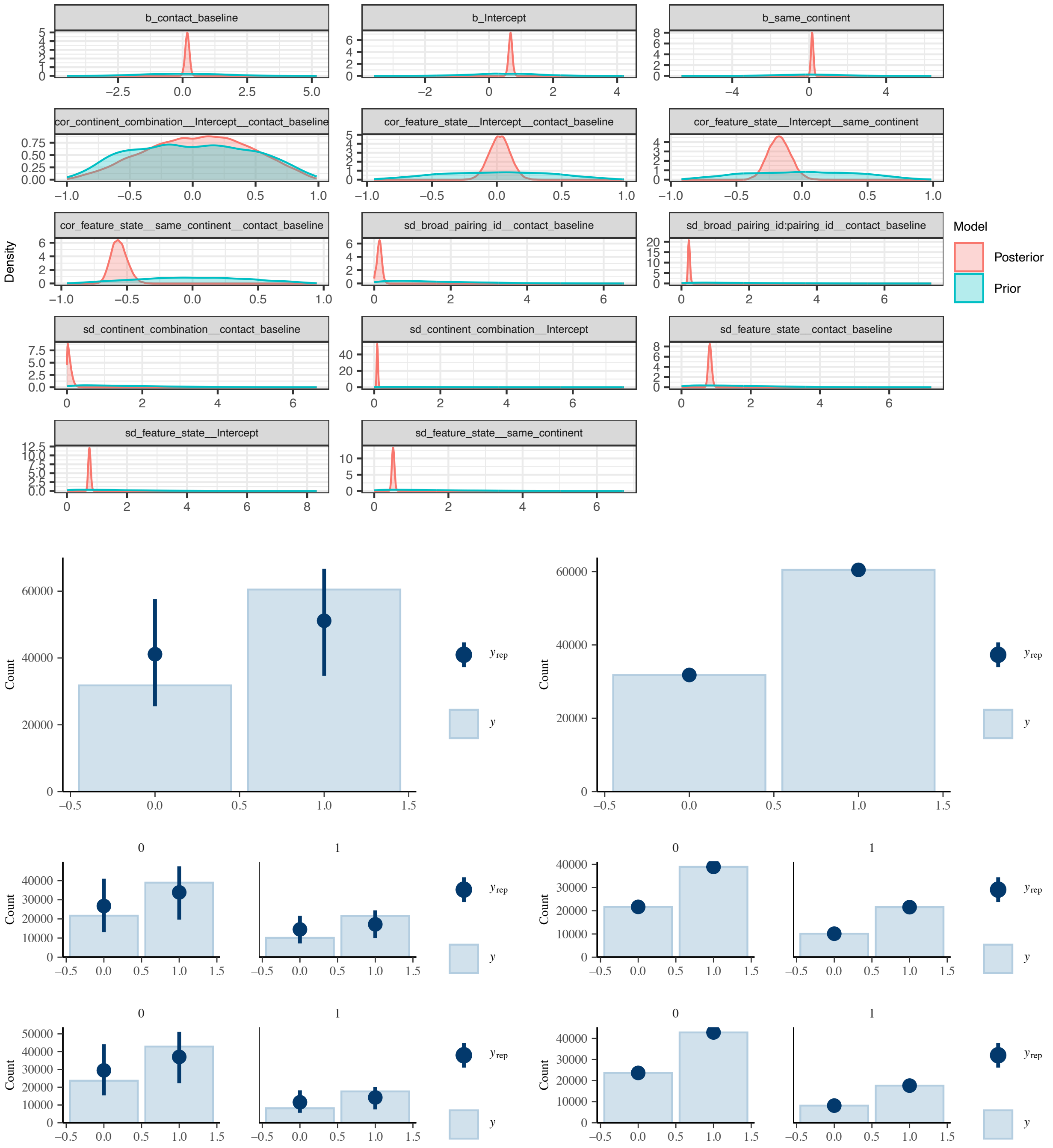

Fig. S13.

Prior and posterior distributions for regression coefficients and multilevel hyperparameters (top) as well as prior predictive checks (bottom left) and posterior predictive checks (bottom right) for regression coefficients: combined model (genetic contact and information on areal co-location), including all pairs, GBI data, main analysis (AUTOTYP areas).

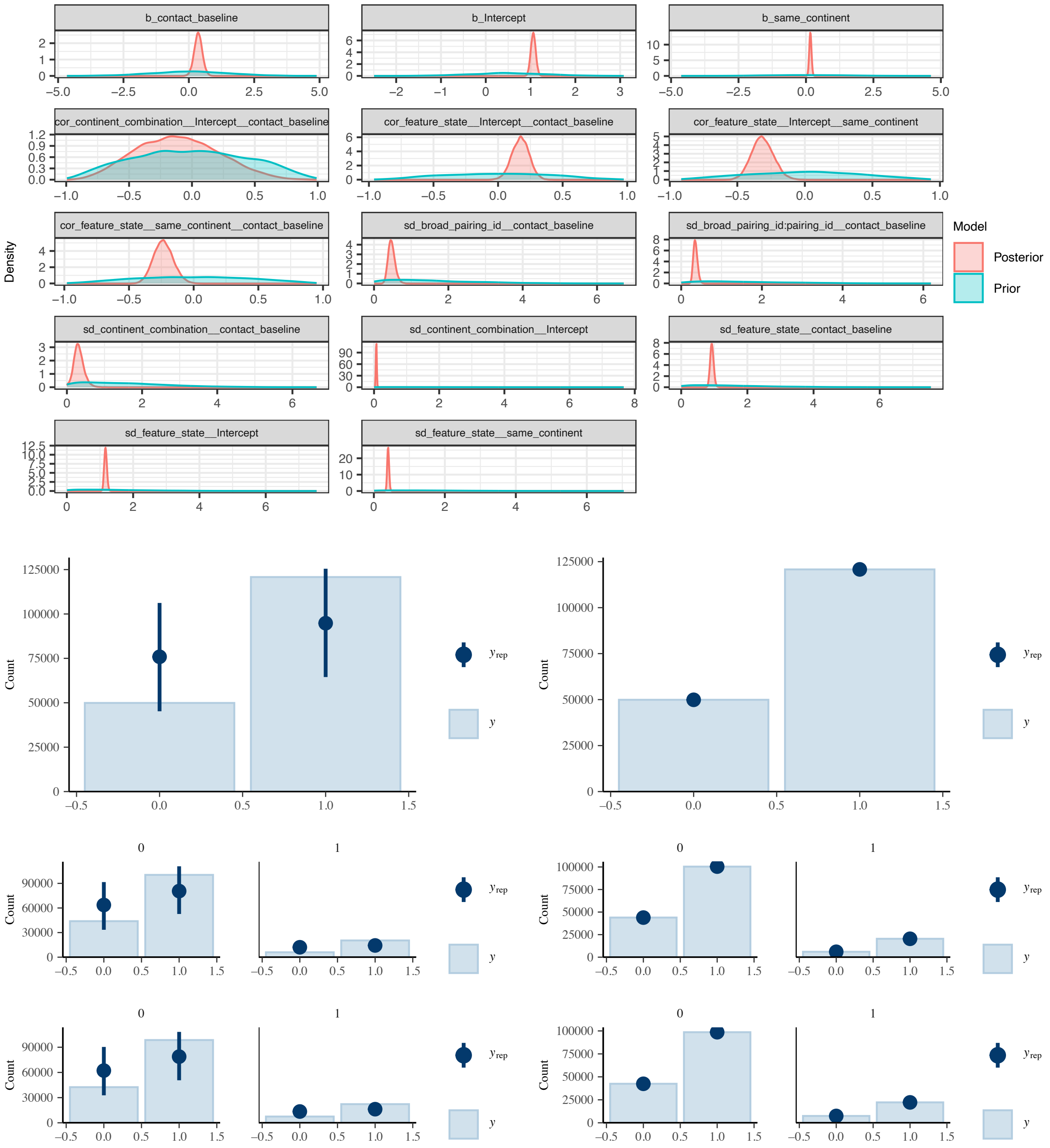

Fig. S14.

Prior and posterior distributions for regression coefficients and multilevel hyperparameters (top) as well as prior predictive checks (bottom left) and posterior predictive checks (bottom right) for regression coefficients: combined model (genetic contact and information on areal co-location), including all pairs, TLI data, main analysis (AUTOTYP areas).

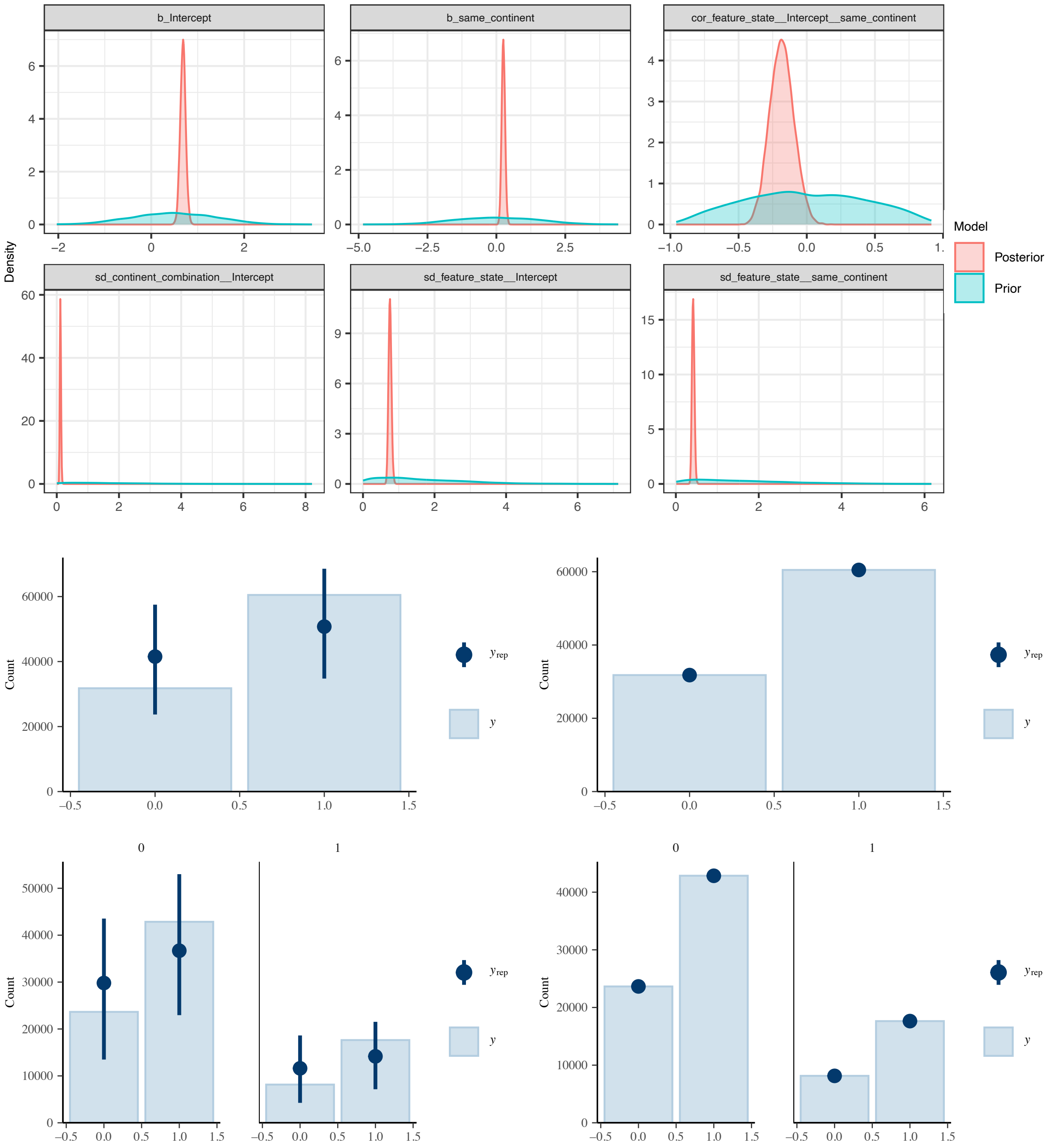

Fig. S15.

Prior and posterior distributions for regression coefficients and multilevel hyperparameters (top) as well as prior predictive checks (bottom left) and posterior predictive checks (bottom right) for regression coefficients: areal model, including all pairs, GBI data, main analysis (AUTOTYP areas).

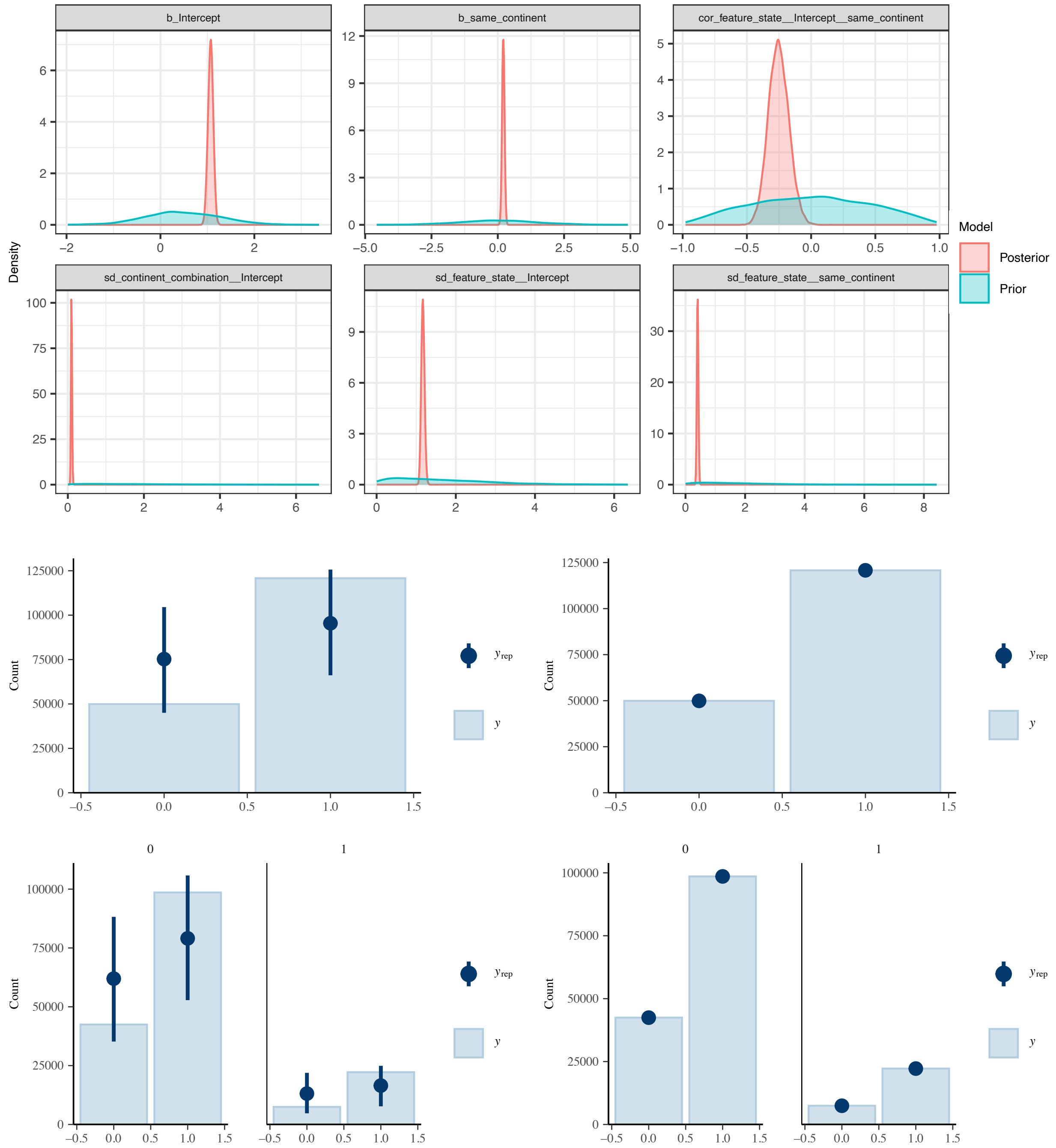

Fig. S16.

Prior and posterior distributions for regression coefficients and multilevel hyperparameters (top) as well as prior predictive checks (bottom left) and posterior predictive checks (bottom right) for regression coefficients: areal model, including all pairs, TLI data, main analysis (AUTOTYP areas).

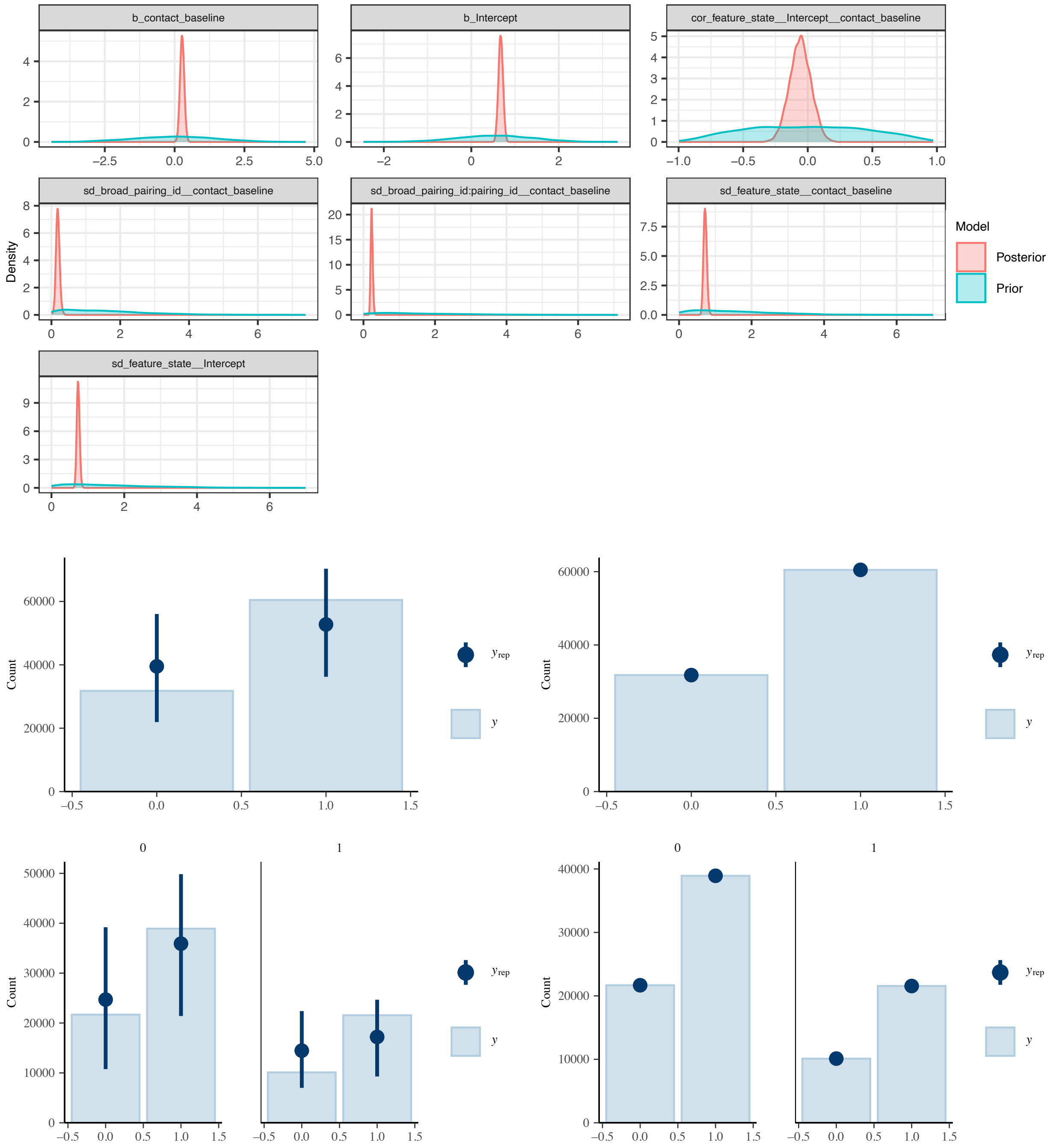

Fig. S17.

Prior and posterior distributions for regression coefficients and multilevel hyperparameters (top) as well as prior predictive checks (bottom left) and posterior predictive checks (bottom right) for regression coefficients: genetic model, including all pairs, GBI data.

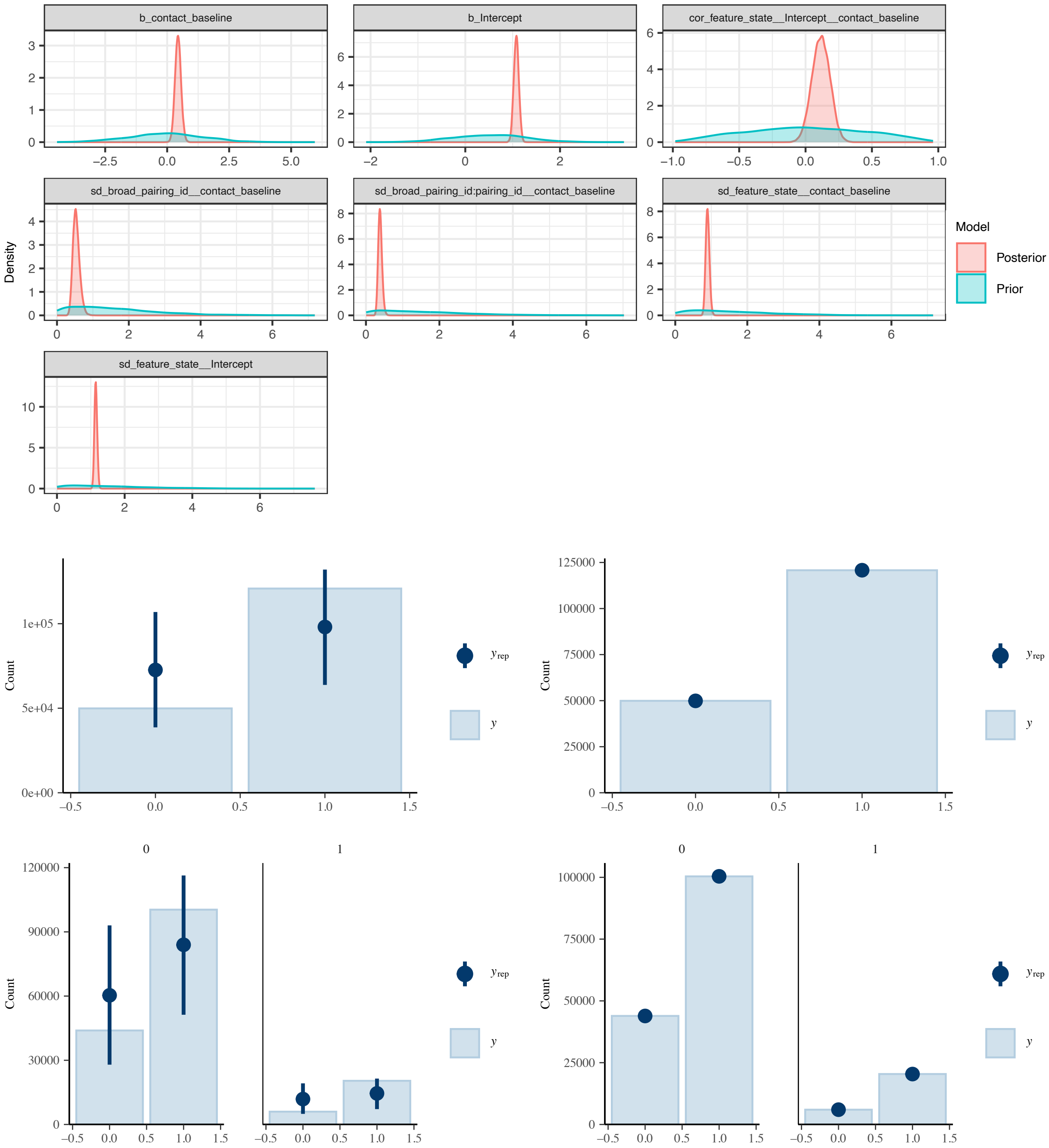

Fig. S18.

Prior and posterior distributions for regression coefficients and multilevel hyperparameters (top) as well as prior predictive checks (bottom left) and posterior predictive checks (bottom right) for regression coefficients: genetic model, including all pairs, TLI data.

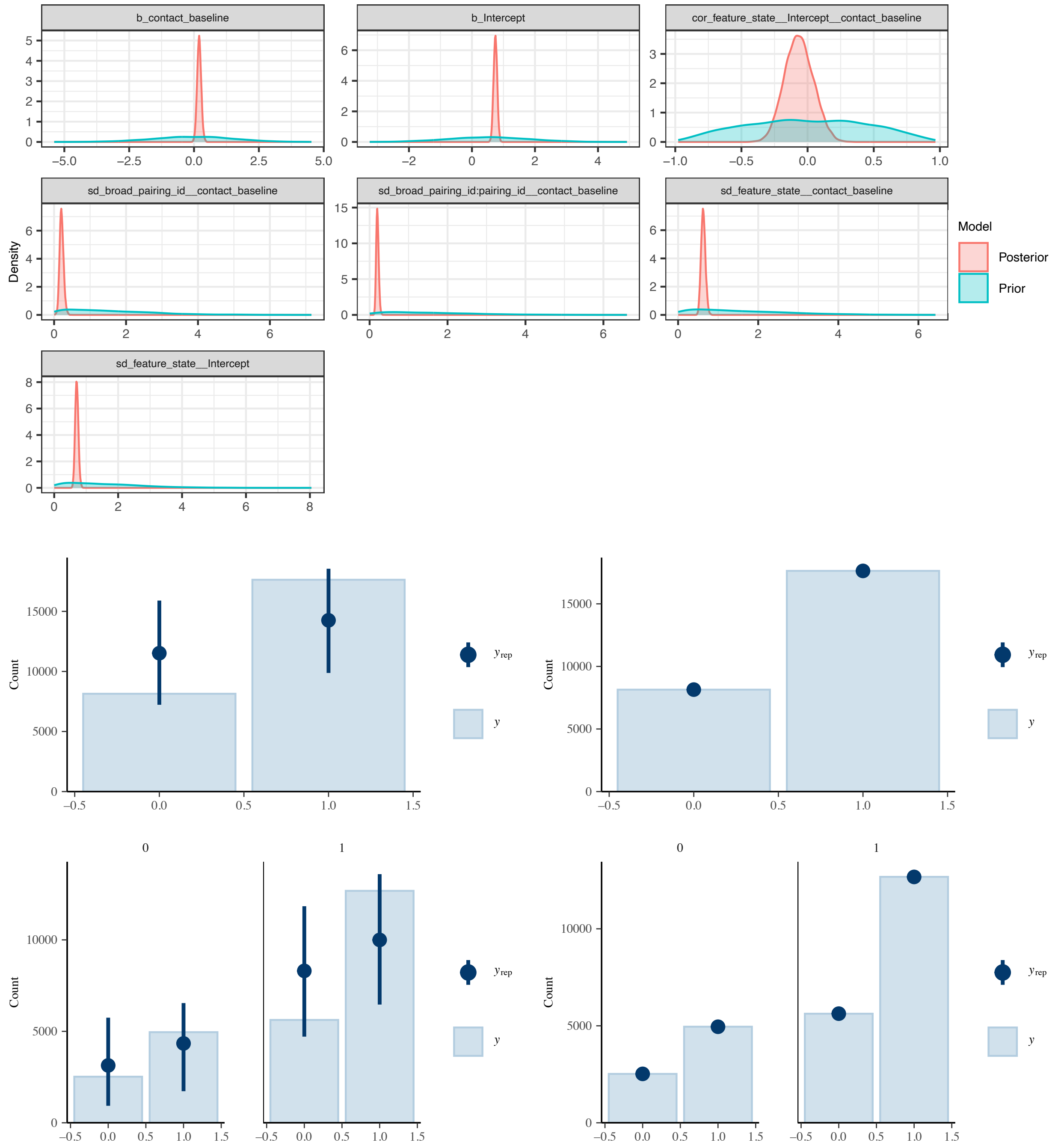

Fig. S19.

Prior and posterior distributions for regression coefficients and multilevel hyperparameters (top) as well as prior predictive checks (bottom left) and posterior predictive checks (bottom right) for regression coefficients: genetic model, including only pairs from the same area, GBI data, main analysis (AUTOTYP areas).

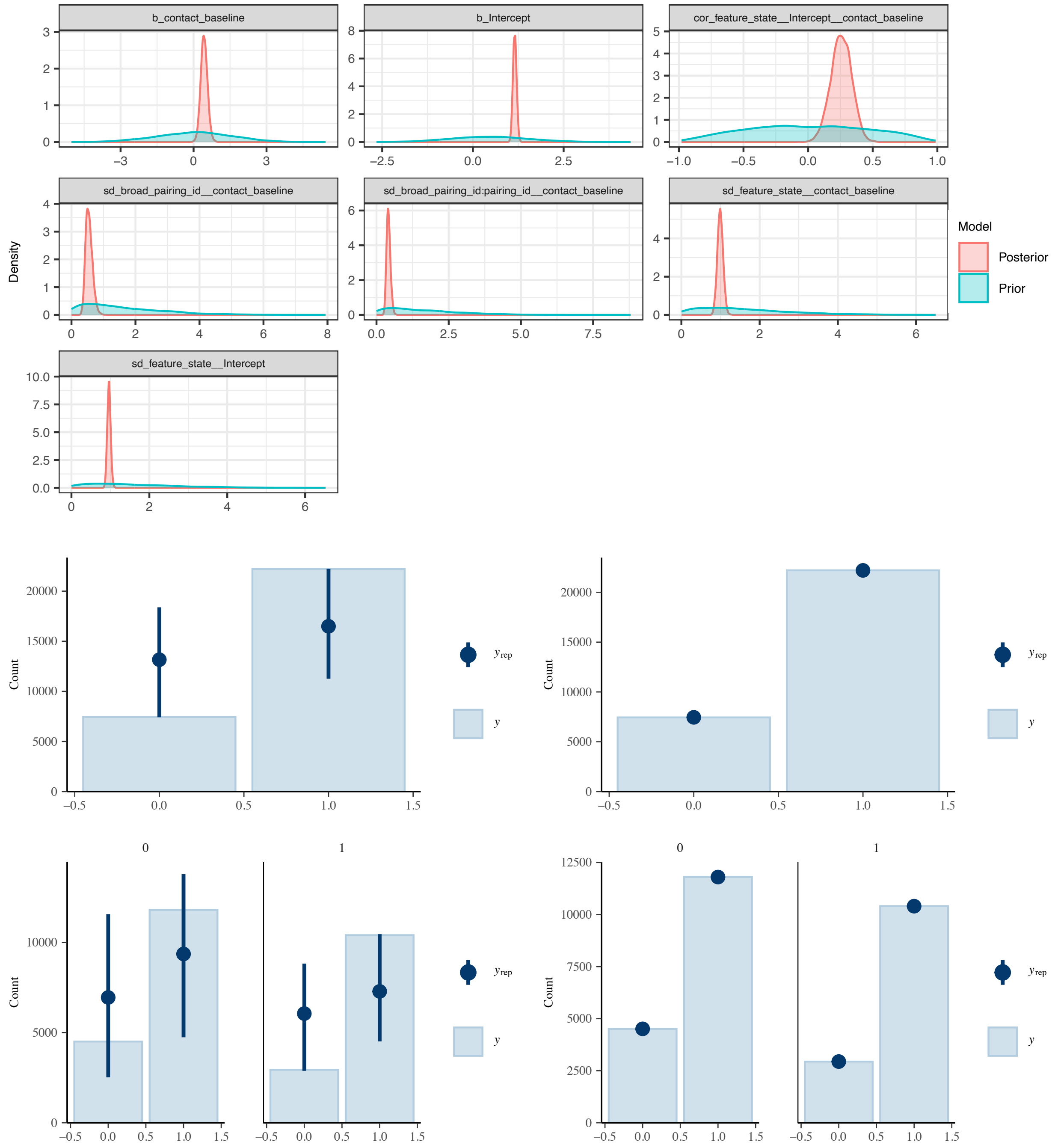

Fig. S20.

Prior and posterior distributions for regression coefficients and multilevel hyperparameters (top) as well as prior predictive checks (bottom left) and posterior predictive checks (bottom right) for regression coefficients: genetic model, including only pairs from the same area, TLI data, main analysis (AUTOTYP areas).

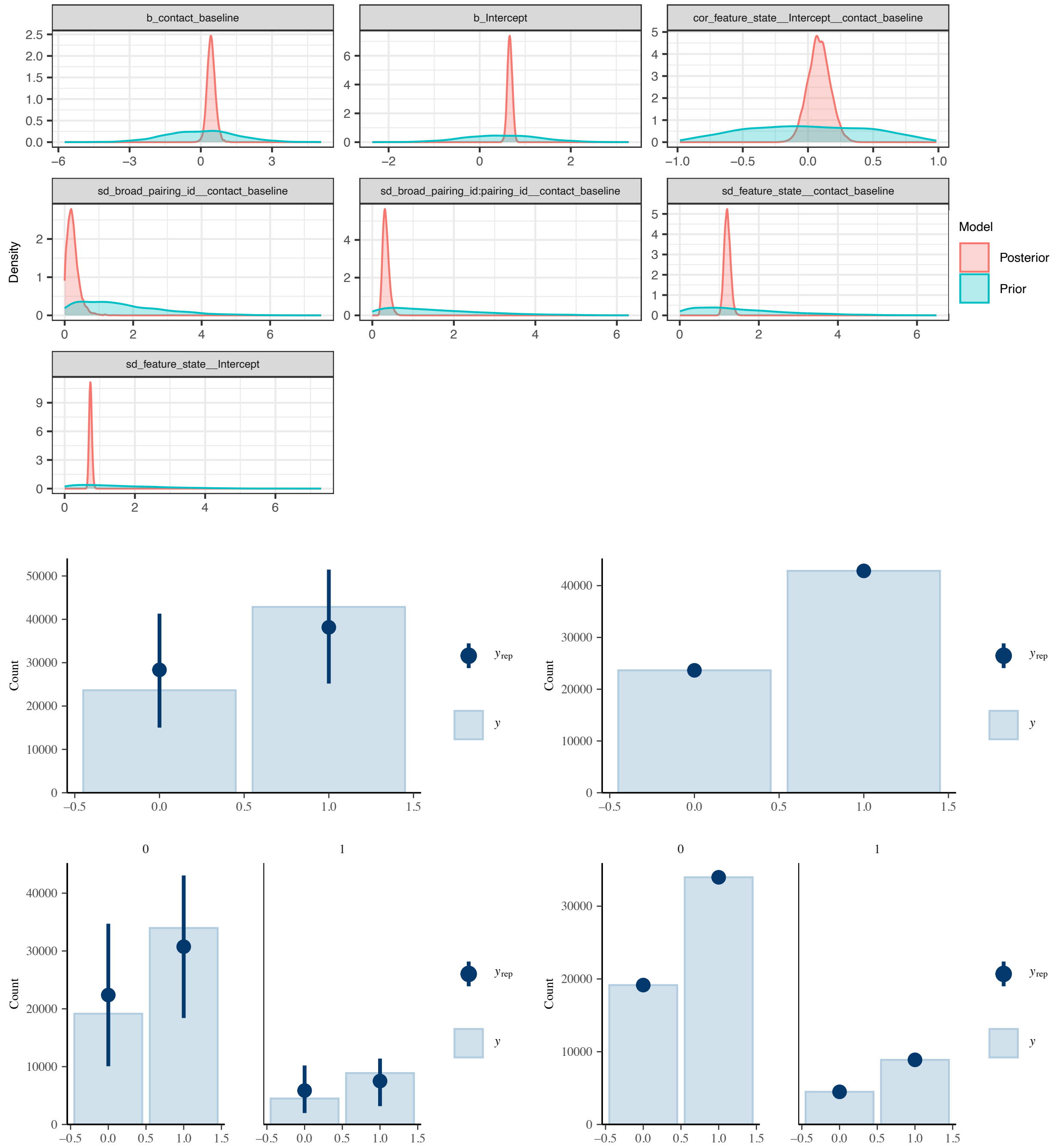

Fig. S21.

Prior and posterior distributions for regression coefficients and multilevel hyperparameters (top) as well as prior predictive checks (bottom left) and posterior predictive checks (bottom right) for regression coefficients: genetic model, including only pairs from different areas, GBI data, main analysis (AUTOTYP areas).

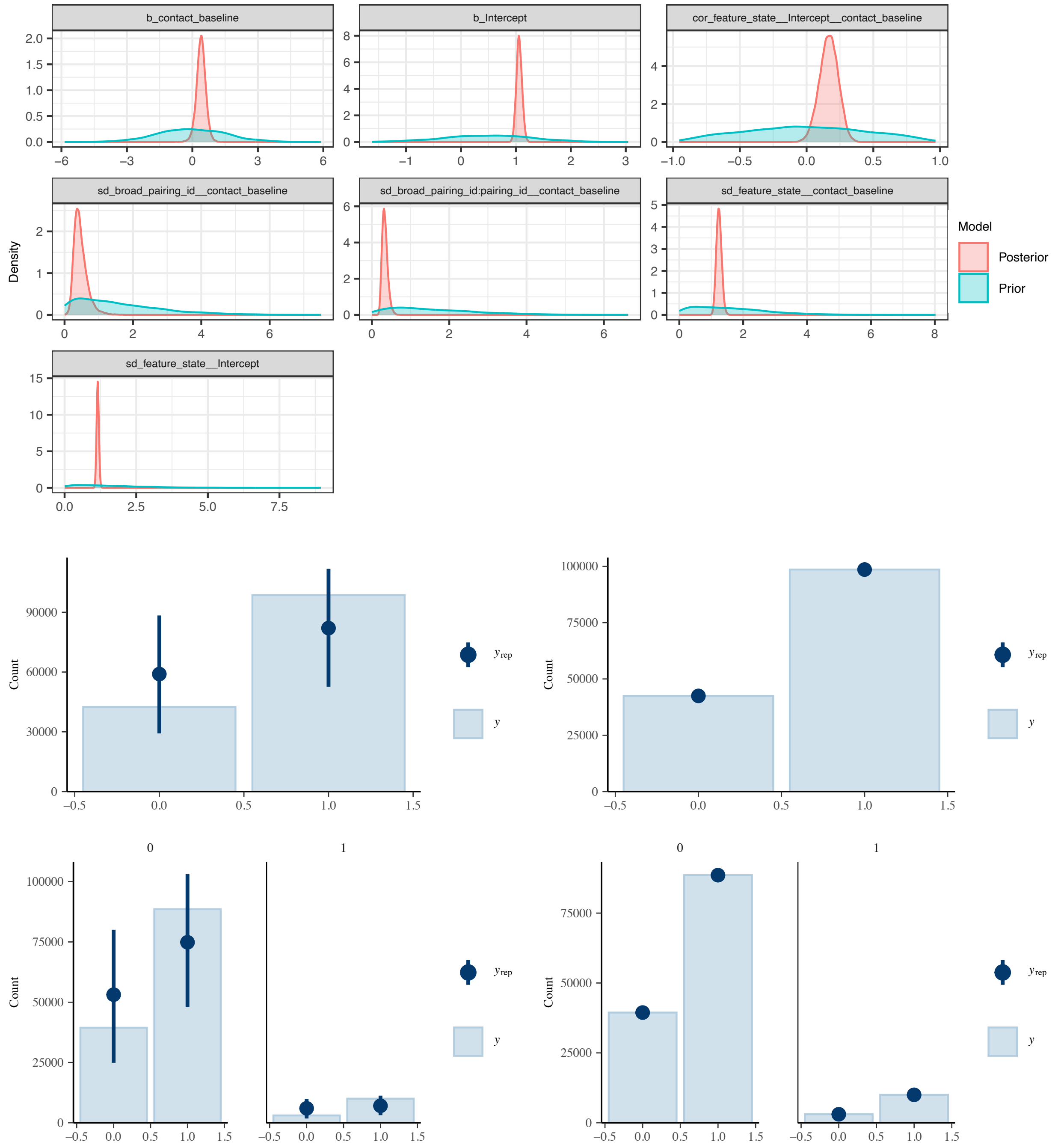

Fig. S22.

Prior and posterior distributions for regression coefficients and multilevel hyperparameters (top) as well as prior predictive checks (bottom left) and posterior predictive checks (bottom right) for regression coefficients: genetic model, including only pairs from different areas, TLI data, main analysis (AUTOTYP areas).

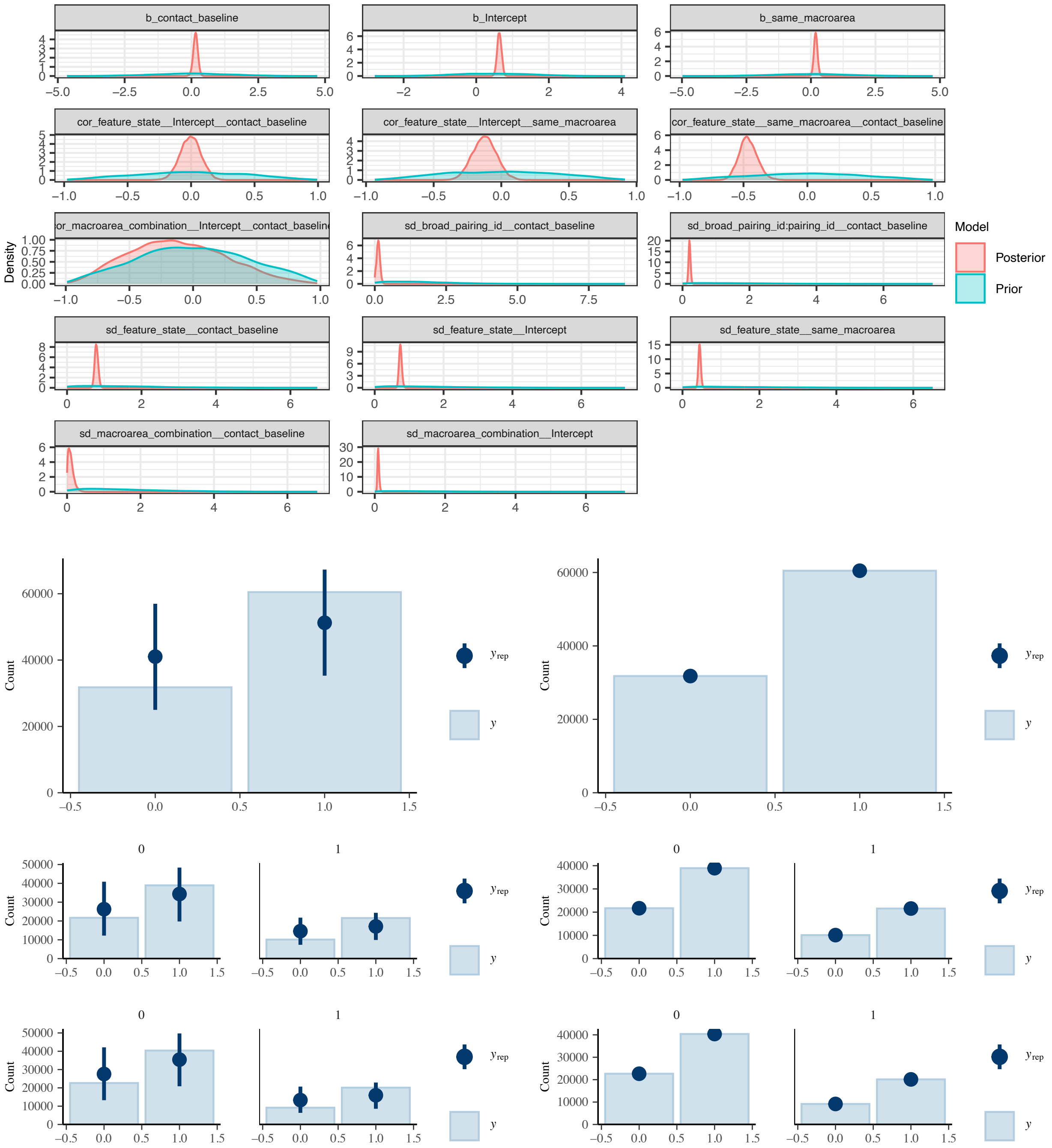

Fig. S23.

Prior and posterior distributions for regression coefficients and multilevel hyperparameters (top) as well as prior predictive checks (bottom left) and posterior predictive checks (bottom right) for regression coefficients: combined model (genetic contact and information on areal co-location), including all pairs, GBI data, sensitivity analysis (Glottolog areas).

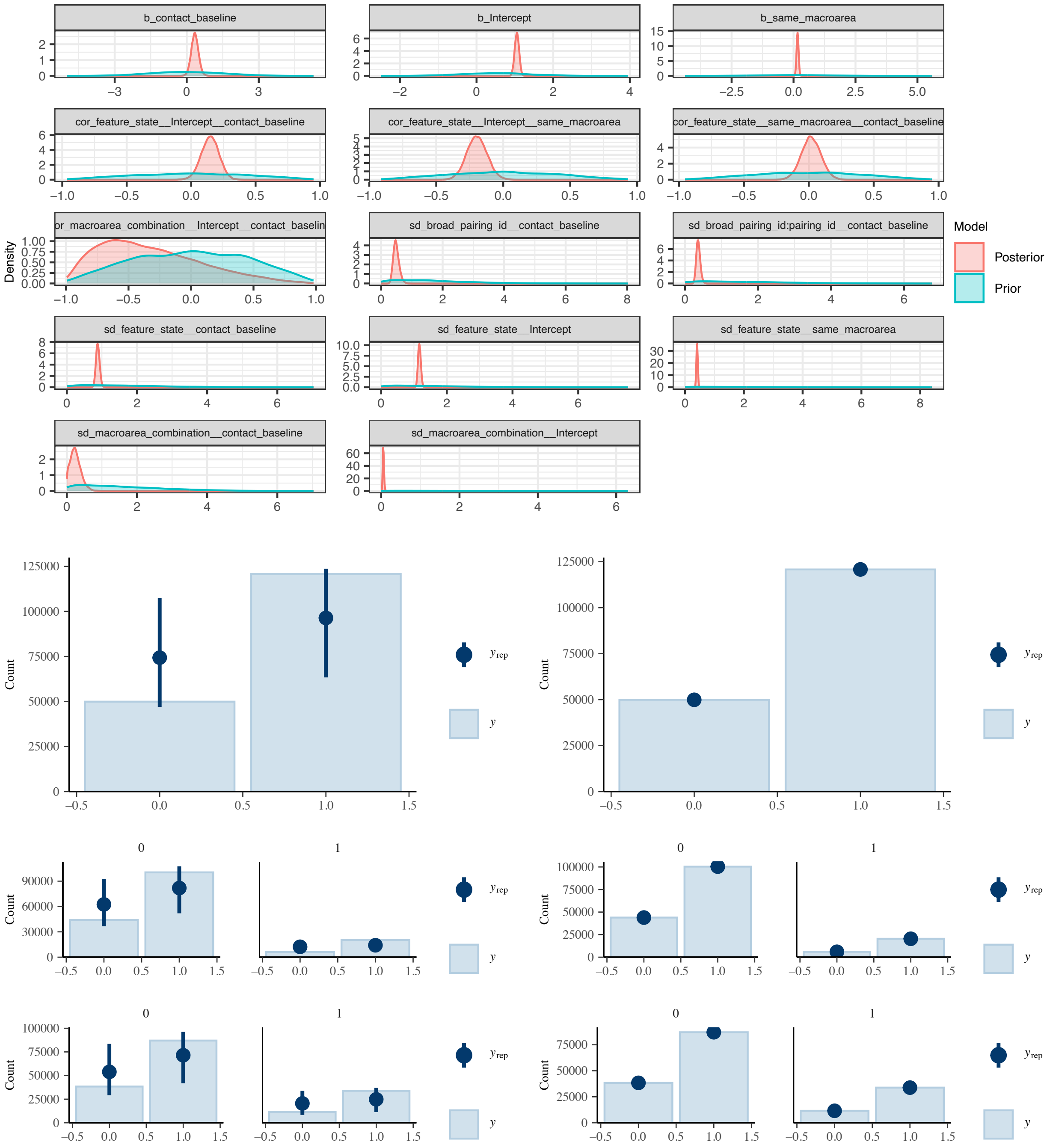

Fig. S24.

Prior and posterior distributions for regression coefficients and multilevel hyperparameters (top) as well as prior predictive checks (bottom left) and posterior predictive checks (bottom right) for regression coefficients: combined model (genetic contact and information on areal co-location), including all pairs, TLI data, sensitivity analysis (Glottolog areas).

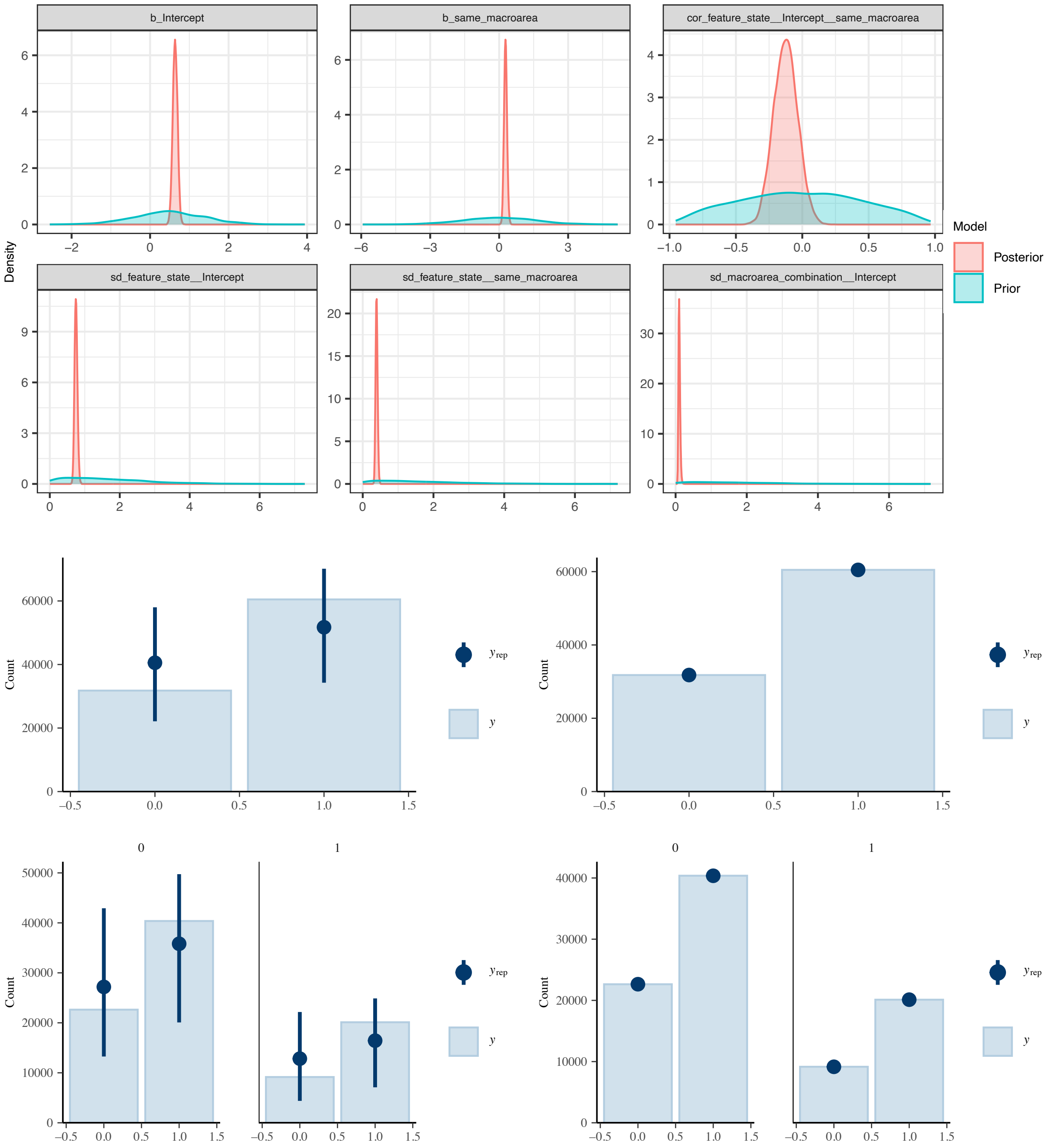

Fig. S25.

Prior and posterior distributions for regression coefficients and multilevel hyperparameters (top) as well as prior predictive checks (bottom left) and posterior predictive checks (bottom right) for regression coefficients: areal model, including all pairs, GBI data, sensitivity analysis (Glottolog areas).

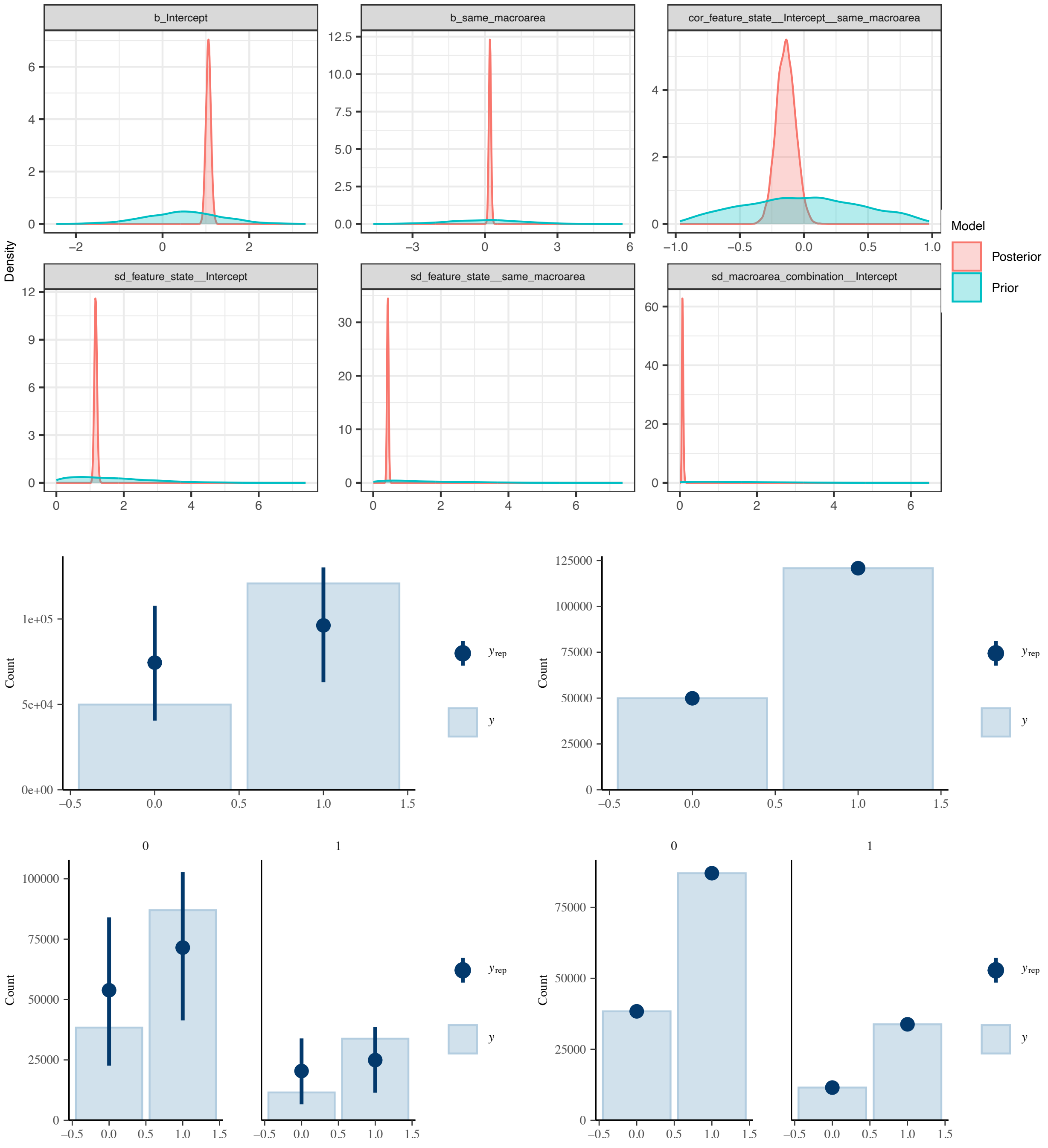

Fig. S26.

Prior and posterior distributions for regression coefficients and multilevel hyperparameters (top) as well as prior predictive checks (bottom left) and posterior predictive checks (bottom right) for regression coefficients: areal model, including all pairs, TLI data, sensitivity analysis (Glottolog areas).

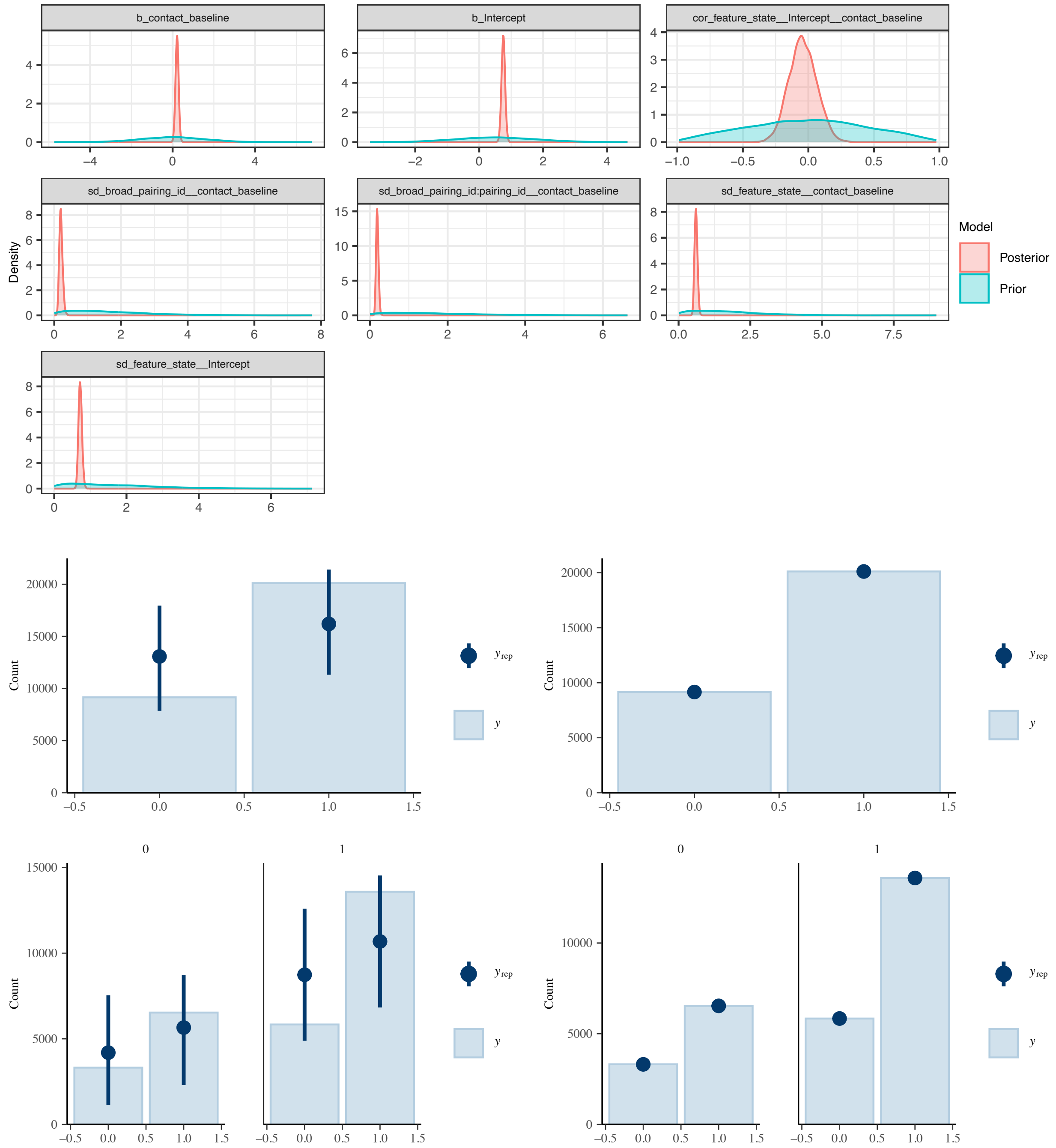

Fig. S27.

Prior and posterior distributions for regression coefficients and multilevel hyperparameters (top) as well as prior predictive checks (bottom left) and posterior predictive checks (bottom right) for regression coefficients: genetic model, including only pairs from the same area, GBI data, sensitivity analysis (Glottolog areas).

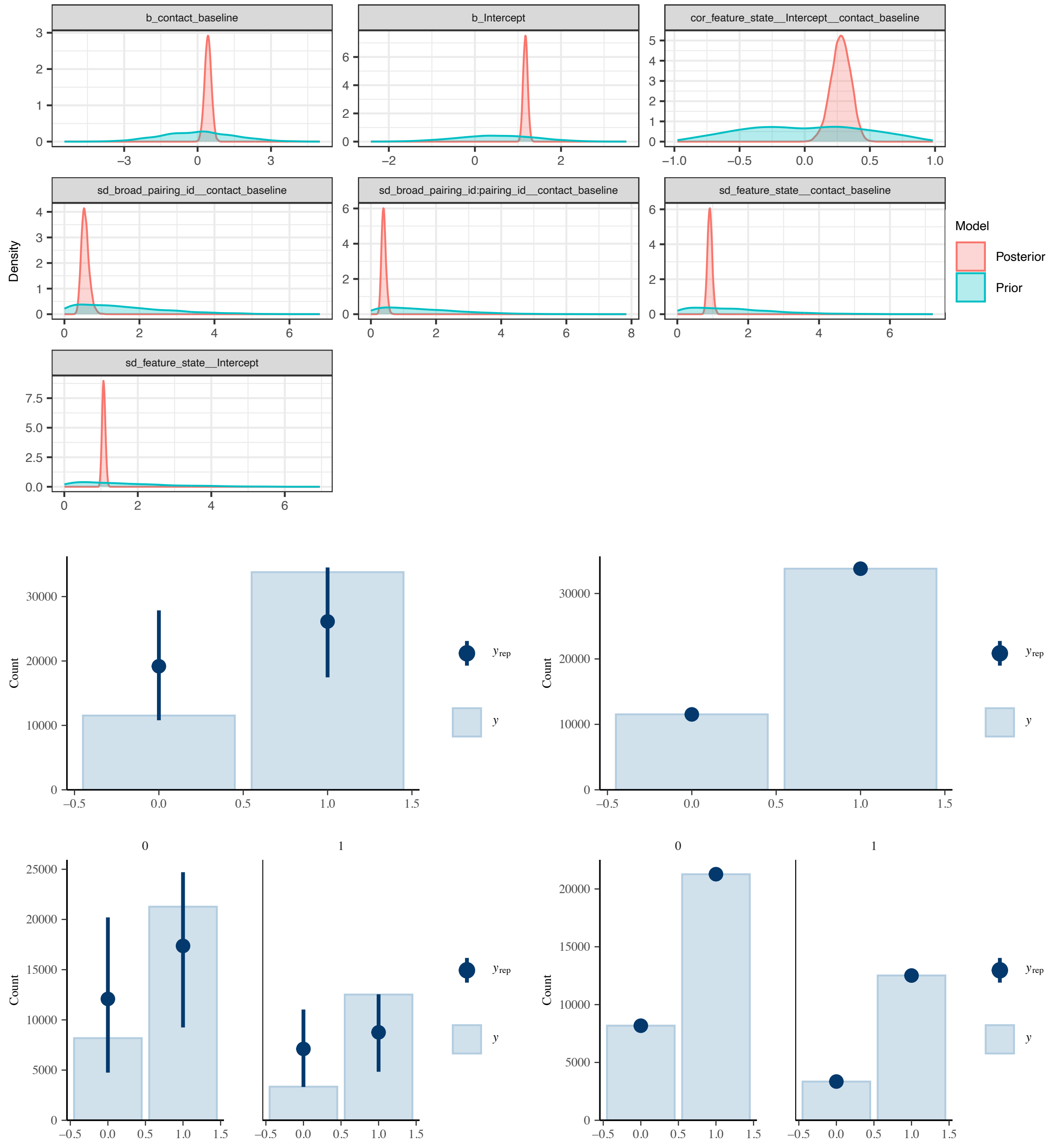

Fig. S28.

Prior and posterior distributions for regression coefficients and multilevel hyperparameters (top) as well as prior predictive checks (bottom left) and posterior predictive checks (bottom right) for regression coefficients: genetic model, including only pairs from the same area, TLI data, sensitivity analysis (Glottolog areas).

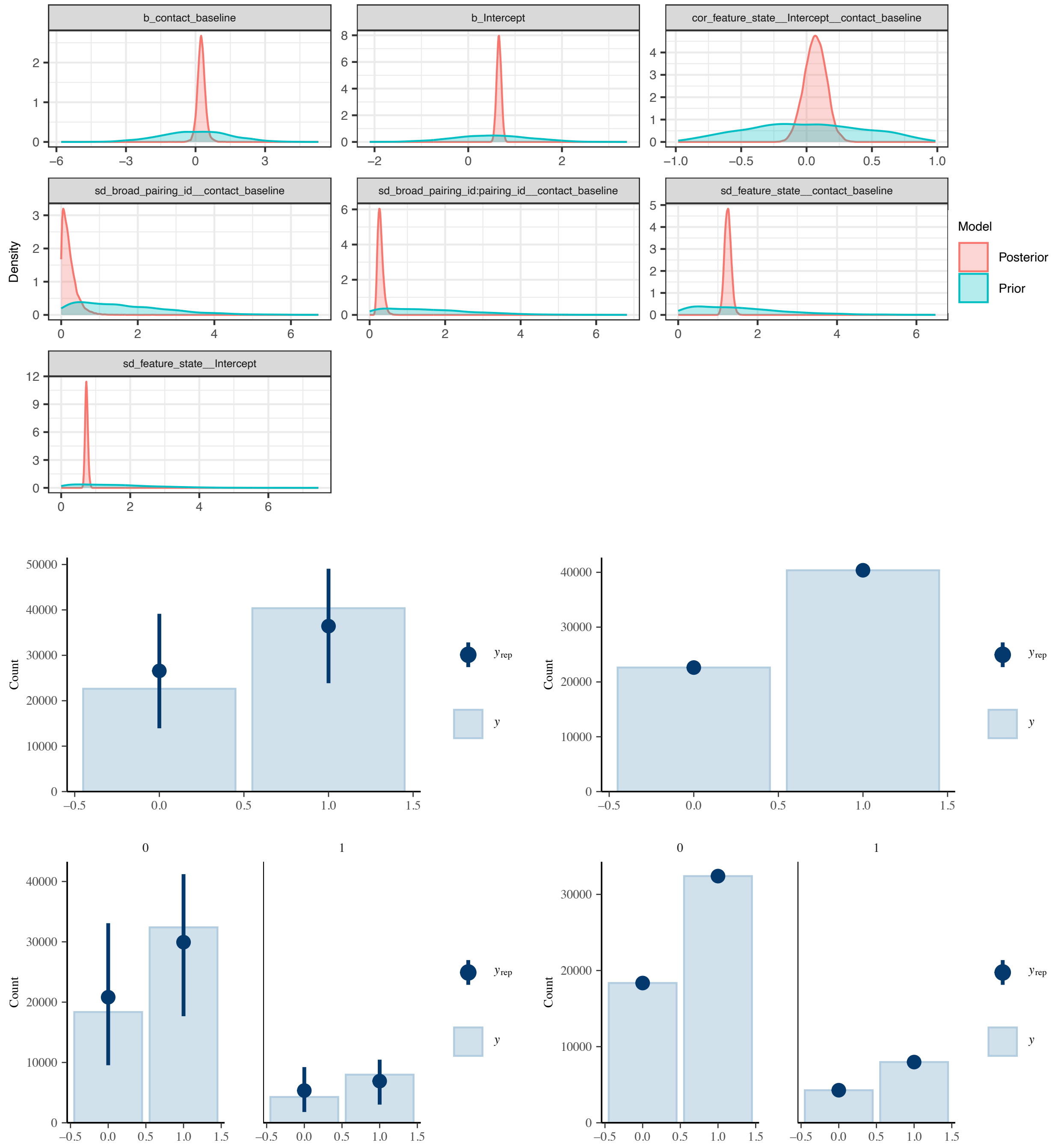

Fig. S29.

Prior and posterior distributions for regression coefficients and multilevel hyperparameters (top) as well as prior predictive checks (bottom left) and posterior predictive checks (bottom right) for regression coefficients: genetic model, including only pairs from different areas, GBI data, sensitivity analysis (Glottolog areas).

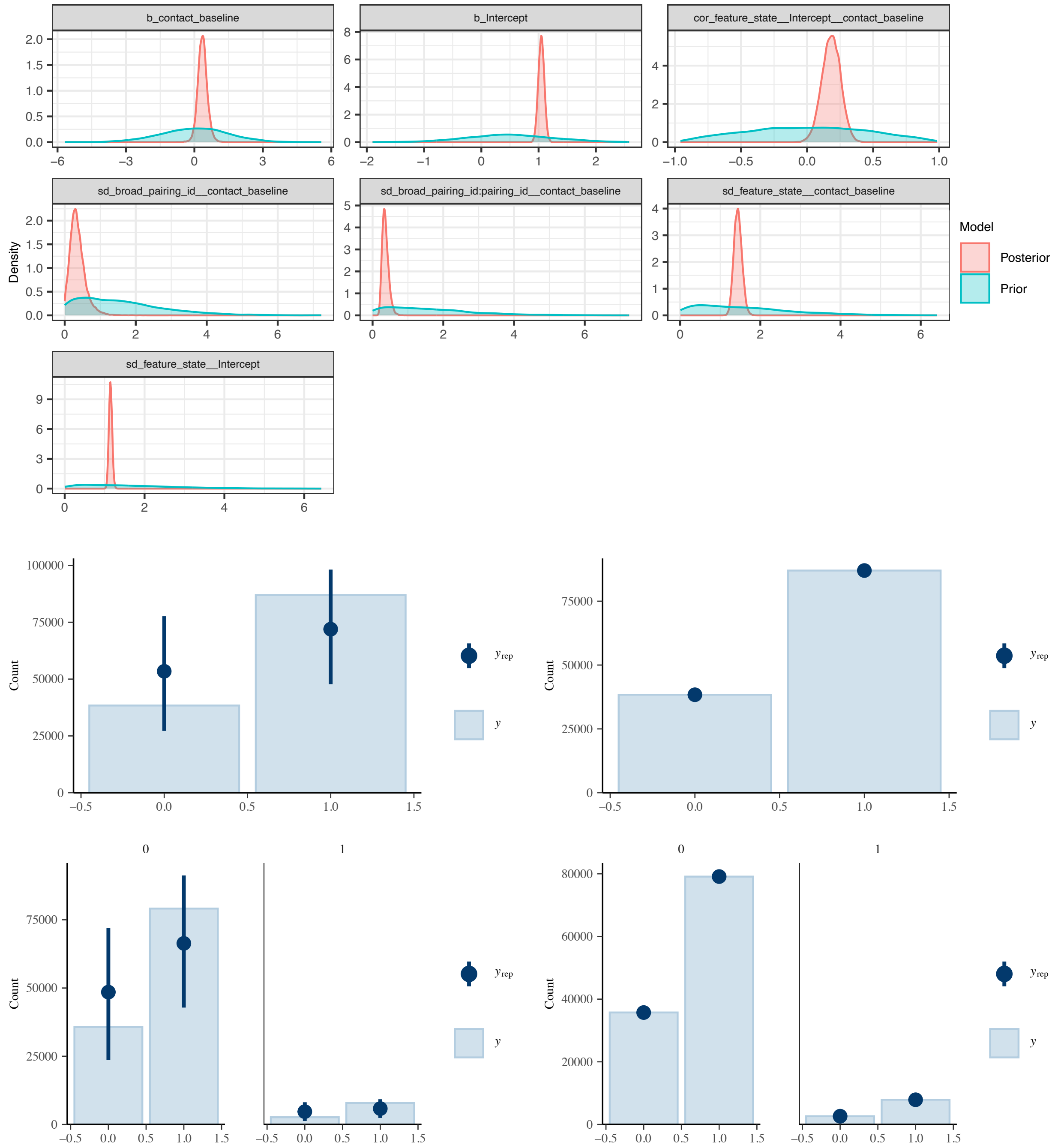

Fig. S30.

Prior and posterior distributions for regression coefficients and multilevel hyperparameters (top) as well as prior predictive checks (bottom left) and posterior predictive checks (bottom right) for regression coefficients: genetic model, including only pairs from different areas, TLI data, sensitivity analysis (Glottolog areas).

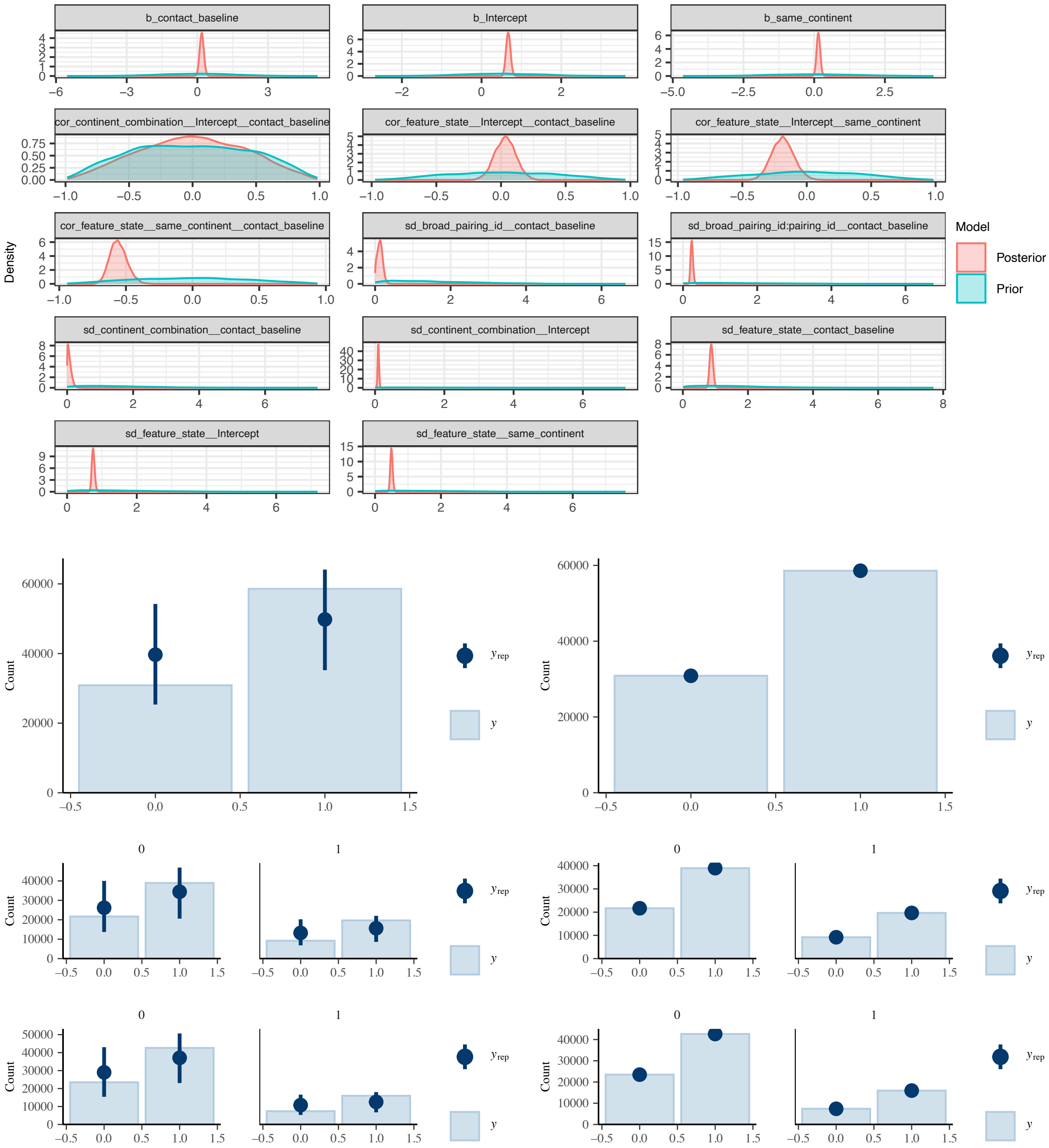

Fig. S31.

Prior and posterior distributions for regression coefficients and multilevel hyperparameters (top) as well as prior predictive checks (bottom left) and posterior predictive checks (bottom right) for regression coefficients: combined model (genetic contact and information on areal co-location), including all pairs from the F_3-sensitivity analysis, GBI data, F_3 -sensitivity analysis (AUTOTYP areas).

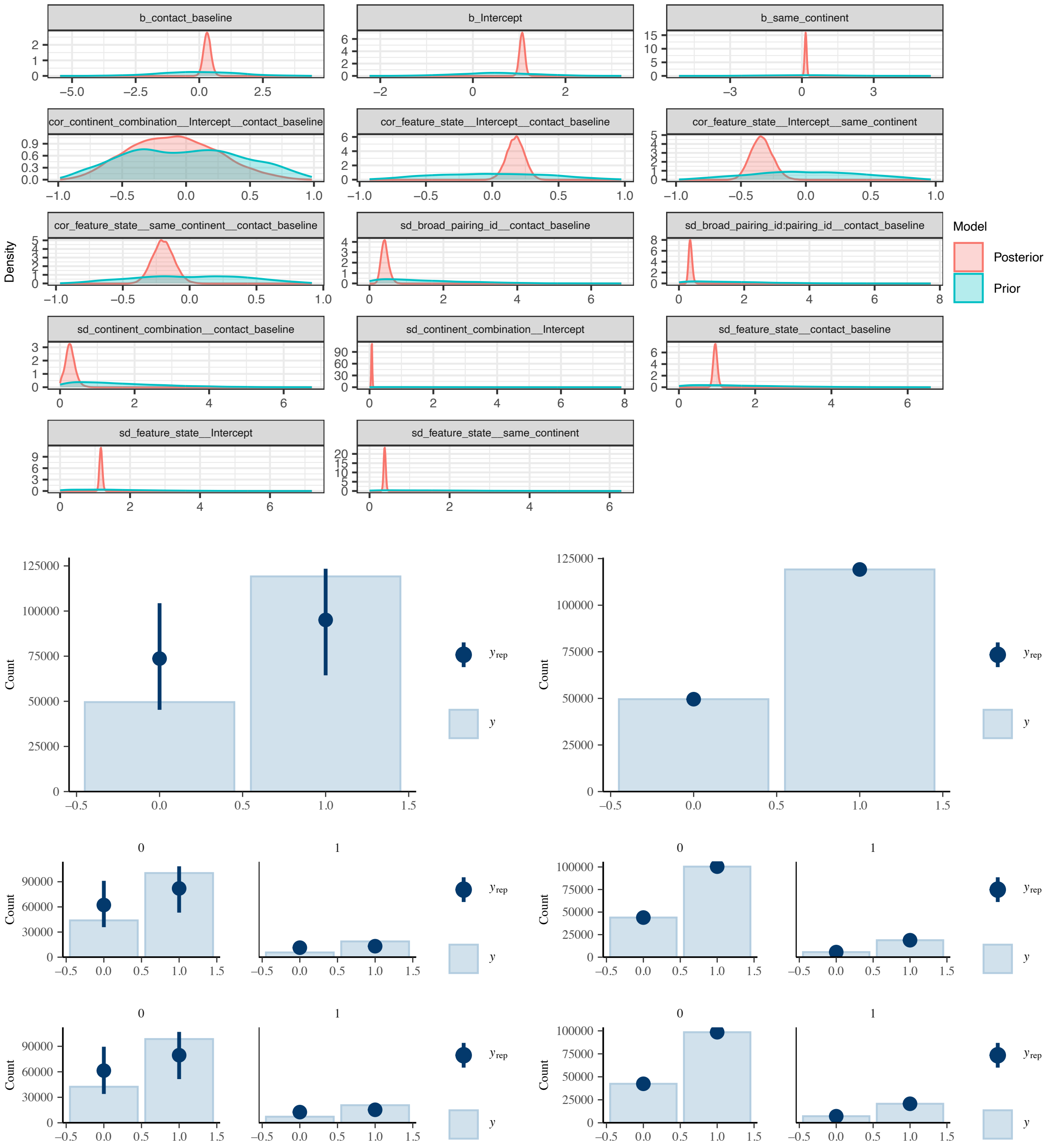

Fig. S32.

Prior and posterior distributions for regression coefficients and multilevel hyperparameters (top) as well as prior predictive checks (bottom left) and posterior predictive checks (bottom right) for regression coefficients: combined model (genetic contact and information on areal co-location), including all pairs from the F_3 -sensitivity analysis, TLI data, F_3 -sensitivity analysis (AUTOTYP areas).

Table S1.
GeLaTo populations used for ADMIXTURE analysis, including number of individuals and language assignments to both datasets used for linguistic analysis.

(external .csv file, tableS1.csv)

Table S2.

List of admixed genetic population pairs, including the reference (GeLaTo or a specified publication) and corresponding linguistic pair sets. Each of the 126 pairs has its unique pair id, which is nested within one of 39 broad pair ids, grouping together contact instances with the same source. The table lists language assignments to targets for GBI and TLI individually, and clade assignments to sources (valid for both GBI and TLI). A single language within the clade is picked as a source for GBI and TLI individually for plotting purposes only (Fig. 1C). For language pairs obtained from GeLaTo-based ADMIXTURE analysis, we also record F_3 -statistics and their Z-scores.

(external .csv file, tableS2.csv)

Table S3.

Features from GBI-statistical and TLI-statistical and their definitions. The third and fourth columns provide the assignments of the feature groups defined in the databases and explained in the main text.

(external .csv file, tableS3.csv)

Table S4.

Summary of fixed and random effects (on log-odds scale) for m1 on the GBI data, using AUTOTYP areas.

m1 (GBI), Fixed Effects Summary

Coefficient	Estimate	Est.Error	l-89% CI	u-89% CI	Rhat	Bulk_ESS	Tail_ESS
α	0.67	0.06	0.58	0.76	1.01	856	1,470
$\beta_1(\mathrm{A})$	0.16	0.06	0.06	0.26	1.00	5,822	8,762
$\beta_2(\mathrm{G})$	0.18	0.08	0.05	0.31	1.00	4,665	7,038

Random Effects, Group: broad_pairing_id

Hyperparameter	Estimate	Est.Error	l-89% CI	u-89% CI	Rhat	Bulk_ESS	Tail_ESS
$\sigma_{\mathrm{BROAD\_ID}}$	0.14	0.06	0.03	0.24	1	2,180	2,526

Random Effects, Group: broad_pairing_id:pairing_id

Hyperparameter	Estimate	Est.Error	l-89% CI	u-89% CI	Rhat	Bulk_ESS	Tail_ESS
$\sigma_{\mathrm{BROAD\_ID/PAIR\_ID}}$	0.22	0.03	0.18	0.27	1	3,884	8,052

Random Effects, Group: continent_combination

Hyperparameter	Estimate	Est.Error	l-89% CI	u-89% CI	Rhat	Bulk_ESS	Tail_ESS
$\sigma_{\alpha_{\mathrm{AA}}}$	0.10	0.02	0.07	0.12	1	5,981	9,814
$\sigma_{\beta_{\mathrm{AA}}}$	0.07	0.06	0.01	0.19	1	3,107	5,425
$cor(\alpha_{\mathrm{AA}}, \beta_{\mathrm{AA}})$	0.05	0.40	-0.62	0.68	1	14,651	10,753

Random Effects, Group: feature_state

Hyperparameter	Estimate	Est.Error	l-89% CI	u-89% CI	Rhat	Bulk_ESS	Tail_ESS
$\sigma_{\alpha_{\mathrm{STATE}}}$	0.75	0.04	0.69	0.82	1	1,828	3,821
$\sigma_{\beta_{\mathrm{A,STATE}}}$	0.51	0.03	0.46	0.57	1	5,848	8,880
$\sigma_{\beta_{\mathrm{G,STATE}}}$	0.82	0.05	0.75	0.90	1	2,994	4,950

Hyperparameter	Estimate	Est.Error	l-89% CI	u-89% CI	Rhat	Bulk_ESS	Tail_ESS
$cor(\alpha_{\mathrm{STATE}}, \beta_{\mathrm{A,STATE}})$	-0.17	0.09	-0.31	-0.04	1	5,694	8,748
$cor(\alpha_{\mathrm{STATE}}, \beta_{\mathrm{G,STATE}})$	0.02	0.08	-0.11	0.15	1	3,623	6,561
$cor(\beta_{\mathrm{A,STATE}}, \beta_{\mathrm{G,STATE}})$	-0.56	0.06	-0.65	-0.46	1	2,105	3,828

Table S5.

Summary of fixed and random effects (on log-odds scale) for m1 on the TLI data, using AUTOTYP areas.

m1 (TLI), Fixed Effects Summary

Coefficient	Estimate	Est.Error	l-89% CI	u-89% CI	Rhat	Bulk_ESS	Tail_ESS
α	1.06	0.05	0.97	1.15	1	657	1,426
$\beta_1(\mathrm{A})$	0.16	0.04	0.10	0.23	1	11,616	14,468
$\beta_2(\mathrm{G})$	0.36	0.15	0.11	0.61	1	9,689	10,611

Random Effects, Group: broad_pairing_id

Hyperparameter	Estimate	Est.Error	l-89% CI	u-89% CI	Rhat	Bulk_ESS	Tail_ESS
$\sigma_{\mathrm{BROAD\_ID}}$	0.47	0.09	0.34	0.63	1	8,188	12,714

Random Effects, Group: broad_pairing_id:pairing_id

Hyperparameter	Estimate	Est.Error	l-89% CI	u-89% CI	Rhat	Bulk_ESS	Tail_ESS
$\sigma_{\mathrm{BROAD\_ID/PAIR\_ID}}$	0.35	0.05	0.28	0.44	1	6,963	12,433

Random Effects, Group: continent_combination

Hyperparameter	Estimate	Est.Error	l-89% CI	u-89% CI	Rhat	Bulk_ESS	Tail_ESS
α_{AA}	0.07	0.01	0.05	0.09	1	8,652	12,848
β_{AA}	0.32	0.14	0.12	0.55	1	4,868	5,201
$cor(\alpha_{\mathrm{AA}}, \beta_{\mathrm{AA}})$	-0.11	0.33	-0.62	0.43	1	12,381	13,232

Random Effects, Group: feature_state

Hyperparameter	Estimate	Est.Error	l-89% CI	u-89% CI	Rhat	Bulk_ESS	Tail_ESS
$\sigma_{\alpha_{\mathrm{STATE}}}$	1.17	0.04	1.11	1.23	1	1,875	3,774
$\sigma_{\beta_{\mathrm{A,STATE}}}$	0.40	0.03	0.36	0.44	1	9,907	14,876
$\sigma_{\beta_{\mathrm{G,STATE}}}$	0.93	0.05	0.85	1.02	1	5,986	10,523

Hyperparameter	Estimate	Est.Error	l-89% CI	u-89% CI	Rhat	Bulk_ESS	Tail_ESS
$cor(\alpha_{\mathrm{STATE}}, \beta_{\mathrm{A,STATE}})$	-0.31	0.08	-0.44	-0.18	1	15,783	15,780
$cor(\alpha_{\mathrm{STATE}}, \beta_{\mathrm{G,STATE}})$	0.17	0.07	0.07	0.28	1	7,971	11,944
$cor(\beta_{\mathrm{A,STATE}}, \beta_{\mathrm{G,STATE}})$	-0.23	0.08	-0.35	-0.11	1	2,329	4,675

Table S6.

Summary of fixed and random effects (on log-odds scale) for m2 on the GBI data, using AUTOTYP areas.

m2 (GBI), Fixed Effects Summary

Coefficient	Estimate	Est.Error	l-89% CI	u-89% CI	Rhat	Bulk_ESS	Tail_ESS
α	0.68	0.06	0.59	0.78	1.01	641	1,075
$\beta_1(\mathrm{A})$	0.25	0.06	0.16	0.35	1.00	4,462	7,397

Random Effects, Group: continent_combination

Hyperparameter	Estimate	Est.Error	l-89% CI	u-89% CI	Rhat	Bulk_ESS	Tail_ESS
σ_{AA}	0.12	0.02	0.09	0.15	1	4,599	8,101

Random Effects, Group: feature_state

Hyperparameter	Estimate	Est.Error	l-89% CI	u-89% CI	Rhat	Bulk_ESS	Tail_ESS
$\sigma_{\alpha_{\mathrm{STATE}}}$	0.76	0.04	0.69	0.82	1	1,612	3,040
$\sigma_{\beta_{\mathrm{STATE}}}$	0.42	0.03	0.37	0.47	1	5,164	8,098
$cor(\alpha_{\mathrm{STATE}}, \beta_{\mathrm{STATE}})$	-0.18	0.09	-0.32	-0.04	1	4,860	8,196

Table S7.

Summary of fixed and random effects (on log-odds scale) for m2 on the TLI data, using AUTOTYP areas.

m2 (TLI), Fixed Effects Summary

Coefficient	Estimate	Est.Error	l-89% CI	u-89% CI	Rhat	Bulk_ESS	Tail_ESS
α	1.07	0.06	0.98	1.16	1	1,123	2,413
$\beta_1(\mathrm{A})$	0.21	0.05	0.13	0.28	1	17,746	21,892

Random Effects, Group: continent_combination

Hyperparameter	Estimate	Est.Error	l-89% CI	u-89% CI	Rhat	Bulk_ESS	Tail_ESS
σ_{AA}	0.1	0.01	0.08	0.12	1	12,232	19,782

Random Effects, Group: feature_state

Hyperparameter	Estimate	Est.Error	l-89% CI	u-89% CI	Rhat	Bulk_ESS	Tail_ESS
$\sigma_{\alpha_{\mathrm{STATE}}}$	1.17	0.04	1.11	1.24	1	2,984	6,116
$\sigma_{\beta_{\mathrm{STATE}}}$	0.41	0.02	0.37	0.45	1	14,751	21,033
$cor(\alpha_{\mathrm{STATE}}, \beta_{\mathrm{STATE}})$	-0.25	0.08	-0.38	-0.12	1	24,507	23,804

Table S8.

Summary of fixed and random effects (on log-odds scale) for m3 on the GBI data, using AUTOTYP areas.

m3 (GBI), Fixed Effects Summary

Coefficient	Estimate	Est.Error	l-89% CI	u-89% CI	Rhat	Bulk_ESS	Tail_ESS
α	0.67	0.05	0.59	0.75	1	768	1,517
$\beta_1(\mathrm{G})$	0.27	0.07	0.15	0.39	1	2,614	4,969

Random Effects, Group: broad_pairing_id

Hyperparameter	Estimate	Est.Error	l-89% CI	u-89% CI	Rhat	Bulk_ESS	Tail_ESS
$\sigma_{\mathrm{BROAD\_ID}}$	0.19	0.05	0.1	0.27	1	2,530	2,782

Random Effects, Group: broad_pairing_id:pairing_id

Hyperparameter	Estimate	Est.Error	l-89% CI	u-89% CI	Rhat	Bulk_ESS	Tail_ESS
$\sigma_{\mathrm{BROAD\_ID/PAIR\_ID}}$	0.23	0.03	0.19	0.28	1	3,805	6,022

Random Effects, Group: feature_state

Hyperparameter	Estimate	Est.Error	l-89% CI	u-89% CI	Rhat	Bulk_ESS	Tail_ESS
$\sigma_{\alpha_{\mathrm{STATE}}}$	0.73	0.04	0.68	0.80	1	1,547	3,405
$\sigma_{\beta_{\mathrm{STATE}}}$	0.72	0.04	0.65	0.79	1	3,036	5,619
$cor(\alpha_{\mathrm{STATE}}, \beta_{\mathrm{STATE}})$	-0.06	0.08	-0.19	0.08	1	2,352	4,154

Table S9.
Summary of fixed and random effects (on log-odds scale) for m3 on the TLI data, using AUTOTYP areas.

m3 (TLI), Fixed Effects Summary

Coefficient	Estimate	Est.Error	l-89% CI	u-89% CI	Rhat	Bulk_ESS	Tail_ESS
α	1.08	0.05	0.99	1.16	1	649	1,637
$\beta_1(\mathrm{G})$	0.44	0.12	0.25	0.63	1	8,350	13,726

Random Effects, Group: broad_pairing_id

Hyperparameter	Estimate	Est.Error	l-89% CI	u-89% CI	Rhat	Bulk_ESS	Tail_ESS
$\sigma_{\mathrm{BROAD\_ID}}$	0.54	0.09	0.4	0.7	1	9,850	15,545

Random Effects, Group: broad_pairing_id:pairing_id

Hyperparameter	Estimate	Est.Error	l-89% CI	u-89% CI	Rhat	Bulk_ESS	Tail_ESS
$\sigma_{\mathrm{BROAD\_ID/PAIR\_ID}}$	0.39	0.05	0.31	0.47	1	9,283	16,254

Random Effects, Group: feature_state

Hyperparameter	Estimate	Est.Error	l-89% CI	u-89% CI	Rhat	Bulk_ESS	Tail_ESS
$\sigma_{\alpha_{\mathrm{STATE}}}$	1.15	0.04	1.09	1.21	1	1,812	4,497
$\sigma_{\beta_{\mathrm{STATE}}}$	0.90	0.05	0.82	0.98	1	8,537	15,728
$cor(\alpha_{\mathrm{STATE}}, \beta_{\mathrm{STATE}})$	0.12	0.07	0.01	0.22	1	9,248	15,241

Table S10.

Summary of fixed and random effects (on log-odds scale) for m4 on the GBI data, using AUTOTYP areas.

m4 (GBI), Fixed Effects Summary

Coefficient	Estimate	Est.Error	l-89% CI	u-89% CI	Rhat	Bulk_ESS	Tail_ESS
α	0.74	0.06	0.65	0.83	1	3,835	4,717
$\beta_1(\mathrm{G})$	0.20	0.08	0.08	0.32	1	5,435	5,744

Random Effects, Group: broad_pairing_id

Hyperparameter	Estimate	Est.Error	l-89% CI	u-89% CI	Rhat	Bulk_ESS	Tail_ESS
$\sigma_{\mathrm{BROAD\_ID}}$	0.21	0.06	0.13	0.3	1	3,037	3,811

Random Effects, Group: broad_pairing_id:pairing_id

Hyperparameter	Estimate	Est.Error	l-89% CI	u-89% CI	Rhat	Bulk_ESS	Tail_ESS
$\sigma_{\mathrm{BROAD\_ID/PAIR\_ID}}$	0.2	0.03	0.16	0.25	1	3,184	4,680

Random Effects, Group: feature_state

Hyperparameter	Estimate	Est.Error	l-89% CI	u-89% CI	Rhat	Bulk_ESS	Tail_ESS
$\sigma_{\alpha_{\mathrm{STATE}}}$	0.71	0.05	0.63	0.79	1	4,275	5,935
$\sigma_{\beta_{\mathrm{STATE}}}$	0.63	0.05	0.55	0.72	1	3,203	5,464
$cor(\alpha_{\mathrm{STATE}}, \beta_{\mathrm{STATE}})$	-0.06	0.11	-0.23	0.11	1	3,651	4,695

Table S11.

Summary of fixed and random effects (on log-odds scale) for m4 on the TLI data, using AUTOTYP areas.

m4 (TLI), Fixed Effects Summary

Coefficient	Estimate	Est.Error	l-89% CI	u-89% CI	Rhat	Bulk_ESS	Tail_ESS
α	1.15	0.05	1.08	1.23	1	2,021	3,666
$\beta_1(\mathrm{G})$	0.43	0.14	0.22	0.66	1	3,351	4,406

Random Effects, Group: broad_pairing_id

Hyperparameter	Estimate	Est.Error	l-89% CI	u-89% CI	Rhat	Bulk_ESS	Tail_ESS
$\sigma_{\mathrm{BROAD\_ID}}$	0.55	0.11	0.4	0.73	1	3,082	5,109

Random Effects, Group: broad_pairing_id:pairing_id

Hyperparameter	Estimate	Est.Error	l-89% CI	u-89% CI	Rhat	Bulk_ESS	Tail_ESS
$\sigma_{\mathrm{BROAD\_ID/PAIR\_ID}}$	0.42	0.07	0.32	0.53	1	2,711	4,140

Random Effects, Group: feature_state

Hyperparameter	Estimate	Est.Error	l-89% CI	u-89% CI	Rhat	Bulk_ESS	Tail_ESS
$\sigma_{\alpha_{\mathrm{STATE}}}$	0.96	0.04	0.90	1.03	1	2,891	4,546
$\sigma_{\beta_{\mathrm{STATE}}}$	0.99	0.07	0.88	1.11	1	3,309	5,251
$cor(\alpha_{\mathrm{STATE}}, \beta_{\mathrm{STATE}})$	0.26	0.08	0.13	0.38	1	3,487	4,811

Table S12.

Summary of fixed and random effects (on log-odds scale) for m5 on the GBI data, using AUTOTYP areas.

m5 (GBI), Fixed Effects Summary

Coefficient	Estimate	Est.Error	l-89% CI	u-89% CI	Rhat	Bulk_ESS	Tail_ESS
α	0.66	0.05	0.58	0.75	1	451	976
$\beta_1(\mathrm{G})$	0.44	0.17	0.18	0.72	1	1,929	3,443

Random Effects, Group: broad_pairing_id

Hyperparameter	Estimate	Est.Error	l-89% CI	u-89% CI	Rhat	Bulk_ESS	Tail_ESS
$\sigma_{\mathrm{BROAD\_ID}}$	0.25	0.18	0.03	0.55	1	1,965	2,624

Random Effects, Group: broad_pairing_id:pairing_id

Hyperparameter	Estimate	Est.Error	l-89% CI	u-89% CI	Rhat	Bulk_ESS	Tail_ESS
$\sigma_{\mathrm{BROAD\_ID/PAIR\_ID}}$	0.33	0.08	0.23	0.47	1	2,972	5,787

Random Effects, Group: feature_state

Hyperparameter	Estimate	Est.Error	l-89% CI	u-89% CI	Rhat	Bulk_ESS	Tail_ESS
$\sigma_{\alpha_{\mathrm{STATE}}}$	0.74	0.04	0.68	0.80	1.01	1,155	2,385
$\sigma_{\beta_{\mathrm{STATE}}}$	1.20	0.08	1.09	1.33	1.00	1,946	4,125
$cor(\alpha_{\mathrm{STATE}}, \beta_{\mathrm{STATE}})$	0.08	0.08	-0.05	0.22	1.00	959	2,068

Table S13.

Summary of fixed and random effects (on log-odds scale) for m5 on the TLI data.

m5 (TLI), Fixed Effects Summary

Coefficient	Estimate	Est.Error	l-89% CI	u-89% CI	Rhat	Bulk_ESS	Tail_ESS
α	1.06	0.05	0.98	1.14	1.01	462	880
$\beta_1(\mathrm{G})$	0.40	0.21	0.06	0.73	1.00	5,850	8,863

Random Effects, Group: broad_pairing_id

Hyperparameter	Estimate	Est.Error	l-89% CI	u-89% CI	Rhat	Bulk_ESS	Tail_ESS
$\sigma_{\mathrm{BROAD\_ID}}$	0.47	0.2	0.23	0.83	1	5,560	8,279

Random Effects, Group: broad_pairing_id:pairing_id

Hyperparameter	Estimate	Est.Error	l-89% CI	u-89% CI	Rhat	Bulk_ESS	Tail_ESS
$\sigma_{\mathrm{BROAD\_ID/PAIR\_ID}}$	0.34	0.07	0.24	0.47	1	5,358	9,593

Random Effects, Group: feature_state

Hyperparameter	Estimate	Est.Error	l-89% CI	u-89% CI	Rhat	Bulk_ESS	Tail_ESS
$\sigma_{\alpha_{\mathrm{STATE}}}$	1.16	0.04	1.10	1.22	1	1,296	2,612
$\sigma_{\beta_{\mathrm{STATE}}}$	1.24	0.08	1.12	1.38	1	4,917	9,436
$cor(\alpha_{\mathrm{STATE}}, \beta_{\mathrm{STATE}})$	0.17	0.07	0.05	0.27	1	4,896	9,415

Table S14.

Summary of fixed and random effects (on log-odds scale) for m1 on the GBI data, using Glottolog areas (sensitivity analysis).

m1 (GBI), Fixed Effects Summary

Coefficient	Estimate	Est.Error	l-89% CI	u-89% CI	Rhat	Bulk_ESS	Tail_ESS
α	0.64	0.06	0.54	0.73	1.01	709	1,207
$\beta_1(\mathrm{A})$	0.18	0.07	0.08	0.30	1.00	4,332	5,307
$\beta_2(\mathrm{G})$	0.15	0.09	0.01	0.29	1.00	3,225	4,796

Random Effects, Group: broad_pairing_id

Hyperparameter	Estimate	Est.Error	l-89% CI	u-89% CI	Rhat	Bulk_ESS	Tail_ESS
$\sigma_{\mathrm{BROAD\_ID}}$	0.12	0.06	0.03	0.22	1	1,350	2,011

Random Effects, Group: broad_pairing_id:pairing_id

Hyperparameter	Estimate	Est.Error	l-89% CI	u-89% CI	Rhat	Bulk_ESS	Tail_ESS
$\sigma_{\mathrm{BROAD\_ID/PAIR\_ID}}$	0.21	0.03	0.17	0.26	1	2,925	4,643

Random Effects, Group: feature_state

Hyperparameter	Estimate	Est.Error	l-89% CI	u-89% CI	Rhat	Bulk_ESS	Tail_ESS
$\sigma_{\alpha_{\mathrm{STATE}}}$	0.75	0.04	0.69	0.81	1	1,298	2,808
$\sigma_{\beta_{\mathrm{A,STATE}}}$	0.45	0.03	0.40	0.50	1	4,258	6,503
$\sigma_{\beta_{\mathrm{G,STATE}}}$	0.79	0.05	0.72	0.87	1	1,870	4,232
$cor(\alpha_{\mathrm{STATE}}, \beta_{\mathrm{A,STATE}})$	-0.12	0.09	-0.26	0.02	1	4,246	6,403
$cor(\alpha_{\mathrm{STATE}}, \beta_{\mathrm{G,STATE}})$	0.00	0.08	-0.13	0.13	1	2,419	4,197
$cor(\beta_{\mathrm{A,STATE}}, \beta_{\mathrm{G,STATE}})$	-0.46	0.07	-0.57	-0.35	1	1,102	2,653

Random Effects, Group: macroarea_combination

Hyperparameter	Estimate	Est.Error	l-89% CI	u-89% CI	Rhat	Bulk_ESS	Tail_ESS
σ_{AA}	0.10	0.02	0.07	0.14	1	3,634	5,218
β_{AA}	0.11	0.08	0.01	0.25	1	2,120	4,624
$cor(\alpha_{\mathrm{AA}}, \beta_{\mathrm{AA}})$	-0.12	0.38	-0.70	0.52	1	8,588	6,991

Table S15.

Summary of fixed and random effects (on log-odds scale) for m1 on the TLI data, using Glottolog areas (sensitivity analysis).

m1 (TLI), Fixed Effects Summary

Coefficient	Estimate	Est.Error	l-89% CI	u-89% CI	Rhat	Bulk_ESS	Tail_ESS
α	1.05	0.06	0.96	1.14	1.01	439	955
$\beta_1(\mathrm{A})$	0.16	0.04	0.10	0.23	1.00	8,074	8,862
$\beta_2(\mathrm{G})$	0.34	0.15	0.10	0.58	1.00	7,101	8,356

Random Effects, Group: broad_pairing_id

Hyperparameter	Estimate	Est.Error	l-89% CI	u-89% CI	Rhat	Bulk_ESS	Tail_ESS
$\sigma_{\mathrm{BROAD\_ID}}$	0.49	0.09	0.36	0.65	1	6,496	9,548

Random Effects, Group: broad_pairing_id:pairing_id

Hyperparameter	Estimate	Est.Error	l-89% CI	u-89% CI	Rhat	Bulk_ESS	Tail_ESS
$\sigma_{\mathrm{BROAD\_ID/PAIR\_ID}}$	0.36	0.05	0.28	0.45	1	4,418	8,381

Random Effects, Group: feature_state

Hyperparameter	Estimate	Est.Error	l-89% CI	u-89% CI	Rhat	Bulk_ESS	Tail_ESS
$\sigma_{\alpha_{\mathrm{STATE}}}$	1.17	0.04	1.10	1.24	1	1,351	3,274
$\sigma_{\beta_{\mathrm{A,STATE}}}$	0.41	0.02	0.37	0.45	1	6,920	9,948
$\sigma_{\beta_{\mathrm{G,STATE}}}$	0.88	0.05	0.80	0.97	1	5,225	8,612
$cor(\alpha_{\mathrm{STATE}}, \beta_{\mathrm{A,STATE}})$	-0.20	0.08	-0.31	-0.08	1	10,747	11,432
$cor(\alpha_{\mathrm{STATE}}, \beta_{\mathrm{G,STATE}})$	0.15	0.07	0.04	0.25	1	6,824	9,629
$cor(\beta_{\mathrm{A,STATE}}, \beta_{\mathrm{G,STATE}})$	0.02	0.07	-0.10	0.14	1	2,134	4,306

Random Effects, Group: macroarea_combination

Hyperparameter	Estimate	Est.Error	l-89% CI	u-89% CI	Rhat	Bulk_ESS	Tail_ESS
σ_{AA}	0.06	0.01	0.04	0.08	1	5,593	9,021
β_{AA}	0.25	0.15	0.04	0.52	1	3,033	4,720
$cor(\alpha_{\mathrm{AA}}, \beta_{\mathrm{AA}})$	-0.32	0.40	-0.86	0.42	1	10,686	9,958

Table S16.

Summary of fixed and random effects (on log-odds scale) for m2 on the GBI data, using Glottolog areas (sensitivity analysis).

m2 (GBI), Fixed Effects Summary

Coefficient	Estimate	Est.Error	l-89% CI	u-89% CI	Rhat	Bulk_ESS	Tail_ESS
α	0.64	0.06	0.54	0.73	1.01	773	1,914
$\beta_1(\mathrm{A})$	0.28	0.07	0.18	0.38	1.00	5,363	7,845

Random Effects, Group: feature_state

Hyperparameter	Estimate	Est.Error	l-89% CI	u-89% CI	Rhat	Bulk_ESS	Tail_ESS
$\sigma_{\alpha_{\mathrm{STATE}}}$	0.75	0.04	0.69	0.81	1	1,669	3,376
$\sigma_{\beta_{\mathrm{STATE}}}$	0.39	0.03	0.35	0.44	1	5,687	9,035
$cor(\alpha_{\mathrm{STATE}}, \beta_{\mathrm{STATE}})$	-0.12	0.09	-0.27	0.02	1	6,791	9,776

Random Effects, Group: macroarea_combination

Hyperparameter	Estimate	Est.Error	l-89% CI	u-89% CI	Rhat	Bulk_ESS	Tail_ESS
σ_{AA}	0.1	0.02	0.07	0.14	1	5,267	8,570

Table S17.

Summary of fixed and random effects (on log-odds scale) for m2 on the TLI data, using Glottolog areas (sensitivity analysis).

m2 (TLI), Fixed Effects Summary

Coefficient	Estimate	Est.Error	l-89% CI	u-89% CI	Rhat	Bulk_ESS	Tail_ESS
α	1.06	0.06	0.97	1.15	1.01	807	1,980
$\beta_1(\mathrm{A})$	0.21	0.05	0.13	0.28	1.00	10,752	12,841

Random Effects, Group: feature_state

Hyperparameter	Estimate	Est.Error	l-89% CI	u-89% CI	Rhat	Bulk_ESS	Tail_ESS
$\sigma_{\alpha_{\mathrm{STATE}}}$	1.17	0.04	1.10	1.23	1	1,878	3,968
$\sigma_{\beta_{\mathrm{STATE}}}$	0.43	0.02	0.40	0.47	1	9,313	14,525
$cor(\alpha_{\mathrm{STATE}}, \beta_{\mathrm{STATE}})$	-0.14	0.07	-0.26	-0.02	1	14,142	15,217

Random Effects, Group: macroarea_combination

Hyperparameter	Estimate	Est.Error	l-89% CI	u-89% CI	Rhat	Bulk_ESS	Tail_ESS
σ_{AA}	0.07	0.02	0.05	0.1	1	7,606	12,410

Table S18.

Summary of fixed and random effects (on log-odds scale) for m4 on the GBI data, using Glottolog areas (sensitivity analysis).

m4 (GBI), Fixed Effects Summary

Coefficient	Estimate	Est.Error	l-89% CI	u-89% CI	Rhat	Bulk_ESS	Tail_ESS
α	0.75	0.06	0.67	0.84	1	2,758	3,842
$\beta_1(\mathrm{G})$	0.22	0.07	0.10	0.33	1	5,464	4,817

Random Effects, Group: broad_pairing_id

Hyperparameter	Estimate	Est.Error	l-89% CI	u-89% CI	Rhat	Bulk_ESS	Tail_ESS
$\sigma_{\mathrm{BROAD\_ID}}$	0.2	0.05	0.13	0.29	1	3,438	4,284

Random Effects, Group: broad_pairing_id:pairing_id

Hyperparameter	Estimate	Est.Error	l-89% CI	u-89% CI	Rhat	Bulk_ESS	Tail_ESS
$\sigma_{\mathrm{BROAD\_ID/PAIR\_ID}}$	0.19	0.03	0.14	0.24	1	3,824	5,520

Random Effects, Group: feature_state

Hyperparameter	Estimate	Est.Error	l-89% CI	u-89% CI	Rhat	Bulk_ESS	Tail_ESS
$\sigma_{\alpha_{\mathrm{STATE}}}$	0.72	0.05	0.65	0.80	1	3,860	5,624
$\sigma_{\beta_{\mathrm{STATE}}}$	0.61	0.05	0.53	0.69	1	3,949	5,416
$cor(\alpha_{\mathrm{STATE}}, \beta_{\mathrm{STATE}})$	-0.04	0.10	-0.21	0.13	1	3,686	5,243

Table S19.

Summary of fixed and random effects (on log-odds scale) for m4 on the TLI data, using Glottolog areas (sensitivity analysis).

m4 (TLI), Fixed Effects Summary

Coefficient	Estimate	Est.Error	l-89% CI	u-89% CI	Rhat	Bulk_ESS	Tail_ESS
α	1.17	0.05	1.09	1.26	1.01	666	1,595
$\beta_1(\mathrm{G})$	0.43	0.14	0.21	0.64	1.00	2,672	3,921

Random Effects, Group: broad_pairing_id

Hyperparameter	Estimate	Est.Error	l-89% CI	u-89% CI	Rhat	Bulk_ESS	Tail_ESS
$\sigma_{\mathrm{BROAD\_ID}}$	0.56	0.1	0.41	0.74	1	2,924	4,294

Random Effects, Group: broad_pairing_id:pairing_id

Hyperparameter	Estimate	Est.Error	l-89% CI	u-89% CI	Rhat	Bulk_ESS	Tail_ESS
$\sigma_{\mathrm{BROAD\_ID/PAIR\_ID}}$	0.4	0.07	0.3	0.51	1	2,585	4,607

Random Effects, Group: feature_state

Hyperparameter	Estimate	Est.Error	l-89% CI	u-89% CI	Rhat	Bulk_ESS	Tail_ESS
$\sigma_{\alpha_{\mathrm{STATE}}}$	1.07	0.04	1.00	1.15	1	1,499	3,117
$\sigma_{\beta_{\mathrm{STATE}}}$	0.92	0.06	0.82	1.02	1	3,175	4,924
$cor(\alpha_{\mathrm{STATE}}, \beta_{\mathrm{STATE}})$	0.28	0.08	0.16	0.39	1	3,440	4,881

Table S20.

Summary of fixed and random effects (on log-odds scale) for m5 on the GBI data, using Glottolog areas (sensitivity analysis).

m5 (GBI), Fixed Effects Summary

Coefficient	Estimate	Est.Error	l-89% CI	u-89% CI	Rhat	Bulk_ESS	Tail_ESS
α	0.66	0.05	0.58	0.74	1.01	523	1,320
$\beta_1(\mathrm{G})$	0.25	0.18	-0.01	0.52	1.00	2,712	3,861

Random Effects, Group: broad_pairing_id

Hyperparameter	Estimate	Est.Error	l-89% CI	u-89% CI	Rhat	Bulk_ESS	Tail_ESS
$\sigma_{\mathrm{BROAD\_ID}}$	0.21	0.2	0.02	0.56	1	2,369	4,552

Random Effects, Group: broad_pairing_id:pairing_id

Hyperparameter	Estimate	Est.Error	l-89% CI	u-89% CI	Rhat	Bulk_ESS	Tail_ESS
$\sigma_{\mathrm{BROAD\_ID/PAIR\_ID}}$	0.28	0.07	0.19	0.41	1	4,586	7,336

Random Effects, Group: feature_state

Hyperparameter	Estimate	Est.Error	l-89% CI	u-89% CI	Rhat	Bulk_ESS	Tail_ESS
$\sigma_{\alpha_{\mathrm{STATE}}}$	0.73	0.04	0.67	0.80	1.01	1,288	3,057
$\sigma_{\beta_{\mathrm{STATE}}}$	1.25	0.08	1.13	1.38	1.00	2,400	4,731
$cor(\alpha_{\mathrm{STATE}}, \beta_{\mathrm{STATE}})$	0.07	0.08	-0.07	0.20	1.00	1,467	2,584

Table S21.

Summary of fixed and random effects (on log-odds scale) for m5 on the TLI data, using Glottolog areas (sensitivity analysis).

m5 (TLI), Fixed Effects Summary

Coefficient	Estimate	Est.Error	l-89% CI	u-89% CI	Rhat	Bulk_ESS	Tail_ESS
α	1.05	0.05	0.96	1.13	1.01	574	1,152
$\beta_1(\mathrm{G})$	0.36	0.21	0.04	0.69	1.00	7,950	10,043

Random Effects, Group: broad_pairing_id

Hyperparameter	Estimate	Est.Error	l-89% CI	u-89% CI	Rhat	Bulk_ESS	Tail_ESS
$\sigma_{\mathrm{BROAD\_ID}}$	0.37	0.22	0.08	0.75	1	4,153	4,547

Random Effects, Group: broad_pairing_id:pairing_id

Hyperparameter	Estimate	Est.Error	l-89% CI	u-89% CI	Rhat	Bulk_ESS	Tail_ESS
$\sigma_{\mathrm{BROAD\_ID/PAIR\_ID}}$	0.36	0.09	0.24	0.52	1	5,511	10,558

Random Effects, Group: feature_state

Hyperparameter	Estimate	Est.Error	l-89% CI	u-89% CI	Rhat	Bulk_ESS	Tail_ESS
$\sigma_{\alpha_{\mathrm{STATE}}}$	1.15	0.04	1.08	1.21	1	1,474	3,655
$\sigma_{\beta_{\mathrm{STATE}}}$	1.44	0.10	1.29	1.61	1	6,033	11,653
$cor(\alpha_{\mathrm{STATE}}, \beta_{\mathrm{STATE}})$	0.18	0.07	0.07	0.29	1	5,560	9,349

Table S22.

Summary of fixed and random effects (on log-odds scale) for m1 on the GBI data, using AUTOTYP areas, but excluding language pairs which fail to show additional evidence for admixture (F_3-sensitivity analysis).

m1 (GBI), Fixed Effects Summary

Coefficient	Estimate	Est.Error	l-89% CI	u-89% CI	Rhat	Bulk_ESS	Tail_ESS
α	0.67	0.05	0.58	0.76	1	580	1,512
$\beta_1(\mathrm{A})$	0.15	0.06	0.05	0.25	1	4,020	7,129
$\beta_2(\mathrm{G})$	0.18	0.09	0.05	0.33	1	3,088	5,489

Random Effects, Group: broad_pairing_id

Hyperparameter	Estimate	Est.Error	l-89% CI	u-89% CI	Rhat	Bulk_ESS	Tail_ESS
$\sigma_{\mathrm{BROAD\_ID}}$	0.13	0.07	0.02	0.24	1	1,738	3,020

Random Effects, Group: broad_pairing_id:pairing_id

Hyperparameter	Estimate	Est.Error	l-89% CI	u-89% CI	Rhat	Bulk_ESS	Tail_ESS
$\sigma_{\mathrm{BROAD\_ID/PAIR\_ID}}$	0.24	0.03	0.19	0.29	1	3,519	7,388

Random Effects, Group: continent_combination

Hyperparameter	Estimate	Est.Error	l-89% CI	u-89% CI	Rhat	Bulk_ESS	Tail_ESS
$\sigma_{\alpha_{\mathrm{AA}}}$	0.10	0.02	0.07	0.12	1	4,958	8,538
$\sigma_{\beta_{\mathrm{AA}}}$	0.08	0.06	0.01	0.19	1	2,996	5,807
$cor(\alpha_{\mathrm{AA}}, \beta_{\mathrm{AA}})$	0.00	0.41	-0.66	0.66	1	9,548	8,942

Random Effects, Group: feature_state

Hyperparameter	Estimate	Est.Error	l-89% CI	u-89% CI	Rhat	Bulk_ESS	Tail_ESS
$\sigma_{\alpha_{\mathrm{STATE}}}$	0.75	0.04	0.69	0.82	1	1,324	2,907
$\sigma_{\beta_{\mathrm{A,STATE}}}$	0.50	0.04	0.45	0.56	1	4,355	7,085

Hyperparameter	Estimate	Est.Error	l-89% CI	u-89% CI	Rhat	Bulk_ESS	Tail_ESS
$\sigma_{\beta_{\mathrm{G,STATE}}}$	0.88	0.05	0.80	0.96	1	2,077	5,139
$cor(\alpha_{\mathrm{STATE}}, \beta_{\mathrm{A,STATE}})$	-0.19	0.09	-0.32	-0.05	1	4,409	7,231
$cor(\alpha_{\mathrm{STATE}}, \beta_{\mathrm{G,STATE}})$	0.03	0.08	-0.10	0.16	1	2,686	5,127
$cor(\beta_{\mathrm{A,STATE}}, \beta_{\mathrm{G,STATE}})$	-0.56	0.06	-0.65	-0.45	1	1,455	3,426

Table S23.

Summary of fixed and random effects (on log-odds scale) for m1 on the TLI data, using AUTOTYP areas, but excluding language pairs which fail to show additional evidence for admixture (F_3 -sensitivity analysis).

m1 (TLI), Fixed Effects Summary

Coefficient	Estimate	Est.Error	l-89% CI	u-89% CI	Rhat	Bulk_ESS	Tail_ESS
α	1.06	0.06	0.97	1.15	1.01	533	934
$\beta_1(\mathrm{A})$	0.16	0.04	0.09	0.22	1.00	8,450	13,353
$\beta_2(\mathrm{G})$	0.31	0.15	0.09	0.55	1.00	8,096	11,480

Random Effects, Group: broad_pairing_id

Hyperparameter	Estimate	Est.Error	l-89% CI	u-89% CI	Rhat	Bulk_ESS	Tail_ESS
$\sigma_{\mathrm{BROAD\_ID}}$	0.42	0.1	0.27	0.59	1	5,982	8,811

Random Effects, Group: broad_pairing_id:pairing_id

Hyperparameter	Estimate	Est.Error	l-89% CI	u-89% CI	Rhat	Bulk_ESS	Tail_ESS
$\sigma_{\mathrm{BROAD\_ID/PAIR\_ID}}$	0.35	0.05	0.27	0.44	1	6,375	11,604

Random Effects, Group: continent_combination

Hyperparameter	Estimate	Est.Error	l-89% CI	u-89% CI	Rhat	Bulk_ESS	Tail_ESS
$\sigma_{\alpha_{\mathrm{AA}}}$	0.07	0.01	0.05	0.09	1	8,192	13,083
$\sigma_{\beta_{\mathrm{AA}}}$	0.28	0.13	0.07	0.50	1	3,858	3,416
$cor(\alpha_{\mathrm{AA}}, \beta_{\mathrm{AA}})$	-0.09	0.34	-0.62	0.47	1	10,407	11,906

Random Effects, Group: feature_state

Hyperparameter	Estimate	Est.Error	l-89% CI	u-89% CI	Rhat	Bulk_ESS	Tail_ESS
$\sigma_{\alpha_{\mathrm{STATE}}}$	1.17	0.04	1.10	1.23	1	1,364	3,706
$\sigma_{\beta_{\mathrm{A,STATE}}}$	0.38	0.03	0.34	0.42	1	8,768	12,967

Hyperparameter	Estimate	Est.Error	l-89% CI	u-89% CI	Rhat	Bulk_ESS	Tail_ESS
$\sigma_{\beta_{\mathrm{G,STATE}}}$	0.96	0.05	0.87	1.04	1	5,921	11,446
$cor(\alpha_{\mathrm{STATE}}, \beta_{\mathrm{A,STATE}})$	-0.34	0.08	-0.47	-0.21	1	13,261	14,393
$cor(\alpha_{\mathrm{STATE}}, \beta_{\mathrm{G,STATE}})$	0.18	0.07	0.07	0.28	1	6,423	10,427
$cor(\beta_{\mathrm{A,STATE}}, \beta_{\mathrm{G,STATE}})$	-0.20	0.08	-0.32	-0.07	1	1,435	3,859

Table S24.

Contact effects on state sharing for the main analysis, using AUTOTYP areas. The table lists, for every feature state and every model (indicating the type of contact: genetic contact: all pairs; genetic contact: different area pairs only; genetic contact: same area pairs only; same area (AUTOTYP)), the mean baseline, and the mean difference in state sharing probabilities under contact as compared to the corresponding baseline. As an alternative transformation, it also lists the mean relative risk of these probabilities. Lower and upper 89%-HPDI values are also provided for each baseline, difference and relative risk, as well as a binary variable indicating whether the 89%-HPDI includes zero (for differences) or one (for relative risk).

(external .csv file, tableS24.csv)

Table S25.

Contact effects on state sharing for the sensitivity analysis, using Glottolog areas. The table lists, for every feature state and every model (indicating the type of contact: genetic contact: all pairs; genetic contact: different area pairs only; genetic contact: same area pairs only; same area (Glottolog)), the mean baseline, and the mean difference in state sharing probabilities under contact as compared to the corresponding baseline. As an alternative transformation, it also lists the mean relative risk of these probabilities. Lower and upper 89%-HPDI values are also provided for each baseline, difference and relative risk, as well as a binary variable indicating whether the 89%-HPDI includes zero (for differences) or one (for relative risk).

(external .csv file, tableS25.csv)
